# Digital Health Implementation Strategies Coproduced With Adults With Acquired Brain Injury, Their Close Others, and Clinicians: Mixed Methods Study With Collaborative Autoethnography and Network Analysis

**DOI:** 10.2196/46396

**Published:** 2023-09-19

**Authors:** Melissa Miao, Rosemary Morrow, Alexander Salomon, Ben Mcculloch, Jean-Christophe Evain, Meg Rebecca Wright, Marie Therese Murphy, Monica Welsh, Liz Williams, Emma Power, Rachael Rietdijk, Deborah Debono, Melissa Brunner, Leanne Togher

**Affiliations:** 1 Graduate School of Health Faculty of Health University of Technology Sydney Sydney Australia; 2 Stakeholder with living experience of acquired brain injury Sydney Australia; 3 Acquired Brain Injury Rehabilitation Ward Caulfield Hospital Alfred Health Network Melbourne Australia; 4 Stakeholder with living experience of acquired brain injury Melbourne Australia; 5 Stakeholder with living experience of acquired brain injury Blenheim Australia; 6 Stakeholder with living experience as a communication partner of a person with acquired brain injury Sydney Australia; 7 Faculty of Education Western Sydney University Sydney Australia; 8 Faculty of Education and Social Work The University of Sydney Sydney Australia; 9 Brain Injury Rehabilitation Unit South Australian Brain Injury Rehabilitation Service Adelaide Australia; 10 Brain Injury Rehabilitation Community and Home (BIRCH) South Australian Brain Injury Rehabilitation Service Adelaide Australia; 11 Faculty of Medicine and Health The University of Sydney Sydney Australia; 12 School of Public Health Faculty of Health University of Technology Sydney Sydney Australia

**Keywords:** complexity, implementation science, internet interventions, brain injury, stroke, traumatic brain injury, delivery of health care, caregivers, digital health, psychosocial interventions, psychosocial, mobile phone

## Abstract

**Background:**

Acquired brain injuries (ABIs), such as stroke and traumatic brain injury, commonly cause cognitive-communication disorders, in which underlying cognitive difficulties also impair communication. As communication is an exchange with others, close others such as family and friends also experience the impact of cognitive-communication impairment. It is therefore an internationally recommended best practice for speech-language pathologists to provide communication support to both people with ABI and the people who communicate with them. Current research also identifies a need for neurorehabilitation professionals to support digital communication, such as social media use, after ABI. However, with >135 million people worldwide affected by ABI, alternate and supplementary service delivery models are needed to meet these communication needs. The “Social Brain Toolkit” is a novel suite of 3 interventions to deliver communication rehabilitation via the internet. However, digital health implementation is complex, and minimal guidance exists for ABI.

**Objective:**

This study aimed to support the implementation of the Social Brain Toolkit by coproducing implementation knowledge with people with ABI, people who communicate with people with ABI, clinicians, and leaders in digital health implementation.

**Methods:**

A maximum variation sample (N=35) of individuals with living experience of ABI, close others, clinicians, and digital health implementation leaders participated in an explanatory sequential mixed methods design. Stakeholders quantitatively prioritized 4 of the 7 theoretical domains of the Nonadoption, Abandonment, Scale-up, Spread, and Sustainability (NASSS) framework as being the most important for Social Brain Toolkit implementation. Qualitative interview and focus group data collection focused on these 4 domains. Data were deductively analyzed against the NASSS framework with stakeholder coauthors to determine implementation considerations and strategies. A collaborative autoethnography of the research was conducted. Interrelationships between considerations and strategies were identified through a post hoc network analysis.

**Results:**

Across the 4 prioritized domains of “condition,” “technology,” “value proposition,” and “adopters,” 48 digital health implementation considerations and 52 tailored developer and clinician implementation strategies were generated. Benefits and challenges of coproduction were identified. The post hoc network analysis revealed 172 unique relationships between the identified implementation considerations and strategies, with user and persona testing and responsive design identified as the potentially most impactful strategies.

**Conclusions:**

People with ABI, close others, clinicians, and digital health leaders coproduced new knowledge of digital health implementation considerations for adults with ABI and the people who communicate with them, as well as tailored implementation strategies. Complexity-informed network analyses offered a data-driven method to identify the 2 most potentially impactful strategies. Although the study was limited by a focus on 4 NASSS domains and the underrepresentation of certain demographics, the wealth of actionable implementation knowledge produced supports future coproduction of implementation research with mutually beneficial outcomes for stakeholders and researchers.

**International Registered Report Identifier (IRRID):**

RR2-10.2196/35080

## Introduction

### Background

Acquired brain injury (ABI), including stroke and traumatic brain injury (TBI), is a globally prevalent condition affecting >135 million people [1]. ABI represents a significant and growing global burden of disease [2,3] with a high need for rehabilitation service support [1].

Cognitive-communication disorders are common sequelae of ABI [4,5]. Cognitive-communication disorders arise when underlying impairments in cognitive skills, such as memory and organization, manifest in a person’s communication [6]. Evidence-based treatment for these communication difficulties is provided by speech-language pathologists [5]. Author RM recalls the following from her living experience:

During my months in hospital, I discovered the critical role speech therapists have in helping people who acquire TBI get back some semblance of normality. As well as losing all my power of speech, my cognition was truly scrambled. So not only did the speech therapist help me regain the ability to speak, but also to reason.

As communication is a bilateral and multilateral exchange between “communication partners,” close others such as friends and family can also be affected by communication changes after ABI. For example, if a person with ABI experiences difficulty staying on topic or remembering details to include during conversations, their communication partners have been found to adjust their own communication to compensate for these communication behaviors [7]. In doing so, communication partners can positively or detrimentally affect the communication rehabilitation of the person with ABI [7-9]. The protocol for this study included a firsthand account of potentially negative impacts of a close other’s sense of disempowerment during communication after ABI [10]. Conversely, author MTM recalls positive experiences of being able to support a friend:

The diminishment of Rosey’s speech was hard on her—she explains and expresses her world through speech. I saw Rosey at least weekly and like Rosey I have a background in special education, so I felt I had some knowledge of supporting others. I think the best support I gave Rosey was feedback—not when we were talking as friends do, but after. I understand the value of data and by being able to tell her explicitly the gains I could see she was making in between each visit, I could affirm her and support her in a really positive way.

Communication changes after ABI can have pervasive and long-term psychosocial effects on both the person with ABI and their close others. As a result of communication changes after ABI, people with ABI can experience negative impacts on their social participation [11,12], relationships [12,13], employment [14,15], and mental health [16]. Inversely, close others can experience altered and challenging relationships with the person with ABI [13], increased burden, and reduced quality of life [17,18]. Wider communities are also economically affected by the burden of informal care, including reduced workforce participation by caregivers [19].

The internationally recommended best practice approach to managing communication impairment after ABI is to provide communication partner training (CPT) to both the person with ABI and the people who communicate with them after ABI [20,21]. However, the substantial and growing burden of ABI makes it challenging to meet this recommendation at a global scale, for both health care systems generally [2,3] and the speech-language pathology profession specifically [22]. For example, in 2 recent national surveys of speech-language pathologists supporting people with ABI, a minority of 122 Australian speech-language pathologists self-reported using published, evidence-based programs to train familiar (11/110, 10%) and unfamiliar (10/75, 13%) communication partners of adults with stroke [22], with comparable proportions of 169 speech-language pathologists surveyed in the United Kingdom reportedly using published programs to train familiar (27/136, 19.9%) and unfamiliar (12/87, 14%) communication partners of adults with TBI [23].

Current research also indicates a need for neurorehabilitation professionals to support people with ABI in the effective use of social media [24], as communication difficulties can extend beyond face-to-face conversations to digital interactions. Potential neurorehabilitation support therefore extends to cyber safety training for people with ABI, with exploratory research indicating that more than half (n=54, 53.5%) of 101 surveyed Australian and New Zealand neurorehabilitation providers had clients who had been affected by cyberscams [25]. However, there is currently a lack of effective interventions supporting digital interactions through social media after ABI [25,26].

**Figure 1:**
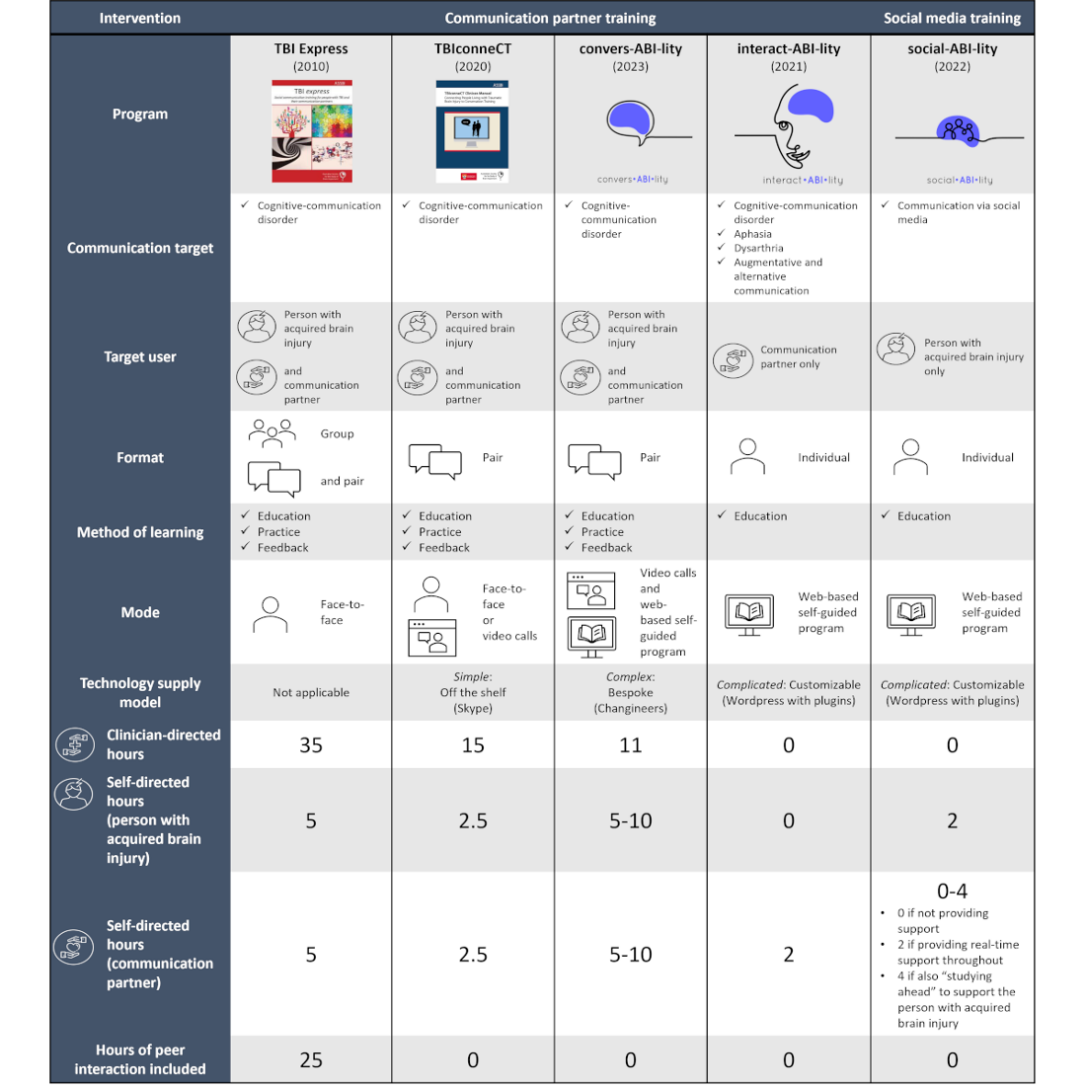
Comprehensive comparison of each tool in the Social Brain Toolkit with previously released communication partner training programs, "TBI Express" and "TBIconneCT". Program images and visual supports are included to improve accessibility. TBI Express and TBIconneCT cover images Copyright 2010, 2019, the Australasian Society for the Study of Brain Impairment [30,31], used with express permission of the copyright holder. Additionally, convers-ABI-lity, interact-ABI-lity, and social-ABI-lity logos Copyright 2020, author Melissa Miao for the Social Brain Toolkit.

To address these needs, the “Social Brain Toolkit” [27] was developed as a novel suite of 3 targeted communication interventions delivered via the internet. It includes a self-directed web-based training program, “social-ABI-lity,” specifically developed to address an identified need [24,26] to support people with ABI to use social media, and 2 stand-alone yet complementary CPT programs, “interact-ABI-lity” and “convers-ABI-lity.” interact-ABI-lity and convers-ABI-lity were derived from previously published, evidence-based [28,29], face-to-face and telehealth-delivered CPT programs targeting cognitive-communication disorders, “TBI Express” [30] and “TBIconneCT” [31]. A key differentiation from these previous programs is that convers-ABI-lity has a fully web-based service delivery model incorporating self-directed learning modules and weekly telehealth sessions between a person with ABI, communication partner, and speech-language pathologist. interact-ABI-lity is a short, self-directed learning program solely for the communication partner and features advice relating to aphasia (language impairment), dysarthria (weakness in speech muscles), augmentative and alternative communication (communication via methods other than natural speech, such as facial expression or a voice output device), and cognitive-communication difficulties (communication difficulties due to cognitive impairments). A comprehensive comparison of each intervention is presented in Figure 1.

Digital health interventions and applications targeting neurological conditions continue to proliferate with modern technologies [32,33]. However, the demonstration of their clinical effectiveness is insufficient to guarantee their successful implementation [34]. Digital health implementation can be highly complex [35], with growing concern in relation to complex challenges such as equity of access [36]. Despite a need for guidance, there is currently a paucity of empirical evidence to guide the process of tailoring implementation strategies [37-39]. This gap is compounded by misalignment between general implementation frameworks and the specific challenges of implementing digital health [40]. Therefore, there is currently an urgent need for guidance to inform the development of tailored, evidence-based implementation strategies that align with the burgeoning need for digital health.

It has been recommended that researchers consult stakeholders to identify implementation barriers and tailored strategies [37]. Therefore, this study aimed to coproduce this implementation knowledge with stakeholders [10] to support the deployment of implementation strategies: (1) at intervention launch, (2) iteratively during initial pilot (convers-ABI-lity) and 12-month implementation-effectiveness hybrid studies (interact-ABI-lity and social-ABI-lity) [41], and (3) in future versions. The Australian development team includes speech-language pathologist researchers LT, RR, and MB from the University of Sydney and EP and MM from the University of Technology Sydney, with advisory and steering committees comprising people with living experience of ABI and clinicians supporting them. Partner organizations include technology vendor Changineers, community partner Brain Injury Australia, and funding partner icare New South Wales. The coproduction of implementation knowledge was conceptualized and led by author MM, whose relevant experience and qualifications include a clinical background in speech-language pathology with specialized training in accessibility, prior experience of coauthorship with stakeholders, qualitative research and peer-review experience, and membership of a high-risk human research ethics committee.

Given the aforementioned medical and psychosocial complexity of ABI [1,3,11-16]; the complex nature of health care systems as a complex adaptive system (CAS) [42,43] facing the growing global burden of ABI [1-3]; and the complexity of implementation itself [44,45], specifically digital health implementation [35,46-49], researchers should anticipate that the implementation, scale-up, and sustainability of the Social Brain Toolkit might be highly complex. It has been suggested that the field of complexity science can offer essential insights to support health [50] and implementation [44] research contending with real-world complexity. However, given that researchers have used a wide range of conceptualizations of complexity [51], with interchangeable use of related terms and concepts [52-54], a clear definition of complexity was required for this study [51,54]. In this study, digital health implementation was conceptualized as a CAS in its own right, in which multiple “agents” (eg, implementation considerations and strategies) followed local rules that adapted to each other (eg, an implementation strategy reducing the impact of an implementation consideration). System “attractors” (eg, a new implementation strategy or consideration) could vary according to parameters such as the number, strength, and diversity of their connections, with some stability gained from the constraints of other agents but also lost as a result of the evolving, emergent nature of local rules. Despite conceptualizing implementation as a CAS, it must also be acknowledged that this study adopts the “least radical” conceptualization of complexity [51], in which complexity is conceptualized as varying between the “simple” (straightforward, predictable, and with few components), “complicated” (with multiple interacting components or issues), or “complex” (dynamic, unpredictable, and not easily disaggregated into constituent components) [55], rather than more radically viewed as something omnipresent at all times. This least radical conceptualization of complexity served as a logic pathway for the use of tailored implementation strategies [56] as recommended in implementation science, whereby implementation strategies could facilitate implementation by theoretically managing or reducing the complexity of implementation considerations from “complex” to merely “complicated” or “simple.”

### Aims

As described in the published protocol [10], the broad aim of this study was to support the implementation of the Social Brain Toolkit by coproducing implementation knowledge with people with ABI, close others, clinicians, and leaders in digital health implementation. The specific aims of the study were to (1) prioritize theoretically informed implementation targets for the Social Brain Toolkit; (2) understand the nature of these priorities; (3) coproduce targeted implementation strategies; and (4) explore potential interrelationships between implementation considerations and strategies as a CAS, as a post hoc aim formulated in response to emergent complexity within the data set.

## Methods

### Research Paradigm

This study was epistemologically, ontologically, methodologically, and axiologically oriented from a critical realist research paradigm [57]. Critical realism would (1) ontologically support the examination of underlying mechanisms beyond empirical observation; (2) enable an epistemologically pluralist stance in which researchers can value complexity science alongside implementation science and experiential knowledge alongside academic knowledge; (3) allow for pragmatic and pluralist methodologies, including both implementation science and complexity science approaches; and (4) axiologically support the wider emancipatory values of coproducing Open Science [58], implementing evidence-based practice, and improving health outcomes for people with ABI and their communities.

Therefore, this study was theoretically guided by the Nonadoption, Abandonment, Scale-up, Spread, and Sustainability (NASSS) framework [55]. The NASSS framework was selected for its comprehensive inclusion of 7 interacting domains of digital health implementation, scalability, and sustainability; systematic itemization of the degree of complexity in each of these 7 domains; specific focus on digital health; and alignment with author MM’s living experience of using technology to implement and scale speech-language pathology services.

### Study Participants

As shown in Table 1, participants were a purposive, maximum variation sample (N=35) of adults with living experience of ABI, communication partners, speech-language pathologists supporting people with ABI, and individuals with experience implementing digital health. Out of 10 individuals who had living experience of ABI, 2 (20%) also had either clinical experience of ABI or digital health implementation experience. In addition, 2 (40%) out of 5 clinicians and 1 (10%) out of the 10 people with ABI had prior experience with TBI Express [30] and TBIconneCT [31] and were thus able to directly compare their delivery and content with those of the potential or prototype versions of convers-ABI-lity and interact-ABI-lity (Figure 1).

People with living experience of ABI (10/35, 29%) were required to be discharged from hospital, participating at least 6 months after sustaining an ABI, and with adequate capacity to consent to study participation. An adapted consenting process [59], published as supplementary materials of the study protocol [10], was used screen capacity to consent. Participants with living experience of ABI were required to be based in Australia, where the Social Brain Toolkit was developed and where they could be financially reimbursed for their living experience expertise. People with ABI were recruited through tailored electronic flyers posted with alternative text on social media, as well as organizational websites, email distribution lists, and snowball recruitment.

Communication partners (11/35, 31%) were self-identified as individuals who interacted with a person with ABI at least once a week and who had not sustained an ABI themselves. Communication partners were also required to be based in Australia, where the Social Brain Toolkit was developed and where they could be financially reimbursed for their living experience expertise. They were also recruited through tailored electronic flyers, organizational websites, email distribution lists, and snowball recruitment.

Clinicians (5/35, 14%) were self-identified as qualified professionals practicing with a caseload of which at least 20% included people with ABI. No other restrictions were applied, including restriction on international participation. Clinicians were recruited through tailored electronic flyers, organizational websites, and email distribution lists.

Individuals experienced in digital health implementation (9/35, 26%) were required to have a research or industry track record in digital health implementation and were able to participate internationally. Eligible individuals were recruited via direct email to publicly listed contact details on university website profiles, researcher networks, and snowball recruitment.

Participant demographic information is described in detail in Table 1 and was previously published in the protocol [10].

**Table 1:** Participant demographic information, reported as an aggregate to preserve participant anonymity (N=35)^a^.

		Adults with experience of ABI^b^ (n=10, 29%)	Communication partners (n=11, 31%)	Clinicians (n=5, 14%)	Individuals with experience of digital health implementation (n=9, 26%)
Living experience		9 (90%) with living experience of ABI and 1 (10%) with living experience of both ABI and clinically supporting patients with ABI	11 (100%) with living experience of being a communication partner of someone with ABI	5 (100%) with clinical experience supporting people with ABI as speech-language pathologists	8 (89%) with living experience implementing digital health interventions for any condition and 1 (11%) with living experience of both ABI and digital health implementation
**Sex, n (%)**					
	Male	7 (70)	4 (36)	1 (20)	6 (67)
	Female	3 (30)	7 (64)	4 (80)	3 (33)
Location		Australia (inclusion criteria)	Australia (inclusion criteria)	1 (20%) from the United Kingdom, 1 (20%) from the Netherlands, and 3 (60%) from Australia	1 (11%) from Denmark and 8 (89%) from Australia
Education		1 (10%) with graduate diploma or certificate, 4 (40%) with bachelor’s degree, 4 (40%) with certificates or diplomas, and 1 (10%) with high school diploma	1 (9%) with a PhD^c^, 1 (9%) with a master’s degree, 2 (18%) with a graduate diploma or certificate, 3 (27%) with a bachelor’s degree, 3 (27%) with certificates or diplomas, and 1 (9%) with a high school diploma	2 (40%) with a master’s degree, 1 (20%) with a graduate diploma or certificate, and 2 (40%) with a bachelor’s degree	9 (100%) with a PhD
**Age (years), n (%)**					
	18-24	0 (0)	0 (0)	1 (20)	N/A^d^
	25-34	2 (20)	1 (9)	2 (40)	N/A
	35-44	4 (40)	2 (18)	1 (20)	N/A
	45-54	2 (20)	2 (18)	1 (20)	N/A
	55-64	1 (10)	1 (9)	0 (0)	N/A
	>65	1 (10)	5 (45)	0 (0)	N/A
**Time postinjury, n (%)**					
	>12 mo	9 (90)	N/A	N/A	N/A
	<12 mo	1 (10)	N/A	N/A	N/A
Clinical experience		N/A	N/A	3 (60%) working for >10 years with 80% to 100% of their caseload involving people with ABI, 1 (20%) working for <5 years with 80% to 100% of their caseload involving people with ABI, and 1 (20%) working for <5 years with 20% of their caseload involving people with ABI	N/A
Relationship with the person with ABI		N/A	3 (27%) friends, 3 (27%) spouses, and 5 (45%) family members	N/A	N/A

^a^This table is republished from the study protocol [10] under Creative Commons Attribution License BY 4.0.

^b^ABI: acquired brain injury.

^c^PhD: Doctor of Philosophy.

^d^N/A: not applicable.

### Study Design

#### Overview

This study includes a (1) mixed methods coproduction of implementation knowledge with (2) collaborative autoethnography of the coproduction process and (3) post hoc analysis of the complexity of implementation. The full methodological details and original materials used in the coproduction were previously published in an Open Access study protocol [10]. An additional step in the analysis and the methodological details of collaborative autoethnography and network analysis are also described below.

#### Coproduction of Implementation Knowledge

The coproduction of implementation knowledge was completed as described in the published protocol [10], using an explanatory sequential mixed methods design [60] as follows:

1. Author MM used the NASSS Complexity Assessment Tool [61] to script and edit a series of videos explaining each domain of the NASSS framework [55] in relation to the Social Brain Toolkit. The videos were short, slowly paced, captioned in plain English, and included both visual and auditory accessibility. Downloadable video files were published Open Access as supplementary materials of the study protocol [10].

2. Videos were embedded with large sans serif transcripts within an electronic prioritization survey created using Qualtrics software (Qualtrics International Inc) [62]. To identify the 4 most highly ranked domains, respondents rated, commented on, and ranked the perceived significance of each domain of the NASSS framework for the implementation of the Social Brain Toolkit. The survey design, layout, and questions, refined through 3 rounds of piloting from researcher, caregiver, and clinician perspectives, are downloadable as supplementary materials of the Open Access study protocol [10]. People with expert living experience of ABI (10/35, 29%) and people with digital health implementation experience (9/35, 26%) accessed the videos and questions via screenshare during a 1- to 2.5-hour video call interview with a speech-language pathologist researcher (MM), whereas clinicians (5/35, 14%) and communication partners (11/35, 31%) completed the electronic survey independently in their own time. Leaders in digital health implementation (9/35, 26%) viewed the video transcripts rather than the accessible videos. People with living experience expertise in ABI (10/35, 29%) viewed and member checked each of their responses in real time via screenshare on Teams (Microsoft Corporation) [63] before proceeding to the next question.

3. Over a series of 7 focus groups containing a diversity of 3 to 6 people with living experience of ABI, close others, or clinicians, the 4 most highly ranked domains of the NASSS framework were verbally discussed [55]. These were the (1) condition of ABI, (2) technology used to deliver the interventions in the Social Brain Toolkit, (3) supply- and demand-side value propositions of the Social Brain Toolkit, and (4) adopters of the Social Brain Toolkit. Qualitative survey and interview data pertaining to these 4 domains were anonymously and visually presented via a screen-shared slideshow summary, in combination with preliminary systematic review findings [41], followed by 1 to 3 prompt questions for group discussion. Plain English questions are available in the study protocol, with a detailed outline of time allocations and procedures provided in supplementary materials [10]. In addition, participant-generated discussion points were sequentially incorporated over the span of the 7 focus group discussions to promote dialogue across all participants. Each 3-hour focus group was facilitated using Teams (Microsoft Corporation) [63] video calls, with at least 1 qualified speech-language pathologist researcher present in each group. Interviews and focus groups were audio recorded and video recorded for verbatim transcription. Data were collected from April 13, 2021, to November 18, 2021.

4. Data were deductively analyzed [64] using the NASSS framework [55]. Meaning was parsed and condensed from the verbatim transcripts in Excel (Microsoft Corporation) [65]. Domains and subdomains were then coded with reference to the original published domain and subdomain definitions [55] which had been reprinted as a codebook in large, sans serif font with color codes. As described in the protocol [10], only the top 4 domains were included in the final narrative report.

An additional step not specified in the protocol was a deductive content analysis [64] of the implementation considerations and strategies within each domain and subdomain from the verbatim transcripts. Author MM also deductively identified the key implementer (ie, clinician, developer, or both) of each strategy.

#### Collaborative Autoethnography of the Research

Collaborative autoethnography may help implementation scientists reflexively document and learn from experiences of intersectionality during research coproduction [66] and “shed light on the challenges, solutions and processes of producing or co-producing knowledge” [66]. Collaborative autoethnography was also used to qualitatively evaluate the prioritization process and outcomes through stakeholder reflection. All participants with living experience of ABI, both those with direct living experience of ABI (10/35, 29%) and those with indirect living experience of ABI as a close other (11/35, 31%) or clinician (5/35, 14%), were eligible to participate in the collaborative autoethnography. Of the 26 eligible participants, 9 (35%) consented to be coauthors, including a close other (BT) who participated in the authorship of the protocol [10] and a close other (MTM) who participated in the authorship of the results. An additional person with living experience expertise in ABI (CR) was invited to participate in the authorship of the study protocol at the suggestion of a study participant. The methodology followed that outlined by Ratnapalan and Haldane [66], in which (1) the primary author (MM) solicited first-person narratives from the coauthors through their preferred communication mode, (2) the coauthors refined and approved their contributions, and (3) all authors approved the final narrative. A notable deviation from the published method was that all authors were given the opportunity to have their narrative contributions attributed to them by name, if desired.

#### Complexity of Implementation

##### Relational Data

Initially, the documentation of potential interrelationships between the identified implementation considerations and strategies was necessary during primary analysis to avoid duplicate findings and to ensure that only unique, mutually exclusive considerations and strategies were identified and counted (ie, strategies 1.3, 1.6, and 2.5 repeat but are not recounted toward the total). However, relationships were ultimately formally and systematically documented across the entire qualitative data set (Multimedia Appendix 1) [67], as participants both explicitly and implicitly described multiple additional interrelationships. These included strategies that targeted multiple considerations, considerations that interacted with each other, and strategies that may benefit other strategies.

**Figure 2:**
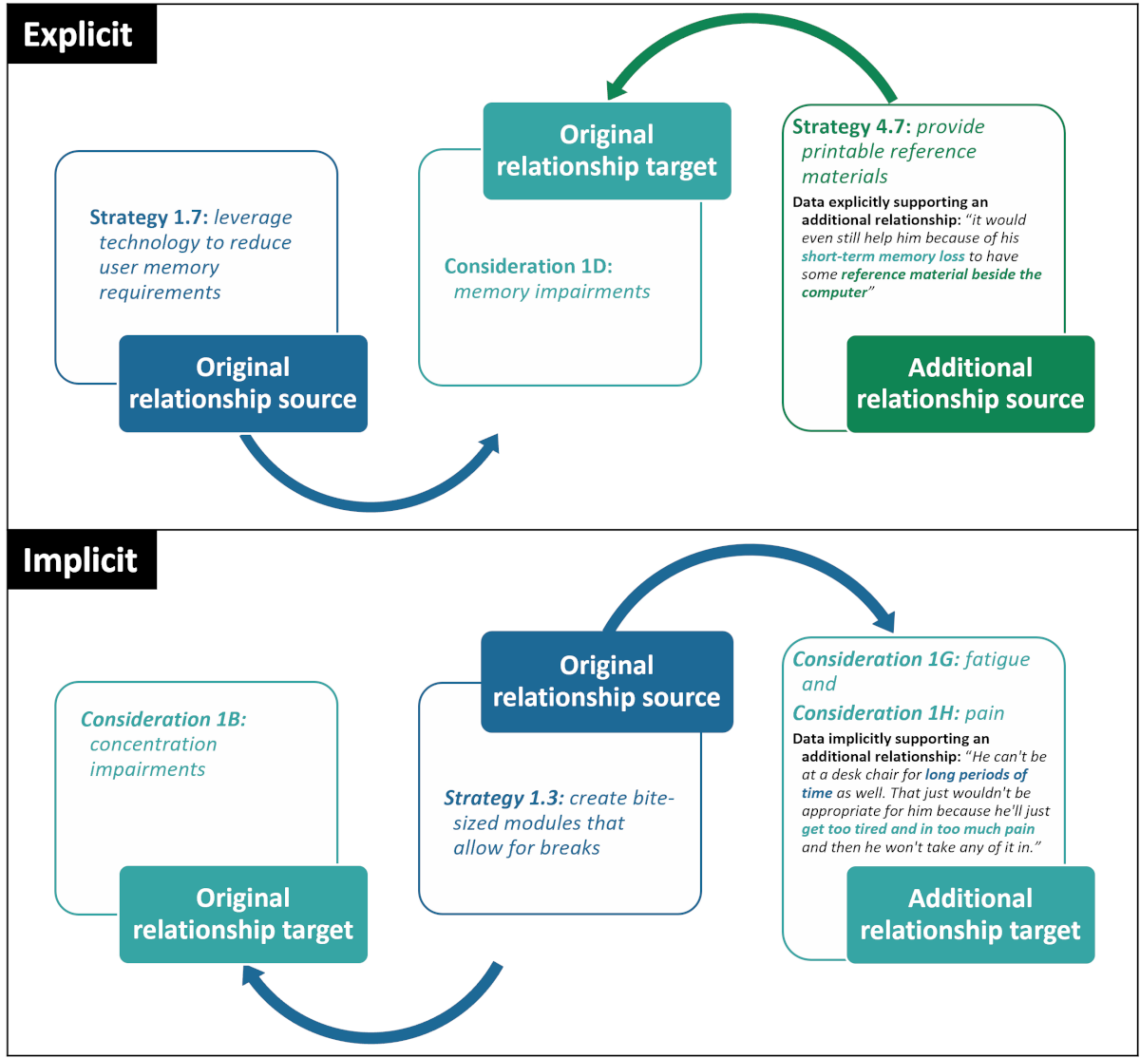
Examples of how additional implicit and explicit relationships (between right and center boxes) were identified during post hoc network analysis beyond the original targeted relationships reported in Table 2 (between left and center boxes). As indicated by arrow direction, additional relationships can occur between any strategy or consideration, in either direction.

Interrelationships were identified through either explicit or implicit reference in the verbatim qualitative focus group or interview data; examples are provided in Figure 2. For example, although suggestions to “leverage technology to reduce user memory requirements” (strategy 1.7) originally targeted “memory impairments” (consideration 1D), an explicit reference to “short term memory loss” in the stakeholder suggestion to “provide printable reference materials” (strategy 4.7) explicitly identified print materials as also potentially benefiting individuals with memory impairments. As an example of an implicit relationship, although the strategy to “create bite-sized modules that allow for breaks” (strategy 1.3) was identified as supporting individuals with “concentration impairments” (consideration 1B), it can be inferred that short modules could also support someone with “fatigue” (consideration 1G) and “pain” (consideration 1H) because of the statement that these comorbidities make it difficult to sit for “long periods of time.”

##### Visual Network Analysis

Following the identification of these relationships, author MM completed a network analysis [68]. Specifically, the visual network analysis (VNA) method was selected to provide a thick description and comprehensive visualization of qualitative relational data [69]. Reflecting the study’s underlying conceptual and theoretical focus on complexity, VNA was used to examine the mechanisms that emerge from a network’s composition [70]. VNA was thus selected to understand and explore the relational distribution of the implementation of the Social Brain Toolkit without having to invoke holistic or individualistic explanations [69,70]. It was also selected to enable presentation, rather than representation, of how the Toolkit’s implementation is constantly affected by relationships in a CAS. VNA was also able to accommodate a pragmatic, a priori cutoff of the network at domains 1 to 4 [69].

Although VNA cannot be proceduralized into neatly delineated substeps [71], it can be broadly outlined as progressing from qualitative data collection and coding to visualization and narrative analysis and interpretation [69]. Visualization was completed in Gephi (Gephi Consortium) [72] software. In addition to its ability to represent each consideration and strategy as a circular “node” and each directional relationship as a curved “edge,” Gephi was selected for its capacity to visually emphasize particular network properties through proportionality [69]. That is, the size of each node could reflect its total outgoing relationships, with larger nodes indicating a larger number of outgoing relationships, and vice versa. Its ForceAtlas algorithm was selected as adequate to account for node sizes in a layout of 100 nodes. Each domain was indicated by 1 of 4 colors, with considerations and strategies indicated by a lighter and darker shade of that color, respectively. Edges were colored according to their source node. Narrative analysis and interpretation focused on the number of outgoing connections from each node to semi-quantitatively identify the potentially most impactful “leverage points” within the identified networks.

### Rigor

#### Coproduction of Implementation Knowledge

As noted in the *Study Design* section of the same name, interviewees member checked the transcription and interpretation of their survey and interview responses in real time via screen-shared video calls. All interview and focus group transcriptions were also verified against the original audio and video recordings. All participants were provided with the opportunity to further member check the interpretation of their qualitative data, with any changes or clarifications incorporated and reported as original data. Of the 35 participants, 15 (43%; including 7/10, 70% of people with ABI; 1/11, 9% of close others; 4/5, 80% of speech-language pathologists; and 3/9, 33% of leaders in digital health implementation) confirmed the interpretation of their data, with either no changes or minor clarifications made. Furthermore, coding for 5 (71%) of the 7 focus groups was verified by 1 or 2 authors who directly participated in those focus groups (RM, AS, BM, J-CE, MRW, MTM, MW, or LW), and 25% of the coding of the first individual interview was verified by a second author (DD). In addition, direct stakeholders in the implementation research are coauthors who have contributed to the analysis, interpretation, and write-up of the results.

#### Collaborative Autoethnography of the Research

The following three key criteria for robust collaborative autoethnography [66] were adhered to: (1) firsthand accounts of the implementation research process from all the coauthors were included across the 2 publications of the study protocol and results, (2) the accounts focused on research challenges and experiences rather than retelling the study results, and (3) the accounts included broader reflections on Open Science [58] and the systems and culture of academia [10]. Finally, both the research protocol and results were comprehensively reported according to the reporting guideline for priority setting of health research [73] to address aspects pertinent to coproduction more comprehensively than required by standard mixed methods guidelines.

#### Complexity of Implementation

As complexity science approaches follow different and often contrasting standards of rigor from those followed in traditional health research [50], the rigor of the network analysis depended on uniformity throughout the study in the theoretical mapping of relationships [69] rather than the positivist “triangulation” of data between multiple researchers. Therefore, network analysis was completed by the author who was most familiar with the theoretical framework and immersed across the full data set (MM).

### Ethical Considerations

The study received human research ethics approval with commendation from the University of Technology Sydney (ETH20-5466).

All participants provided informed written consent. In recognition of their living experience expertise, people with ABI and their close others were reimbursed at the rate stipulated by Health Consumers New South Wales [74]. All data were stored and transferred securely. Identifying audio and video data were deidentified as much as possible and stored separately from identifying information. Collaborative autoethnographic data were attributed to authors by name only when discussed and agreed upon in writing during the authorship process.

## Results

### Main Results

Across the top four prioritized domains of (1) condition, (2) technology, (3) value proposition, and (4) adopters of the Social Brain Toolkit, the coproduction of implementation knowledge yielded a total of 48 unique digital health implementation considerations and 52 unique, tailored implementation strategies for both developers and clinicians. Collaborative autoethnography identified both the benefits and challenges of coproducing implementation research within traditional academic systems. Post hoc network analysis yielded 172 unique relationships between the 100 implementation considerations and strategies and identified the potentially most impactful implementation strategies within the network.

### Coproduction of Implementation Knowledge

#### First Priority Domain: Condition

Stakeholders coproduced a total of 14 implementation considerations and 17 tailored strategies within their top priority domain of the “condition” of ABI (Table 2). These spanned all 3 subdomains, including the nature of the condition, its comorbidities, and sociocultural factors.

**Table 2:** Overview of the 14 stakeholder-identified digital health implementation considerations (1A-1N) and 17 tailored strategies (1.1-1.17) in the first priority domain: “condition.”

Subdomain and consideration			Strategy tailored to the consideration		Implementer
**Nature of ABI^a^**					
	1A: stage of recovery	1.1: disseminate the Social Brain Toolkit to communication partners while the person with ABI is in the acute stage of recovery, and to people with ABI between discharge and rehabilitation 1.2: suggest that speech-language pathologists can help determine whether a tool would be suitable for a person with ABI and their communication partners		Clinician and developer	
	1B: concentration impairments	1.3: create bite-sized modules that allow for breaks 1.4: provide simple, clear, and consistent user interfaces		Developer	
	1C: communication impairments	1.5: use simple language 1.6: ensure compatibility with alternative technology access methods (eg, eye gaze, voice, or touchscreen)		Developer	
	1D: memory impairments	1.7: leverage technology to reduce user memory requirements (eg, automation, repetition, or guided structure)		Developer	
	1E: impairments in emotional regulation	1.8: ensure fast and easy usability		Developer	
	1F: self-esteem	1.9: ensure achievable task difficulty 1.10: maintain a supportive tone and language 1.11: avoid conveying judgment or penalty 1.12: empower people with ABI with the option to provide feedback about the tools		Developer	
**Comorbidities**					
	1G: fatigue 1H: pain	1.13: suggest completion in the morning		Clinician and developer	
	1G: fatigue 1H: pain	See also 1.3: create bite-sized modules that allow for breaks		Developer	
	1I: physical impairments 1J: sensory impairments	1.14: avoid placing time limits on tasks 1.15: ensure multimodal accessibility when designing tasks (eg, avoid designing tasks that can only be completed visually) See also 1.6: ensure compatibility with alternative technology access methods		Developer	
**Sociocultural factors**					
	1K: nature and availability of social support	1.16: suggest seeking multidisciplinary support beyond speech-language pathology if needed		Clinician and developer	
	1L: invisibility and social stigma of disability 1M: socioeconomic situation 1N: gender differences in health care access	1.17: ensure tools are available on mainstream device types		Developer	

^a^ABI: acquired brain injury.

Although developers could deploy all 17 strategies in this domain by making various design, accessibility, and language adjustments, speech-language pathologists could also deliver 4 of these strategies within clinical settings. First, when considering the subdomain concerning of the nature of a person’s ABI, stakeholders highlighted the importance of the “stage of recovery” of a person with ABI (consideration 1A). Communication partners expressed a desire to receive the Social Brain Toolkit “as early as possible” (Communication partner 6, focus group 6), whereas people with ABI may not yet be ready: “I was just focusing on getting through another day, so giving me any work or anything, I just know in hospital it was beyond me at that time” (Person with living experience of ABI 2, focus group 3). Therefore, stakeholders suggested disseminating the Social Brain Toolkit to close others while the person with ABI is in the acute stage of their recovery, and to people with ABI between discharge and rehabilitation (strategy 1.1). Stakeholders also expressed a desire for speech-language pathologists to help them determine whether a tool would be suitable for a person with ABI and their communication partners (strategy 1.2). This approach was perhaps best summarized in the words of a person with ABI who reflected on the best time for dissemination as follows:

Probably not straight after I’d had the accident [for me], but my parents, my friends, my best friend, who came and saw me every day at the rehab[ilitation] center, every day at the hospital, he could have done this straight up, so the support that came from it, he’d have been able to tell my parents, and my parents would be able to tell my sisters, and my sisters would be able to effectively understand what’s going on with me.

Person with living experience of ABI 3, focus group 2

In the theoretical subdomain concerning comorbidities, speech-language pathologists were identified as potentially being able to assist the management of “fatigue” (consideration 1G) and “pain” (consideration 1H) by suggesting that people with ABI complete the intervention in the morning (strategy 1.13). Finally, in the subdomain concerning sociocultural factors, speech-language pathologists were identified as being able to seek multidisciplinary support if needed (strategy 1.16):

When speech pathologists are using these types of tools with clients, especially when you’re working on communication with a conversation partner, it can sort of reveal sort of pre-injury relationship or marital or friendship issues. And sometimes I find I need to get people to see a social worker, psychologist, relationship counsellor, before we then can address communication, because I feel like sometimes that’s just worth as a clinician, being mindful of.

Speech-language pathologist 1, focus group 6

#### Second Priority Domain: Technology

Stakeholders ranked the “technology” used to deliver the Social Brain Toolkit interventions as the second most important implementation target. They identified a total of 14 considerations and 8 unique tailored strategies across all 4 subdomains, including the key features of the technology, the knowledge made visible by and required to use it, and supply models (Table 3).

Technological considerations could primarily be addressed by developers, including ensuring a responsive web design across all device types (strategy 2.1), providing appropriate software training and video guides (strategies 2.4-2.7), and creating clinician dashboards (strategy 2.3). However, speech-language pathologists could also deliver 2 strategies. First, assistance with hardware setup (strategy 2.5) could only be provided to people with ABI who might want “someone to help set up the computer” (Person with living experience of ABI 5, focus group 3) by a clinician as opposed to a developer. Second, clinicians could reinforce the recommendation that telehealth video calls be conducted via a tablet or computer (strategy 2.2) to make nonverbal communication visible:

...observations about nonverbal communications, so how the couple are sitting, whether they’re facing each other, looking at gestures and things like that. Often with smartphones you can see the person’s face but you’re not getting much else apart from that. So, from a clinician’s perspective, all of that other non-verbal stuff is really important and good to observe, which you don’t always get with the smartphone [with] just people often holding it up.

Speech-language pathologist 1, focus group 6

**Table 3:** Overview of the 14 stakeholder-identified digital health implementation considerations (2A-2N) and 8 tailored strategies (2.1-2.8) in the second priority domain: “technology.”

Subdomain and consideration		Strategy tailored to the consideration	Implementer
**Key features of the technology**			
	2A: screen size 2B: camera 2C: accessibility 2D: portability 2E: stability 2F: affordability 2G: reliability	2.1: ensure responsive web design	Developer
**Knowledge made visible by the technology**			
	2H: interaction with clinicians 2I: interaction between communication partners	2.2: recommend conducting telehealth video calls via tablet or computer	Clinician and developer
	2J: patient-reported measures 2K: progress data	2.3: provide a clinician dashboard See also 1.6: ensure compatibility with alternative technology access methods	Developer
**Knowledge required to use the technology**			
	2L: digital literacy 2M: unfamiliar processes	2.4: provide an introductory software tutorial for clinicians	Developer
	2L: digital literacy 2M: unfamiliar processes	2.5: provide hardware setup support for people with ABI^a^	Clinician
	2L: digital literacy 2M: unfamiliar processes	2.6: provide an introductory software tutorial for people with ABI and their communication partners 2.7: provide video guides for people with ABI and their communication partners	Developer
**Supply model**			
	2N: complexity of technological requirements	2.8: tailor supply models to the technological complexity of each tool	Developer

^a^ABI: acquired brain injury.

#### Third Priority Domain: Value Proposition

A total of 8 considerations and 13 tailored strategies were identified in relation to the “value proposition” of the Social Brain Toolkit. These spanned both the supply- and demand-side value subdomains (Table 4).

On the demand side, developers of the Social Brain Toolkit could direct efforts toward communicating the Toolkit’s value in improving communication after ABI (consideration 3A), supporting communication partners after ABI (consideration 3B), addressing social media use after ABI (3C), and improving access to support (consideration 3D). On the supply side, developers could offer improvements to service efficiency (consideration 3E) and the provision of person-centered care (consideration 3F). Developers could also address the financial sustainability of the interventions (consideration 3G) by adopting a hierarchical pricing structure, with least to no payment from people with ABI, and the most funding from organizations, government, or insurance (strategy 3.12). It was also recommended that developers address the scalability (consideration 3H) of the Social Brain Toolkit in a strategic, stepwise manner (strategy 3.13).

**Table 4:** Overview of the 8 (3A-3F) stakeholder-identified digital health implementation considerations and 13 tailored strategies (3.1-3.13) in the third priority domain: “value proposition.”

Subdomain and consideration			Strategy tailored to the consideration		Implementer
**Demand-side value**					
	3A: improving communication after ABI^a^	3.1: generate and update evidence of benefit 3.2: provide video stories from people with ABI		Developer	
	3A: improving communication after ABI	3.3: suggest that speech-language pathologists can refer people with ABI and their communication partners to the tools 3.4: gain momentum through clinician champions		Clinician and developer	
	3B: supporting communication partners after ABI	3.5: ensure that communication partner training is described in an empowering rather than judgmental manner		Developer	
	3C: addressing social media use after ABI	3.6: create clear, simple messages to explain and differentiate between the 3 different tools in the Social Brain Toolkit		Developer	
	3D: improving access to support	3.7: disseminate the Social Brain Toolkit through as many channels as possible		Developer	
**Supply-side value**					
	3E: improving service efficiency 3F: providing person-centered care	3.8: use the language of the funder to communicate the Social Brain Toolkit’s benefits 3.9: communicate that the Social Brain Toolkit complements or adds value to existing services, rather than replacing them 3.10: demonstrate to funders that there is a financial benefit to providing the tools 3.11: maximize user autonomy		Developer	
	3G: sustainability	3.12: adopt a hierarchical pricing structure with least to no payment from people with ABI and most from organizations, government, or insurance		Developer	
	3H: scalability	3.13: adopt a strategic, stepwise approach to scale-up		Developer	

^a^ABI: acquired brain injury.

There were 2 ways in which clinicians could also be instrumental in the dissemination of the Social Brain Toolkit. First, although stakeholders emphasized the importance of disseminating the Social Brain Toolkit through as many channels as possible, including through professional groups and organizations, social media, and a website, clinicians were the preferred referral source for people with ABI and their communication partners (strategy 3.3): “I would take their word because they are professionals, and the advice directly from them is better than Google or the Internet” (Communication partner 7, focus group 2). Second, leaders in digital health implementation highlighted the value of the Social Brain Toolkit gaining momentum through clinician champions (strategy 3.4):

It’s really critical from your aspect that you find some of those people—and there’s always a few of them, so there will always be a small percentage of people that look at it and go ‘absolutely, this is a great idea, let’s do it.’ Then the more it gets out there, the more people will start to hear about it and become onboard with it.

Digital health implementation leader 8

Although people with ABI, communication partners, and clinicians shared similar views on the demand-side value proposition of the Social Brain Toolkit, including common desires for “improving communication after ABI” (consideration 3A), “supporting communication partners after ABI” (consideration 3B) and “improving access to support” (consideration 3D), implementation strategies could be tailored to each stakeholder type, as outlined in Textbox 1. Some demand-side strategies might also apply to the supply-side proposition for health care systems. Maximizing user autonomy (strategy 3.11) as a strategy to support the supply-side value of providing person-centered care could offer demand-side value for users. Likewise, generating and updating supporting evidence (strategy 3.1) could be important for both supply- and demand-side value propositions.

**Tailoring strategies to specific stakeholder groups to address the value proposition domain.**

**People with acquired brain injury (ABI)**

- 3.2: provide video stories from people with ABI
- 3.3: suggest that speech-language pathologists can refer people with ABI and their communication partners to the tools
- 3.11: maximize user autonomy

**Close others**

- See also 3.3: suggest that speech-language pathologists can refer people with ABI and their communication partners to the tools
- 3.5: ensure that communication partner training is described in an empowering rather than judgmental manner
- 3.7: disseminate the Social Brain Toolkit through as many channels as possible

**Clinicians**

- 3.4: gain momentum through clinician champions
- 3.6: create clear, simple messages to explain and differentiate between the 3 different tools in the Social Brain Toolkit

**Health care systems**

- 3.1: generate and update evidence of benefit
- 3.8: use the language of the funder to communicate the Social Brain Toolkit’s benefits
- 3.9: communicate that the Social Brain Toolkit complements or adds value to existing services, rather than replacing them
- 3.10: demonstrate to funders that there is a financial benefit to providing the tools

#### Fourth Priority Domain: Adopters

A total of 12 considerations and 14 strategies were identified in relation to the fourth priority: the “adopters” of the Social Brain Toolkit. These included people with ABI, their close others, and clinicians (Table 5).

Although stakeholders identified ways in which developers could provide support across all 3 subdomains, clinicians could also support implementation for people with ABI and their close others. For close others, this included facilitating buddy or peer support (strategy 4.10) and providing reminders (strategy 4.11). For people with ABI, this included providing the setup checklist created by developers (strategy 4.2), reminders to complete the intervention at a regularly scheduled time (strategy 4.4), positive feedback of progress and achievement (strategy 4.5), upfront communication of the estimated time requirement (strategy 4.6), and printable reference materials (strategy 4.7). It also included the aforementioned strategy in the technology domain of providing hardware setup support for people with ABI (strategy 2.5).

In addition, it was identified that people with ABI may benefit from being accountable to a clinician (strategy 4.8):

From our experience, the other big thing that helped: [spouse] got a lot of homework when she did speech [therapy] and that would always be done...At least by the night before. Yeah, because she didn’t want to go to her speech therapist not having done the homework.

Communication partner 2, focus group 3

Similarly, people with ABI may enjoy being accountable to their peers with ABI (strategy 4.9):

Working in groups works for me. Like if [peer with living experience of ABI] and I were working as a team, right, and we were meeting at say 12 o’clock every Tuesday and we had a certain deliverable that we had to meet, then he could motivate me to get my bit done and I can motivate him. Working as a team is often a good motivator for me. [It would be ideal if] at the end of the completion of the course we’d all go out and have a big cup of coffee together or something like that.

Person with living experience of ABI 4, focus group 5

Other people with ABI could also be a mentor:

I’m wondering if it would be useful when someone had gone through the course and had already graduated, to become a partner with someone who you are introducing it to? Given that people had the skill, or they got on [with each other], because I think, then you’ve got someone being mentored by a graduate.

Communication partner 1, focus group 2

Full descriptions of all 100 considerations and strategies are provided in Multimedia Appendix 1.

**Table 5:** Overview of the 12 stakeholder-identified digital health implementation considerations (4A-4L) and 14 tailored strategies (4.1-4.14) in the fourth priority domain: “adopters.”

Subdomain and consideration			Strategy tailored to the consideration		Implementer
**All**					
	4A: user experience	4.1: conduct user or persona testing		Developer	
**People with ABI^a^**					
	4B: access to a viable setup	4.2: provide a setup checklist See also 2.5: provide hardware setup support for people with ABI		Clinician and developer	
	4C: expectation to return and reorient to an intervention	4.3: streamline the number of steps		Developer	
	4C: expectation to return and reorient to an intervention	4.4: recommend and provide reminders to complete the intervention at a regularly scheduled time 4.5: provide positive feedback of progress and achievement 4.6: provide upfront communication of the required time commitment 4.7: provide printable reference materials 4.8: facilitate accountability to a clinician 4.9: facilitate accountability to peers or mentors with ABI		Clinician and developer	
**Close others**					
	4D: performance anxiety 4E: familiarity with content 4F: expectation to provide social support 4G: expectation to provide technological support 4H: expectation to participate in interventions	4.10: facilitate buddy or peer support for close others 4.11: provide reminders for close others		Clinician and developer	
**Clinicians**					
	4I: responsibility to support and follow up the use of the tools 4J: need to adjust from face-to-face delivery	4.12: provide a checklist for web-based service delivery		Developer	
	4K: need to provide technological support to people with ABI and close others	4.13: provide troubleshooting education and support for clinicians		Developer	
	4L: area of clinical practice	4.14: provide clinical professional development in the program		Developer	

^a^ABI: acquired brain injury.

### Collaborative Autoethnography of the Research

The collaborative autoethnography included reflections on the methods of the study from the perspective of the coauthors with living experience of ABI, close others, and clinicians. Reflections on the methods of the study were previously published in the study protocol [10], with academic writing interleaved with quotations from people with living experience to communicate the equal value of both academic and experiential knowledge. The following results continue this reflection from the final stages of the research.

Upon appraising the outcomes of this implementation research, author RM, who has living experience of ABI, shared the following reflection:

This implementation study has not come from professionals working on the basis of what they believe will work best for their patients, this comes from those living with experience of the condition having input into what treatments would have helped them and when those treatments should have been available. This is a rare example of supply meeting demand.

Likewise, author J-CE provided the following conclusion as both a clinician and person with living experience of ABI:

This Toolkit is going to be an invaluable resource for both patients and clinicians in the future.

Stakeholders reported experiencing personal satisfaction in coproducing new knowledge, with author RM adding the following:

Not only is a very great need being met with this research study, but it also represents the intersection of my professional life as an editor and my personal life as someone living with a brain injury. I’m more than thrilled—I’m over the moon that my experiences are being recognized academically. I can’t be more proud to be a coauthor of this study—it’s beyond fulfilling, it’s wonderful as well.

Author J-CE, who has both living and clinical experiences of ABI, shared a similar reflection:

Participating in and coauthoring this research was very interesting; being able to share both my living experience with my ABI and my experience caring for people with an ABI was extremely rewarding and validating. I got to learn a lot from everyone involved in this research, especially the different types of support people with an ABI received across different states in Australia.

Sharing the living experience of ABI in the writing stage was identified to be an emotional experience. From their living experience as a close other of a person with ABI, author MTM notes that their reflection was “a very difficult passage to write, with so many memories and feelings.”

Reflecting from the researcher perspective of facilitating coproduction, MM offers the following four key learnings for fellow researchers wishing to engage in coproduction:

1. *I found that coproduction required continual courage and perseverance, as it requires a researcher to overcome the inherently elitist traditions of academia, procedural inertia where there is a lack of precedent, and personal vulnerability in doing the unconventional. I particularly experienced this when interleaving first-person accounts of living experience with traditional academic writing, trying to secure and provide payment for living experience expertise, and asking for this expertise to be published as equivalent to an academic degree. I found it helpful to find solidarity at conferences with other researchers who were coproducing their research, and to view setbacks within our collective, longer-term aim to normalize the democratization of knowledge. I also found co-authors with living and clinical experience were a constant touchstone and encouragement during the isolation and struggle of driving implementation efforts [45], and was greatly encouraged to find allyship in journal editors who were committed to inclusive and innovative publishing practices which recognize living experience expertise.* Production Editor Hannah Reinhardt communicated: *I agree with you strongly that the expertise of those with living experience should be acknowledged in the research community, and this is a cause that is of personal importance to me. I was therefore thrilled to have the opportunity to raise this with JMIR's management team, in hopes of expanding our affiliation regulations. I am fortunate enough to work with an excellent group of people who are always happy to take the time to review our processes and work on innovative solutions, so this is very much a shared achievement.*

2. *As CR expressed in the study protocol [10], coproduction is a deeply human, relational process, and it is only in the context of these relationships that its ethically and politically complex nature [75] can be managed. Insofar as it can be known in advance, clearly communicating expectations upfront, such using accepted criteria for authorship [76] to determine authorship expectations, was helpful to plan and achieve authentic collaboration. However, coproduction often contravenes a research tradition of obtaining a clearly replicable scientific procedure. I found it important to empower coauthors to make informed choices within its complexities. For example, although the collaborative autoethnographic method [66] prescribes anonymization of co-author contributions in the interests of ‘confidentiality,’ author decisions and indeed enthusiasm to be identified by name, with informed discussion of the potential personal and professional risks of doing so, was potentially a less paternalistic approach than anonymizing all coauthors like participants. An additional example was the need for a trusting relationship in which to raise unforeseen challenges, such as receiving predatory conference and journal emails.*

3. *As my clinical background was not in ABI, my coauthors provided invaluable expertise in ABI that far exceeded what I could read in academic literature and gave me an invaluable sense of purpose in my work. Coproduction also transformed my perspective of the research process, particularly the many places patient and community voices are missing from it. As ABI does not discriminate, the final author lists are a unique collective of people from all walks of life, including film and television, professional sport, health, and education. As RM and J-CE mentioned, each person brought a wealth of personal and professional experience in addition to their living experience of ABI, and this diversity of knowledge and experience was an asset to addressing the complexity of implementation. However, as shown by a markedly lower response rate in member-checking and the ‘division of labor’ across publications for communication partner co-authors, close others stood out to me as potentially requiring more proactive inclusion and financial support to avoid their underrepresentation or absence from research.*

4. *Researchers may benefit from allocating additional time when planning and scoping coproduced projects, as a key challenge I observed was the incongruity between the natural timeline of relationship and the tight deadlines of academic research.*

### Complexity of Implementation

**Figure 3:**
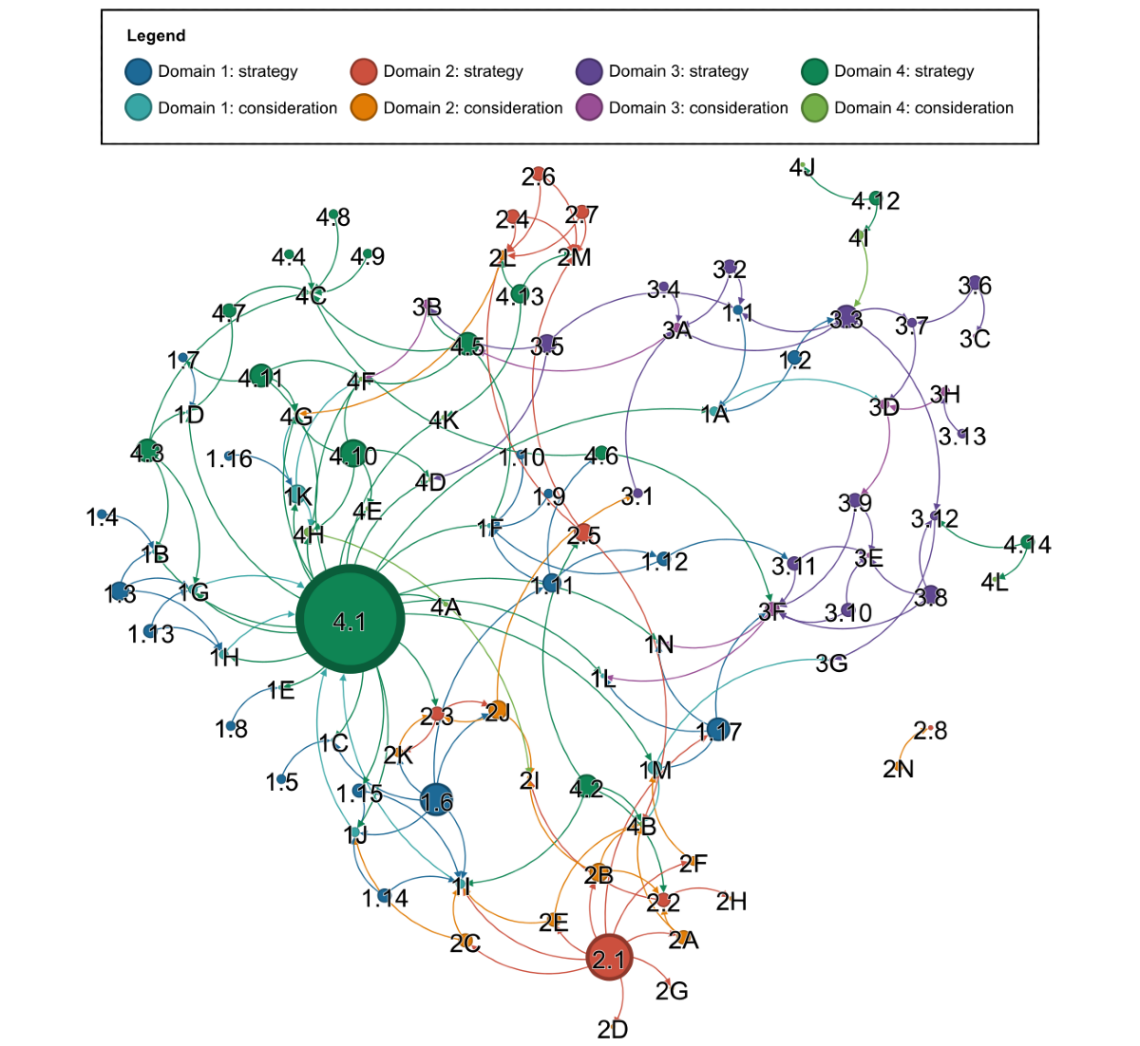
Network visualization of 172 unique, directional relationships between the 100 implementation considerations and strategies identified in the 4 prioritized domains. Each consideration and strategy is indicated as a “node,” represented by a circle, with each directional relationship shown as an “edge,” indicated by a curved arrow. The size of each node reflects its total outgoing relationships, with larger nodes indicating a larger number of outgoing relationships, and vice versa.

The post hoc network analysis yielded a total of 172 unique relationships between the 100 implementation considerations and strategies (Figure 3). The VNA provided an explicit visualization of the complexity reported by leaders in digital health implementation, one of whom remarked that the implementation domains were “all going back to the same problem that I said before...Essentially, I don’t think your questions...are all independent; I think they’re all interrelated” (Digital health implementation leader 7).

As another leader in digital health implementation acknowledged, implementation findings were revealed to be interdependent across all domains and subdomains: “I don’t mean to scare you, but there’s layers and layers of complexity” (Digital health implementation leader 1). All considerations and strategies were revealed to be connected in a network, except for a singular external connection between the “complexity of technological requirements” (consideration 2N) and a targeted strategy to “tailor supply models to the technological complexity of each tool” (strategy 2.8).

By contrast, strategy 4.1, to “conduct user or persona testing,” represented the highest number (22) of outgoing connections. As 64% (14/22) of these connections targeted the considerations in the stakeholders’ top priority domain of the “condition” of ABI (considerations 1A-1N), this strategy represented both high impact and high stakeholder priority. With fewer than half (9/22, 41%) as many outgoing connections, ensuring a responsive web design (strategy 2.1) was the second most influential strategy, as responsive design allowed the intervention to be available on various mainstream device types (strategy 1.17) and enabled access to a myriad of technological features (considerations 2A-2G), including options that potentially benefit people with physical impairments (consideration 1I). The third most influential strategy, with 6 outgoing connections, was ensuring that the tools were compatible with alternative technology access methods, such as eye gaze, voice, or touch, as opposed to only standard mouse and keyboard peripherals (strategy 1.6). This strategy had the identified benefits of facilitating technology access for people with ABI with sensory (consideration 1J), physical (consideration 1I), and communication (consideration 1B) impairments, and facilitating the input of patient-reported measures (consideration 2J) and progress data (consideration 2K) by people with ABI with accessibility requirements. It was also noted that these accessibility requirements should be considered in tandem with the need to avoid conveying a sense of penalty and judgment (strategy 1.11), such as standard speech-to-text devices being unable to positively acknowledge dysarthric speech. The fourth most influential strategy, and the first strategy to focus on close others, was strategy 4.10, to “facilitate buddy or peer support for close others.” This had the potential to address the 5 identified assumptions and requirements of close others (considerations 4D-4H), including participating in CPT and overcoming performance anxieties around communicating effectively with someone with ABI.

Within the identified network, there were 6 implementation strategies that each influenced 4 other strategies or considerations. These included suggesting that speech-language pathologists refer people with ABI and their communication partners to the tools (strategy 3.3), providing positive feedback of progress (strategy 4.5), ensuring that tools are available on mainstream device types (strategy 1.17), providing a setup checklist (strategy 4.2), streamlining the number of steps for users (strategy 4.3), and providing reminders to close others (strategy 4.11). Of the 90 remaining nodes, there were 9 (10%) nodes with 3 outgoing connections, 24 (27%) nodes with 2 outgoing connections, and 31 (34%) nodes with 1 outgoing connection. A total of 26 nodes received only incoming relationships, including 25 considerations and the aforementioned strategy (2.8) of tailoring supply models.

## Discussion

### Principal Findings

This study aimed to support the implementation of the Social Brain Toolkit by coproducing new implementation knowledge with people with ABI, close others, clinicians, and leaders in digital health implementation. The first specific aim was to identify stakeholders’ implementation priorities from the NASSS framework [55]. Stakeholders prioritized the investigation of the (1) target condition, (2) technology, (3) value proposition, and (4) adopters of the Social Brain Toolkit. The second specific aim was to identify implementation considerations in each of the top 4 prioritized domains. Stakeholders identified a total of 48 unique digital health implementation considerations across all domains and subdomains. The third specific aim, to coproduce implementation strategies tailored to these considerations, yielded 52 unique, tailored implementation strategies across all domains and subdomains for both developers and clinicians. A collaborative autoethnography revealed both the benefits and challenges of coproducing new implementation knowledge within traditional academic systems. The fourth post hoc aim was to explore potential interrelationships between the identified implementation considerations and strategies. A post hoc network analysis presented a network of 172 unique relationships between the 100 identified considerations and strategies, with highly connected strategies, or “attractors” within the CAS offering data-driven prioritization guidance for implementation effort.

### Coproduction of Implementation Knowledge

To what was previously systematically evaluated to be a sparse evidence base [41], the 48 stakeholder-identified implementation considerations in this study contribute the first extensive compilation of considerations for web-based psychosocial interventions for people with ABI. In particular, this same systematic review [41] identified that the complexities within the first priority domain of the “condition” of ABI have traditionally been simplified via participant exclusion criteria to establish a clear value proposition for interventions (stakeholders’ third priority domain). By contrast, stakeholders in this study identified 14 implementation considerations and 17 implementation strategies in the top priority domain, as well as 4 demand-side and 4 supply-side considerations (Table 4) for the Social Brain Toolkit and 13 tailored strategies to directly address these 8 considerations. There was also guidance on how to target the strategies to specific stakeholders, including people with ABI, close others, clinicians, and health services (Textbox 1). Therefore, the study both enacted and supported the review conclusions to collaborate with stakeholders to increase implementation knowledge.

The 52 tailored strategies identified in this study were largely consistent with generic implementation [77] and sustainment [78] strategies but provided more specific implementation knowledge for ABI and digital health. For example, the general recommendation to “develop strategies with patients to encourage and problem solve around adherence” [77] was addressed through 9 unique strategies (4.1-4.9) to support people with ABI and a further 5 strategies (4.10-4.14) to support close others and clinicians. The generic recommendation to “increase demand” [77] was extended with very specific guidance on which value proposition messages were most important to stakeholders and how, when, and to whom these might be communicated. Thus, these strategies may provide more specific guidance for other digital health implementation endeavors to support people with ABI, as well as digital health implementation more broadly [34,35]. Furthermore, the identified implementation strategies were immediately relevant for current and future iterations of the Social Brain Toolkit [41].

### Collaborative Autoethnography of the Research

Positive final reflections of the reciprocal benefit in coproducing implementation research corroborated previously published coauthor reflections in the study protocol [10]. Reflections on the emotional nature of the writing stage of research were also consistent with the emotion described during data collection [10], supporting the use of trauma-informed principles in coproduced research [79,80]. Researcher experiences appeared to corroborate suggestions that research practices, cultures, and structures encumber the process of coproduction, making it challenging to achieve the aspirations of coproduction [81]. Potentially of particular interest to researchers seeking to coproduce research with people with ABI was the experience of predatory email invitations being sent to all authors, which may warrant proactive warning, education, and follow-up, given the particularity of this type of scam to academia and the known potential vulnerability of people with ABI [25].

### Complexity of Implementation

In addition to the complex network revealed by the VNA, the findings of this study illuminated a clear way forward [69]. A complexity-informed approach identified that user and persona testing (strategy 4.1) may be the single most impactful leverage point to support digital health implementation for people with ABI, reflecting findings from a recent systematic review highlighting the potential to address the complexities for “adopters” [41]. The fruitful use of VNA in this study directly addresses an identified need in implementation science for data-driven methods to prioritize and select implementation strategies from long lists of possibilities [82], especially given potential time and resource constraints. In addition, a focus on strategy 4.1 to target diverse considerations in the “condition” domain offers specific guidance on how to address a previously identified gap in implementation knowledge for people with ABI who have comorbidities and additional challenges [41], specifically, how to practically address the complexity of ABI, rather than reduce it through participant exclusion criteria. It is possible that analogous synergies may be identified and exploited for other digital health interventions and other populations.

The results also provide much-needed guidance on how to select an appropriate theoretical underpinning for future studies [41,83], as the critical position of user and persona testing in the network highlights the value of user-centered design [84]. The sociotechnical focus of the 2 most well-connected “attractors” within the system highlights the importance of using a technology-specific, as opposed to a generic, implementation framework. Therefore, this study supports and contributes methodological knowledge on how to conduct a sound integration of complexity science and implementation science [44] to prioritize digital health implementation strategies and targets.

### Study Strengths

This study has several strengths, primarily in relation to stakeholder input throughout the research. First, this study provides a multidimensional understanding of digital health implementation from the perspectives of people with ABI, their close others, clinicians, and leaders in digital health implementation, with some strategies identified specifically by certain subgroups (Multimedia Appendix 1). In addition, stakeholder prioritization of the study targets enabled research efforts to be directed toward the implementation questions that are most important to end users. Complementarily, stakeholder input included a discussion of preliminary findings from a prior systematic review [41] and the rigorous integration of the NASSS framework [55] from data collection to interpretation. This arguably managed the “balance to be struck between a paternalistic overemphasis on the research literature and a disregard of evidence...Selection and tailoring methods are guided by the best available theory and evidence, while preserving the benefits of stakeholder engagement and preference” [37].

An additional strength of this study was its methodological pluralism. By drawing on the complexity-informed NASSS framework [35] and methods such as network analysis, additional insights could be gained, which maximized the benefit of the initial list of implementation findings, providing new insights into the nature of and potential prioritization opportunities within digital health implementation for people with ABI. In doing so, this study also contributed an applied example of how a critical realist approach can be used to rigorously manage the conceptual, theoretical, and methodological tensions between complexity and implementation sciences to support the practical implementation of real-world interventions.

### Study Limitations

This study also has several limitations. First, although a focus on domains 1 to 4 reflected stakeholder priorities, additional implementation considerations and strategies from domains 5 to 7 were not reported, despite there being less research evidence to guide implementation in these domains [41]. With regard to complexity, this limitation is notable, given known interactions between domains 5 to 7 and the results of domains 1 to 4. For instance, professional expectations and regulations in the “wider system” (domain 6) would also interact with the reported results. In domain 6, although “client stories” (strategy 3.2) were directly requested by people with ABI and close others to convey the “value proposition” (domain 3) of the Social Brain Toolkit, the implementation of this strategy is not straightforward when considered in relation to the Code of Ethics of Speech Pathology Australia [85], which stipulates that all advertising “must not use testimonials / reviews. The use of ‘client stories’ may constitute testimonializing, which is prohibited” [86].

Second, given that ABI is especially prevalent in low- and middle-income countries [87], there was a noteworthy lack of representation from these countries, where additional complexities and strategies may have been identified. In addition, the results should be interpreted with consideration of the majority representation of Australian participants. For instance, strategy 3.12 was a recommendation of hierarchical pricing that demands least to no payment from people with ABI and most funding from governments and insurers. The identification of this strategy may have been influenced by the Australian context, in which national Medicare and insurance schemes may increase expectations of government support in a way that differs from countries and markets where self-funding health care is the norm.

Finally, although the exploratory and mechanistic focus of this study is consistent with complexity science and its distinctive quality criteria [50], the identified implementation strategies and considerations and their interrelationships have not yet been empirically tested or observed and would, therefore, be considered lower on traditional health research evidence hierarchies [88]. Therefore, future research might consolidate these preliminary findings by empirically testing the identified strategies, considerations, and interrelationships with a more confirmatory aim. This may also eventually allow for more in-depth network analytical methods in future, such as the weighting of certain links based on a deeper understanding of their importance or the use of alternative measures of centrality when digital health implementation mechanisms are better understood. However, it should be noted that a complexity-informed investigation would not expect the findings of this study to be precisely replicable at 2 points in time, given the qualitative nature of the data and the dynamic nature of complexity. Therefore, research resources may be better directed toward more practical implementation efforts, namely user and persona testing (strategy 4.1) for profiles that were not directly represented in this study.

### Conclusions

People with ABI, close others, clinicians, and digital health leaders coproduced new knowledge of digital health implementation considerations for adults with ABI and their communication partners, as well as strategies tailored to address them. Several of the identified strategies may be applicable to other interventions for ABI, as well as interventions for conditions other than ABI, addressing current gaps in digital health implementation knowledge more broadly. Although this study was limited by its focus on 4 NASSS domains and the underrepresentation of some participant demographics, the wealth of immediately actionable implementation knowledge generated is aligned with the prospect of including stakeholders in the coproduction of implementation research, with mutually beneficial outcomes for stakeholders and researchers. A complexity-informed theoretical underpinning and a post hoc network analysis revealed that digital health implementation for people with ABI, their close others, and clinicians is a complex phenomenon. Traditional implementation science methods may therefore benefit from leveraging complexity-informed frameworks such as the NASSS and methods such as VNA to more fully understand, better prioritize, and more effectively address digital health implementation needs.

## Appendix

Multimedia Appendix 1 Full descriptions of all 100 considerations and strategies.

## Abbreviations

ABI: acquired brain injury
CAS: complex adaptive system
CPT: communication partner training
NASSS: Nonadoption, Abandonment, Scale-up, Spread, and Sustainability
TBI: traumatic brain injury
VNA: visual network analysis

## Data Availability

The data sets generated and analyzed during this study are not publicly available to protect the privacy and confidentiality of participants as per human research ethics approvals. However, reasonable requests to the first author will be considered.
