# Supplementary material for: Digital Health Implementation Strategies Coproduced With Adults With Acquired Brain Injury, Their Close Others, and Clinicians: Mixed Methods Study With Collaborative Autoethnography and Network Analysis
Source: J Med Internet Res. 2023 Sep 19;25:e46396. doi: 10.2196/46396 (PMC10548320; doi:10.2196/46396)
Supplement: Multimedia Appendix 1 [file jmir_v25i1e46396_app1.docx]

### First priority domain: Condition

#### Nature of ABI

##### Consideration 1A - Stage of recovery

People with ABI and their close others communicated a sense of urgency to accessing communication interventions. For example, a person with ABI reflected; “I would have benefitted from access to a constructive rehabilitative task like the Toolkit immediately after discharge” (Person with living experience of ABI 10, Focus Group 3). Likewise, communication partners expressed desire for earlier support;

- *“make it early as possible. As early as possible. So, let's say when people [are] discharged from hospital to rehab [or] something like that, make it available.” (Communication partner 6, Focus group 6).*
- *“it was only after [Friend with ABI] was recovering and everything was getting better that I realized maybe there were things I could have done earlier that may have been more helpful. […] you need to have the right input at the right time, because then it will be motivating.” (Communication partner 1, Focus Group 2).*

However, given the severity of ABI was greatest “in the early days” (Person with living experience of ABI 3, Focus Group 2), people with ABI, their close others and clinicians acknowledged it may not always be feasible for the person with ABI to participate during the acute stages of recovery;

- *“I definitely don't think I would have been able to use any Tools when I was still in hospital, or even just out, because I lost all speech and many other things. I was just focusing on getting through another day, so giving me any work or anything, I just know in hospital it was beyond me at that time.” (Person with living experience of ABI 2, Focus Group 3).*
- *“seeing them in the quite early acute times, that insight that they even have a problem, it's so low, typically, and their initiation is impaired and thus they want to do it, but they just can't get started with it. So, the population I work with, I really don't think it's realistic that they could do homework or engage of their own accord.” (Speech-language pathologist 4, Focus group 1).*

This consideration may be investigated through user or persona testing (Strategy 4.1), outlined later in the *‘Fourth priority domain: Adopters’* section.

###### Strategy 1.1 – Disseminate the Social Brain Toolkit to communication partners while the person with ABI is in the acute stage of recovery, and to people with ABI between discharge and rehabilitation

People with ABI, their close others and clinicians therefore described the acute stage of recovery as an opportunity to first focus on supporting close others;

- *“[Son with ABI] can't use [the intervention] until he gets home, until he is able to talk, because in rehab[ilitation], they [have to] teach him to speak in ABCs [again], [therefore] everything, in the beginning [of his recovery, is] not suitable for him, but [it would be] very, very useful for family members.” (Communication partner 6, Focus group 6).*
- *“Probably not straight after I’d had the accident [for me], but my parents, my friends, my best friend, who came and saw me every day at the rehab[ilitation] center, every day at the hospital, he could have done this straight up, so the support that came from it, he'd have been able to tell my parents, and my parents would be able to tell my sisters, and my sisters would be able to effectively understand what's going on with me” (Person with living experience of ABI 3, Focus Group 2).*

Clinicians and close others noted that close others that it was beneficial to introduce the intervention asynchronously even before close others had developed sufficient readiness and insight into their need for support;

- *“that’s [a] very important point that when we start to introduce this one, they say, "Hey, we [have] got online [support] here". […] some sort of introduction and get people in in the first place. And then slowly, slowly [they realize the need], and they say, ‘that's a very [good] idea, a very good help’, and they started to get in. Yeah, if I don't have enough support, how can I help my son?” (Communication partner 6, Focus group 6).*
- *“if I could introduce it as an acute, subacute inpatient clinician, give them the link, then they can do it, [even if] it means nothing [now], but they can come back to it 6 months down the line, [and realize]; “Oh that's what she was going on about”, and future clinicians can kind of refer back in as the loved ones’ clinical presentation changes.” (Speech-language pathologist 4, Focus group 1).*

This need and stakeholders’ preferred messages and methods are described further in the *‘Third priority domain: Value Proposition’* section (Strategy 3.2, 3.3, 3.5).

###### Strategy 1.2 – Suggest that speech-language pathologists can help determine whether a tool would be suitable for a person with ABI and their communication partners

Given the complexity of potentially balancing desire and readiness for intervention, it may be helpful for the clinician to assess whether the intervention was appropriate;

- *“[Rehabilitation hospitals] need to do a bit of an assessment […] and try to work out who's actually tech-savvy enough and who has progressed enough. I think there are lots of people I met in [Rehabilitation hospital name] who didn't have language, but there were people who did. The speech therapist would have to do the assessment.” (Person with living experience of ABI 1, Focus Group 2).*
- *“I would agree […] assessment would be appreciated.” (Communication partner 7, Focus Group 2).*

This strategy is thus connected with the suggestion that speech-language pathologists can refer people with ABI and their communication partners to the tools (Strategy 3.3) in the *‘Third priority domain: Value Proposition’* section.

##### Consideration 1B – Concentration impairments

People with ABI noted a need for intervention to cater for impaired concentration;

- *“I don't have a great concentration and my comprehension isn't great either. […] being able to go back 3 pages [would be challenging]” (Person with living experience of ABI 10, Focus Group 3).*
- *“you know how therapists only have 20 to 30 minutes to give you assistance, but you're only efficient for about 5 of those minutes? My mom took the role of the therapist to basically do my speech exercises and my physio[therapy] exercises 20 times throughout the day, and for only like 2 or 3 minutes each time. So, [that was] far more efficient, because it’s less time, but it’s efficient for the entire time.” (Person with living experience of ABI 3, Focus Group 2).*

This consideration may also be investigated through user or persona testing (Strategy 4.1), outlined later in the *‘Fourth priority domain: Adopters’* section.

###### Strategy 1.3 – Create bite-sized modules that allow for breaks

Leaders in digital health implementation described a need to streamline the content and “use the technology to allow you to create bite-sized activities.” (Digital health implementation leader 4). Indeed, “The fewer the questions, the better. Every time there is a change in the flow, you might distract somebody.” (Digital health implementation leader 1). This was corroborated from stakeholder perspectives. Firstly, the need to streamline content was summarized by the following exchange between a clinician familiar with predecessor programs and a person with living experience of ABI;

- *“the original versions of these programs, like TBI Express, they're brilliant, but they contain a lot of extra information, like a lot of detail about social rules and a lot of just sort of quite detailed information, and I think if it's not relevant to the person you're not going to get great engagement - I found, anyway. So, as a clinician, I tend to sort of leave out those bits anyway when I do these sorts of programs, unless it's relevant.” (Speech-language pathologist 1, Focus group 6).*
- *“as a brain injury person, I back that up. It's hard enough doing some basic stuff, we don't need things to make it more complicated.” (Person with living experience of ABI 6, Focus Group 5 and 6).*

Secondly, it was noted that breaks should be facilitated by creating sections that could be saved and returned to in a self-paced manner;

- *“you do 1 activity and maybe you could get up and go and then come back to it maybe tomorrow” (**Communication partner 4, Focus group 7).*
- *“don't push people, right? Just let [them] enjoy it through your page. Whatever you want to [do], you can save it […] and come back, so that they do it at their own pace.” (Communication partner 6, Focus group 6).*

###### Strategy 1.4 – Provide simple, clear, and consistent user interfaces

People with living experience of ABI emphasized the need for “as simple an interface as possible” (Person with living experience of ABI 10, Focus Group 3), leveraging format and layout to create the most intuitive user experience;

- *“it's got to be big and clear and not messy because [otherwise] it becomes confusing and overwhelming.” (Person with living experience of ABI 6, Focus Group 5 and 6, agreed by Person with living experience of ABI 4, Focus Group 5).*
- *“it's got to be standardized so that every type of communication is done in a certain font so that when you see a certain font, you know that that’s relevant.” (Person with living experience of ABI 4, Focus Group 5).*

##### Consideration 1C – Communication impairments

In addition to cognitive difficulties, it was noted that ABI could introduce impairments in receptive language comprehension. From the perspective of a person with ABI, a primary consideration “is that it’s asking you some fairly difficult questions and the capacity for you to deal with those questions has got nothing to do with the technology that you’re using, it's whether or not your brain [can get] around the idea of the question that you’re being asked.” (Person with living experience of ABI 9, Focus Group 5, agreed by Person with living experience of ABI 6, Focus Group 5 and 6). Therefore, “You might find that they're getting to a certain page in the process and that page, as it turns out, is just accidentally a little bit too wordy or complicated.” (Digital health implementation leader 5). It was also noted that there could be expressive language difficulties as a result of ABI. For example, “as part of aphasia – I can remember distinctly having difficulty spelling and I had a huge difficulty with writing.” (Digital health implementation leader 3 with living experience of ABI). This consideration may be investigated through user or persona testing (Strategy 4.1), outlined later in the *‘Fourth priority domain: Adopters’* section.

###### Strategy 1.5 – Use simple language

To reduce complexities introduced by receptive language difficulties, “the big thing with your brain injury patients you've got to bring it down to the basic language.” (Communication partner 5, Focus group 6). A person with experience of both ABI and digital health implementation noted;

*“the language [in the intervention] should be very basic […] [For example] the question can be quite long and will sometimes have a double negative in it - It’s done in psychology to try and make sure that I guess they’re getting consistency - but with people with brain injury [laughs], it’s actually really actually a negative thing, because they won’t be able to comprehend that there’s a double negative in there. So, it’s not that they’re not telling the truth, or not deliberately being consistent, but they just don’t understand the question [in the way] that it’s phrased. So, I think [the intervention] needs to be as empowering as possible in the [information] it provides.” (Digital health implementation leader 3 with living experience of ABI).*

###### Strategy 1.6 – Ensure compatibility with alternative technology access methods (eg, eye-gaze, voice, or touchscreen)

Given the various ways ABI may affect expressive communication, it may be helpful to leverage technology to give people with ABI alternative ways to input intervention data wherever possible. As observed by a clinician;

*“with any sort of online platform, people who use AAC [Augmentative and Alternative Communication] are probably overlooked a lot of the time, so I think ideally it could be adapted according to what the user needs. So, if they're using a text-to-speech device or an eye-gaze device, that there would be scope for that to work” (Speech-language pathologist 1, Focus group 6).*

Overall, it was considered important,

*“[Just that such difficulty] has to be taken into consideration, but I think giving the option is wonderful, because some people can type and write better than they can communicate orally. So, it just depends, and some people can read but can’t have their communication output, so [I think it is beneficial having] all the different options, so maybe if they have trouble typing, they could use an audio tool. Sometimes doing things like multiple choice can be easier as well, just to get some momentum going as well.” (Digital health implementation leader 3 with living experience of ABI).*

##### Consideration 1D – Memory impairments

Among the cognitive difficulties that might arise from ABI, memory difficulties post-injury could affect the implementation of an intervention. Due to memory impairments;

*“compliance for people with brain injury is one of the hardest things. It’s not a deliberate want to not comply or turn up for appointments. It’s that forgetfulness”. (Digital health implementation leader 3 with living experience of ABI).*

For example,

*“[Spouse with ABI] has to be reminded. So, he's not going to do a speech program or anything online unless there's someone who's going to prompt him. I imagine that lots of people with a brain injury would be similar; they just don't remember that it's there to do.” (Communication partner 4, Focus group 7).*

This consideration may be investigated through user or persona testing, outlined later in the *‘Fourth priority domain: Adopters’* section.

###### Strategy 1.7 – Leverage technology to reduce user memory requirements (eg, automation, repetition, or guided structure)

To address this issue in the design of a digital health intervention, “you have to take the memory out of it.” (Digital health implementation leader 3 with living experience of ABI). Leaders in digital health implementation provided various ways technology could be leveraged to support users, particularly in the login process;

- *“They log in once and they should be logged in from it forever from then. So, you should just remove any kind of obstacles to using it.” (Digital health implementation leader 7).*
- *“there’s a possibility that people might forget how to access it, how to get to that site, so […] I would get them to tag it as a shortcut and have it as a bookmark on their browser up the top, to have reminders, that it comes up as a reminder on either an email or something that they check regularly and it prompts them to do it”. (Digital health implementation leader 3 with living experience of ABI).*
- *“making sure that the whole sequence of events is very guided and it's very clear where they're up to and what they need to do next, rather than them having to recall steps in the process.” (Digital health implementation leader 5).*

Additionally, the timing of tasks should also be designed with consideration of “how long after you’ve provided the information you have to answer the question, because the information doesn’t necessarily get retained for all that long” (Digital health implementation leader 3 with living experience of ABI).

##### Consideration 1E – Impairments in emotional regulation

Another cognitive impairment introduced by the condition of ABI,

*“would be the emotional regulation, and where people with pre-frontal injury will have significant difficulty reading emotion, managing their own emotion, and so on. Anger, irritability, of course, would contribute.” (Digital health implementation leader 1).*

From the perspective of a person with living experience of ABI, even “very, very, mild” ABI could impact a person’s capacity to interact with a web-based intervention;

*"past a pretty low threshold, it would become difficult quite quickly. That certainly would have been difficult for me. […] when there were delays, lags, small-scale complications, [it] would have grown very frustrating." (Person with living experience of ABI 7, Focus group 4).*

This consideration may also be investigated through user or persona testing (Strategy 4.1), outlined later in the *‘Fourth priority domain: Adopters’* section.

###### Strategy 1.8 – Ensure fast and easy usability

With irritability and emotional regulation in mind, intervention developers should focus on improving usability and user experience, ensuring technical speed to avoid lags, and “making sure that that patient journey to get to that [telehealth video] call is as simple as possible, which isn't easy.” (Digital health implementation leader 9). Leaders in digital health implementation consistently singled out that; “the usability of the technology, that has to be a high priority, to make sure it’s usable” (Digital health implementation leader 8).

##### Consideration 1F - Self-esteem

An additional complexity introduced by ABI was its potential impact on a person’s self-esteem. From the perspective of people with ABI, close others, clinicians and developers, this complexity must be managed effectively to avoid user abandonment;

- *“should we have any difficulties, even ones that I perceive to be quite minor, […] it’s a total shut down, disengage, and I've got no hope of getting back from that.” (Speech-language pathologist 4, Focus group 1).*
- *“if there's too much pressure, they will just back out of it.” (Communication partner 5, Focus group 6).*
- *“some of those tasks would just remind some of the members about some of the things they can't do.” (Person with living experience of ABI 2, Focus Group 3).*
- *“you may think it's even a reasonable question, but if they think it's hard, then that'll be discouraging, and it will essentially be a [punisher].” (Digital health implementation leader 1).*

This consideration may also be investigated through user or persona testing (Strategy 4.1), outlined later in the *‘Fourth priority domain: Adopters’* section.

###### Strategy 1.9 – Ensure achievable task difficulty

To minimize this risk, developers should be mindful to set users up for success in task design:

*“what my experience is, is that you've kind of got one chance to get it right, and it has to be a successful experience the first time that they go on there, so that you succeed, and your confidence is still there. […] I need to really structure my re-introduction [of social media] very carefully to ensure success [...] So I might get their phone and take off every single app except the one I want from the home screen. So that they can only click on the target we're working on, and that we don't have these kinds of failures. […] my feedback would be, I think this is really important, because should we have people engaged, they need to feel successful from the get-go. It needs to be as easy as possible, because once you lose them, you really, I really lose them” (Speech-language pathologist 4, Focus group 1).*

###### Strategy 1.10 – Maintain a supportive tone and language

To avoid abandonment and discouragement, people with living experience of ABI, their close others and clinicians suggested numerous specific phrases that could convey support;

- *“if you haven't got something done or can't get something done, then it’s about, ‘Well how can we help you with this?’, ‘Because we still want you to get the maximum benefit from this within the bounds of your individual situation, how can we help you?’ and so it's about remaining engaged and remaining motivated as opposed to ‘oh well, I failed, so I might as well just give up then’.” (Communication partner 3, Focus group 4).*
- *“it's a good idea [to send reminders], but not put it in [a phrase such as] ‘You've still got to do this’. The reminder could say “okay, this one’s coming up next’, instead of ‘you've still got to do this’, ‘Just a reminder that this one’s coming up next’. Yeah, so just be very careful just with your wording […] Maybe even say something like ‘today, ohh, you wanted to do...’, ‘We're just reminding that you wanted to spend some time on this today’, […] with [Person with living experience of ABI 6, Focus Group 5 and 6], sometimes he might think things I say might be passive-aggressive. [You’ve] Got to keep it very easy-flowing and so, I say, it comes back to your wording. I definitely believe in the reminders, but ‘just reminding you that you were going to set a time sometime today, [and] if you can't, just reschedule’” (Communication partner 5, Focus group 6).*
- *“the reminder could also be phrased in terms of the goal, so rather than ‘you've got this appointment’ it could be, ‘don't forget to have a chat about…You know, your favorite movie today’” (Speech-language pathologist 1, Focus group 6).*
- *“Don't forget’ is probably an ideal wording.” (Person with living experience of ABI 6, Focus Group 5 and 6).*
- *“There could be just, ‘when you get a chance, this is where you're up to’ or something like that?” (Communication partner 5, Focus group 6).*
- *“Suggest' there is the key. The word 'suggest' for me, like I lock it in, but other people aren't that way inclined.” (Person with living experience of ABI 6, Focus Group 5 and 6).*

This thoughtful language was necessary, because, as described by a close other of a person with ABI;

*“[Spouse with ABI] feels like it's each time we have to see a doctor or a therapist or something, he's being judged, and it's really hard to convince him otherwise in a lot of cases. He doesn't want to participate because he's not achieving as he thinks he should be.” (Communication partner 4, Focus group 7).*

###### Strategy 1.11 – Avoid conveying judgment or penalty

In addition to using a supportive tone wherever possible, it was recommended that developers avoid conveying failure or judgment in any aspect of the intervention. For example, when providing feedback on responses to questions; “Instead of saying, “wrong!”, it might be, “hey, try again. Let's try again.” (Digital health implementation leader 1). In sum, “I'd really emphasize the principle of errorless learning” (Digital health implementation leader 1). The concept of avoiding conveying error extended beyond intervention content to accessibility options:

*“I don't know whether recording him, or like voice-to-text [would be effective], because some of his speech is not very clear yet. […] if it types the word incorrectly, then that would just be more disappointing to him, that he wasn't able to say it clearly enough for the computer to understand. […] he would be someone who would respond negatively to that. Whereas he can spell, and when he types, he can spell all perfectly fine, it just takes a really long time, but at least it's correct.” (Communication partner 4, Focus group 7).*

This strategy could therefore be considered in relation to Strategy 1.6 to ‘Ensure compatibility with alternative technology access methods’.

In addition to avoiding judgment of correctness, stakeholders advised there should be no judgment of completeness;

*“it needs to be just presented in such a way that this is about ‘we understand that everybody has different needs. Everybody has different effects of their particular brain injuries, and so if you haven't managed to get this done, or you haven't managed to complete this part of a course, then you haven't failed. Don't give up because this is about...’ so it really has to walk a fine line between a learning useful tool, but not into a classroom pass-fail type situation.” (Communication partner 3, Focus group 4).*

Fellow focus group members strongly agreed that “There should be no penalties” (Person with living experience of ABI 7, Focus group 4) and “no guilt tripping [about whether people were] getting things done” (Speech-language pathologist 2, Focus group 4).

###### Strategy 1.12 – Empower people with ABI with the option to provide feedback about the tools

An additional way the self-esteem of people with ABI could be supported is to empower intervention users to contribute their feedback:

*“the more it feels like they're a part of a constructive project, as opposed to just subjected to testing, the more they're going to feel motivated to come back. […] it's like putting on the brakes to the cognitive tests and reframing it, so you've got something to contribute, [which] takes the pressure off the person.” (Person with living experience of ABI 7, Focus Group 4).*

However, it was recommended to;

*“always just leave it as an option [to enter feedback], because if people wanted to give feedback and felt strongly, ‘hey, there are opportunities for improvement of things you haven't thought’ [they could then do so], but I would never make it mandatory that ‘you have to fill this out’, because I know that for certain members of my [group with aphasia], that would stress them out, because they would not be able to fill out the thing, because some of the members, they can't write at all, so asking them to do that would be more stressful. So, I would never make ‘oh, you have to make this feedback’ mandatory. Personally, I would just give it as ‘Hey there's an option if you would like to give feedback but you don't have to.’” (Person with living experience of ABI 2, Focus Group 3).*

This returns to the concept of avoiding judgment and penalty (Strategy 1.11). It is also a way to maximize user autonomy in the *‘Third priority domain: Value proposition’* section (Strategy 3.11).

#### Comorbidities

##### Consideration 1G - Fatigue

A common comorbidity of ABI that was especially observed by communication partners was that;

*“tiredness and fatigue is very taxing on people with a brain injury, and gee, I feel for them, and the simpler it could all be and the less taxing the whole exercise is, the more effective it will be.” (Communication partner 10, Focus group 7).*

This comorbidity may be a consideration for user or persona testing (Strategy 4.1).

###### Strategy 1.13 – Suggest completion in the morning

In addition to design efforts to reduce user burden (Strategy 4.3) and facilitating breaks through short modules (Strategy 1.3), the impact of fatigue may mean people with ABI may benefit from clinicians scheduling morning telehealth calls (Speech-language pathologists 3 and 4, Focus group 1) or reminders to complete self-directed work in the morning (Person with living experience of ABI 5, Focus Group 3).

##### Consideration 1H - Pain

The pain of comorbid physical injuries may mean that standard ergonomic setups and working periods should not be assumed. For example,

*“a big computer setup, if you had it on a desk or something with webcams and blah-blah-blah, in [Spouse with ABI]'s case, it wouldn't be great because he had quite severe physical injuries as well […] and the pain makes him very fatigued. […] he's got like a recliner chair thing. He can't be at a desk chair for long periods of time as well. That just wouldn't be appropriate for him because he'll just get too tired and in too much pain and then he won't take any of it in. That's the other thing to consider.” (Communication partner 4, Focus group 7).*

This aligned with Strategy 1.3 to facilitate breaks between short modules. This comorbidity may also be a consideration for user or persona testing (Strategy 4.1).

##### Consideration 1I - Physical impairments

People with ABI, close others and clinicians noted varying types and levels of physical disability that could affect a person’s interaction with technology. For example; “[Daughter with ABI]'s got like a weakness on one side and a tremor on the other” (Communication partner 11, Focus group 7). Physical impairments like hemiparesis could make it harder to handle a device;

*“it's just very important that the tablet is in a position that it doesn't move, not on their laps or whatever, because it's also really hard then for them to sometimes control due to paralysis, and if it drops, if you do lose connection, then you cannot reconnect again. So, it has to be very stable.” (Speech-language pathologist 5, Focus group 1).*

Device choice (Strategy 2.1) is discussed further in the ‘*Second priority domain: Technology’* section, as is setup advice (Strategy 4.2) in the ‘*Fourth priority domain: Adopters’* section. This comorbidity may also be a consideration for user or persona testing (Strategy 4.1).

Physical impairments could also encumber the use of standard peripherals or onscreen interfaces;

- *“typing and using a mouse and things would be difficult for [Spouse with ABI] because the hemiplegia is on his right side and that was his dominant hand.” (Communication partner 4, Focus group 7).*
- *“pressing and dragging kind of actions are really hard as well, because they don't have the muscle tension” (Speech-language pathologist 5, Focus group 1).*

Typing may be enabled by using the non-dominant hand, alternative accessibility options (Strategy 1.6), or allowing more time (Strategy 1.14);

- *“he does do it with his left hand.” (Communication partner 4, Focus group 7).*
- *“I've lost control of the right-hand side so therefore my capacity to answer is much less than what it used to be and so I’d much prefer to voice that answer than type it, but I can [type] – it just takes longer, that’s all. (Person with living experience of ABI 9, Focus Group 5).*

##### Consideration 1J - Sensory impairments

Some people with ABI reported developing hearing and vision impairments from their ABI. Developers may therefore need to consider sensory impairments when developing an intervention, including through user or persona testing (Strategy 4.1).

###### Strategy 1.14 – Avoid placing time limits on tasks

Therefore, as outlined in Strategy 1.6 to offer multimodal options;

*“breadth of options is important ultimately. Don't rely on typing with a keyboard. Actually, there's a really important principle actually around this as well, around time. Don't put time constraints on things. Just as an example of that; a lot of software has timeouts. That can be a huge problem for people with disability because they just can't physically complete the process in 5 minutes.” (Digital health implementation leader 5).*

###### Strategy 1.15 –Ensure multimodal accessibility when designing tasks (eg, avoid designing tasks that can only be completed visually)

As a bare minimum, leaders in digital health implementation advised web-based platforms should comply with current Web Content Accessibility Guidelines [67].

*“However, the problem with that is that that doesn't guarantee accessibility. It really helps a lot, but it doesn't actually guarantee it. So, the secondary level to this is, there's not really a replacement at current for what we call accessibility testing. So that's actually having people sit down generally under observation to observe them using the system” (Digital health implementation leader 5).*

User or persona testing (Strategy 4.1) is discussed in detail in the ‘Fourth priority domain: Adopters’ section. The input of people with living experience of ABI is invaluable to accommodate impairments specific to ABI. For example, when making video content, a co-author with living experience of prosopagnosia, or “face-blindness” after ABI, suggested that developers could choose video actors who are as physically different as possible, such as choosing a man and a woman of different ethnicities and ages.

As an overall principle during task design,

*“multichannel is ultimately the key thing. So, look at a really basic example, don't design things that can only be completed based on a visual search because that would preclude a blind person.” (Digital health implementation leader 5).*

An advantage of web-based platforms was their compatibility with existing accessibility tools within browsers and devices; “Generally they would have [screen readers] installed themselves if they had a vision impairment because it's something that helps them navigate the entire internet, it wouldn't be just your website.” (Digital health implementation leader 5).

#### Sociocultural factors

##### Consideration 1K - Nature and availability of social support

It was noted that social support was an important consideration for both digital health access and healthcare access more broadly;

*“It's a bit of a cascading snowball effect at times - because of these injuries or other conditions, people lose community supports. They lose carer supports. Their mental health declines and becomes a snowball that makes reaching out and accessing care harder until it gets to a tipping point.” (Digital health implementation leader 9).*

It was noted that it should not be assumed that all people with ABI will have close others to help or join them in completing an intervention together, with paid carers becoming more likely communication partners for some people with ABI (Speech-language pathologist 4, Focus group 1). Another clinician reported experiencing a close other dismissing a person with ABI’s need for intervention and gatekeeping access to webcam technology for telehealth calls (Speech-language pathologist 3, Focus group 1). This consideration is complementary to reports by people with ABI and their communication partners that close others are a major part of the rehabilitation process (Consideration 4F) and could be considered in user or persona testing (Strategy 4.1) in the *‘Fourth priority domain: Adopters’* section.

###### Strategy 1.16 – Suggest seeking multidisciplinary support beyond speech-language pathology if needed

Given the communication focus of the Social Brain Toolkit, a particularly pertinent consideration for clinicians is that;

*“when speech pathologists are using these types of tools with clients, especially when you're working on communication with a conversation partner, it can sort of reveal sort of pre-injury relationship or marital or friendship issues. And sometimes I find I need to get people to see a social worker, psychologist, relationship counsellor, before we then can address communication, because I feel like sometimes that's just worth as a clinician, being mindful of.” (Speech-language pathologist 1, Focus group 6).*

##### Consideration 1L - Invisibility and social stigma of disability

The invisible nature of ABI, societal stigma around disability, and the ubiquity of technology in modern society, may result in a desire for discreet “self-service” on mainstream devices; “It would put him off [to have a large keyboard for accessibility]; he doesn't really like anything that draws attention to his disability.” (Communication partner 4, Focus group 7). This profile could be considered in user or persona testing (Strategy 4.1) in the *‘Fourth priority domain: Adopters’* section.

##### Consideration 1M - Socioeconomic situation

The socioeconomic situation of a person with ABI and their families may affect their capacity to access and complete an intervention. For example, some people with ABI may be unable to self-fund an intervention (Speech-language pathologist 4, Focus group 1), and families described having to sell their house due complete financial upheaval after the ABI (Communication partner 6, Focus group 6). Additionally, although people with ABI experiencing homelessness or domestic violence may be able to temporarily access web-based treatments within a rehabilitation setting,

*“the issue would be generalizing once they’d left the hospital if they were being discharged to accommodation that wasn't stable; then you're probably not going to get a lot of maintenance, and if you needed to continue the program, that might be challenging.” (Speech-language pathologist 1, Focus group 6).*

In addition to broader socioeconomic factors, there were several challenges specific to digital health. For example, “we see there's common traits around accessing online healthcare, around who has a smart device, who has a computer and who has an Internet connection.” (Digital health implementation leader 9). This user case could be considered in user or persona testing (Strategy 4.1) in the *‘Fourth priority domain: Adopters’* section.

##### Consideration 1N – Gender differences in healthcare access

Gender differences in the prevalence of TBI may be especially relevant for implementation efforts;

*“young men are disproportionately represented in the statistics who experience traumatic brain injury in particular, and yet that's a population, invariably, who don’t seek treatment. […] if you were able to engage with younger men, that would be great, but I suspect you will have difficulty, not because of your intervention, but because they don’t currently seek support.” (Digital health implementation leader 1).*

This user case could be considered in user or persona testing (Strategy 4.1) in the *‘Fourth priority domain: Adopters’* section.

###### Strategy 1.17 – Ensure tools are available on mainstream device types

Implementation may be facilitated by leveraging mainstream device types such as smartphones and laptops, as “it has to be normalized” (Speech-language pathologist 3, Focus group 1). For example,

*“with clients I would be using on their phone. A lot of my clients won't do something if it's obviously disability-related or ‘outs’ them a little bit. Or you know this, ‘I’m a cool 20-year-old skateboarder dude’. So, if I can get on this phone, like we've just been doing Facebook and then we click over to this, it's all cool, and easy and quick and inconspicuous.” (Speech-language pathologist 4, Focus group 1).*

An additional potential value proposition of the Toolkit in reaching male users is its potential to support person-centered care (Consideration 3F) through discreet self-service on mainstream devices, discussed in the ‘*Third priority domain: Value Proposition’* section.

Overall, the presence of these myriad sociocultural considerations,

*“doesn’t undermine [the intervention’s] value. You maybe just sharpen it. I would actually think that there's some instances where the technology can assist with this. […] It's clearly a challenge but not always a barrier.” (Digital health implementation leader 4).*

### Second priority domain: Technology

#### Key features

##### Consideration 2A - Screen size

Stakeholders identified screen size as important key feature of digital health technology. The primary advantage of computers, and to a lesser extent, tablets, was their larger screen size:

*“If you [have] got lots of information in front of you, you want to have a decent size you can actually do it and be part of it, whereas on the telephone, the screen’s only so small, the tablet screen’s a bit bigger, whereas on the computer the screen’s nice and large, and I kind of enjoy that, insofar as being able to see something in a decent scale as opposed to a tiny scale.” (Person with living experience of ABI 9, Focus Group 5).*

Computer access also enabled the use of multiple monitors;

*“having two screens means you can actually look at everyone across the field of view and you’re not sort of focused on one little screen.” (Person with living experience of ABI 4, Focus Group 5).*

This desire for a larger screen meant smartphones were a non-preferred option:

- *“I’d struggle to see the content on a small device.” (Person with living experience of ABI 10, Focus Group 3).*
- *“phones are no-go for sure for me because they're so small […] everything is so small, you need to go around the tabs and things, [and] if [they are] there at all, everything [is] smashed altogether. It's really hard.” (Speech-language pathologist 5, Focus group 1).*

However, a clinician participant recalled observing that some people with ABI may prefer to scan a smaller screen (Speech-language pathologist 2, Focus group 4).

##### Consideration 2B - Camera

To conduct telehealth video calls, it was reported that high-resolution cameras built into devices was advantageous;

- *“if they're using a laptop with a built-in camera, it'll be a lot easier for them to take a video and then upload it. It's dead simple. Same with an iPad if they're doing the training through an iPad. If they're using a computer that doesn't have a camera for some reason and then they have to record the video with their iPhone or something, it becomes harder.” (Digital health implementation leader 5).*
- *“we did actually end up using her iPad because you can't buy an iPad without camera, so that [overcame the barrier of a caregiver who avoided buying a computer with a camera]. So, I personally I think tablets is probably the way forward” (Speech-language pathologist 3, Focus group 1).*

##### Consideration 2C - Accessibility

A particular benefit of tablets was their option for both direct and indirect access:

- *“with the tablet you can actually have external peripherals, so you can have a keyboard and a mouse.” (Person with living experience of ABI 3, Focus Group 2).*
- *“[the person with ABI] cannot move the mouse to click on the blue thing that she needs to say, ‘OK’, but I'm assuming if she had a tablet, maybe she could just touch the screen.” (Speech-language pathologist 5, Focus group 1).*

This relates to physical (Consideration 1I) and sensory (Consideration 1J) accessibility needs outlined in the ‘*First priority domain: Condition’* section.

##### Consideration 2D - Portability

Although nonpreferred due to challenges with stability and size, portability made tablets and smartphones a suitable backup option;

- *“For me the smartphone’s a last resort, like if you’re going somewhere and you need to do it.” (Person with living experience of ABI 6, Focus Group 5 and 6).*
- *“I prefer to use a computer, but then if I was elsewhere when the call was on or when something was on, I'd be happy to use the telephone or the tablet.” (Person with living experience of ABI 9, Focus Group 5).*

However, portability was not without disadvantages;

*“[Daughter with ABI] would have to have probably a computer that was fixed because she loses her phone every day.” (Communication partner 10, Focus group 7).*

##### Consideration 2E - Stability

The stability of computers was an advantage compared to smartphones and tablets; “I find [the tablet]’s cumbersome because sometimes you've got to find somewhere to place it and sometimes it falls over or falls off the desk or something, whereas the laptop, it’s really stable.” (Person with living experience of ABI 4, Focus Group 5).

This was described in relation to challenges associated with hemiparesis (Consideration 1I) in the ‘*First priority domain: Condition’* section.

##### Consideration 2F - Affordability

Tablets were often singled out as a preferred device, combining key feature benefits with appeal and affordability;

- *“To me, tablet [access is best]; first thing, [it’s] cheaper, you can carry [it] around, you can access [it easily] - the mobile screen [is] very small, [whereas a] tablet is a little bit handy, and you can carry [it] anywhere […] it is very popular, affordable and easy to use.” (Communication partner 6, Focus group 6).*
- *“the size [of a tablet] is better than a phone, they’re cheaper, easier to obtain and they’re sexy, people want them don't they. I've had several quite elderly clients who've never accessed tech before but have got tablets since their event because they've been persuaded that this is a useful thing and they've really bought into it, so personally, I'd go for the tablet route.” (Speech-language pathologist 3, Focus group 1).*

Affordability relates to socioeconomic situation in Consideration 1M in the *‘First priority domain: Condition’* section.

##### Consideration 2G - Reliability

Another reason smartphones may be nonpreferred is their varied reliability;

*“I'm working with clients [for whom] either laptops, computers, [or] tablets tend to work better than smartphones. I would say [for] smartphones, I feel it depends a lot on the type of phone, depends on the browser they're using on the smartphone. I just find them a little bit more prone to glitches than tablets and laptops.” (Speech-language pathologist 1, Focus group 6).*

Indeed, a person with ABI noted they preferred not to access digital health on a smartphone because “I've got a crappy phone.” (Person with living experience of ABI 10, Focus Group 3).

###### Strategy 2.1 – Ensure responsive web design

To meet these varied considerations,

- *“It's really important that it's accessible across all devices. That's the annoying answer, but I would capitalize on all of those devices” (Speech-language pathologist 4, Focus group 1).*
- *“ensuring the technology or platform in use is user friendly is the major consideration with this [Toolkit], that it works on a variety of devices and platforms and browsers. It can quite often be a challenge with some technology platforms, ensuring that it’s compatible with a wide range of devices and internet browsers.” (Digital health implementation leader 8).*

The ability to access an intervention on any device type via the internet gave users more autonomy over their learning and engagement, with many noting they owned all device types, but had varied learning preferences. For example;

*“[it is good to access it on my] phone, because that way I can do it anywhere, but computer’s good because if you’ve got time, you can sit down and take notes. But I'm happy either way. I'd like to have it available on every platform.” (Person with living experience of ABI 6, Focus Group 5 and 6).*

The ability to access interventions via the internet on any device also enabled devices to serve as backups when troubleshooting; “For example, like today, my laptop's having problems so we're on [Person with living experience of ABI 6, Focus Group 5 and 6]'s smartphone.” (Communication partner 5, Focus group 6).

This strategy therefore also relates to the strategy to ensure tools were available on mainstream devices (1.17) in the *‘First priority domain: Condition’* section.

#### Knowledge made visible by the technology

##### Consideration 2H - Interaction with clinicians

A potentially important consideration for a communication-focused intervention is the establishment of a therapeutic relationship over the internet. An expert in digital health implementation noted that “establishing that rapport with the healthcare provider, that relationship is often vital, and I think that can sometimes be challenging in a virtual environment.” (Digital health implementation leader 8). A person with experience of both ABI and face-to-face communication partner training (Person with living experience of ABI 1, Focus Group 2) noted in their prioritization interview that telehealth delivery may inhibit the authenticity of communication with clinicians;

*“online you might be on your best behavior and you do all the other stuff before you go online; that could be a difficulty or downside. And I was impressed that [in one of our face-to-face sessions] there was this honesty that ordinarily wouldn’t have happened in front of an outsider, and technology might break that and put up a barrier and make someone feel like an outsider, like you’re on or you're not. That's just a possibility”.*

##### Consideration 2I - Interaction between communication partners

In a communication partner training program, data on the verbal and nonverbal interactions between a person with ABI and their communication partner is essential. Capturing this information by recording and uploading videos of conversations was identified by a close other as potentially more naturalistic than low-tech data collection methods;

*“you just said if you go out to the café or go to the shops [and record yourself practicing, that counts as completing your homework], that can make it easier because just letting people know that that can be getting your homework done. So, “just record that conversation, it doesn't have to be” – I remember when [Spouse with ABI] was doing speech therapy here at home, his therapist would want us to have a conversation and for me to write down what we talked about. He had these sheets and things to fill in. [Spouse with ABI] just felt ridiculous. He felt like it wasn't very natural.” (Communication partner 4, Focus group 7).*

This data collection requirement therefore also relates to expectation of people with ABI to provide patient-reported measures (Consideration 2J) and for their close others to participate in the intervention (Consideration 4H) in the *‘Fourth priority domain: Adopters’* section.

In addition to the need for video capabilities for telehealth calls, a clinician noted that a communication partner training program needed the larger vista of a computer or tablet, at a sufficient distance from the user, in order to observe a communication dyad as a whole, including nonverbal exchanges;

*“just making observations about non-verbal communications, so how the couple are sitting, whether they’re facing each other, looking at gestures and things like that. Often with smartphones you can see the person's face but you're not getting much else apart from that. So, from a clinician's perspective, all of that other non-verbal stuff is really important and good to observe, which you don't always get with the smartphone [with] just people often holding it up.” (Speech-language pathologist 1, Focus group 6).*

A larger view also allowed clinicians to gain important information about the communication environment during the interaction;

*“if you're working with a client and their communication partner, you kind of like to see them both at once and where they're sitting and what's behind them and get a sense of the environment as well, whereas I feel like on smartphones, it’s often very close up on one person, rather than getting a sense of the whole interaction at once. So, it can be a bit harder, I feel, for the clinician to see as well on a smartphone versus a tablet or laptop.” (Speech-language pathologist 1, Focus group 6).*

This data collection requirement therefore relates directly to the device screen size (Consideration 2B).

###### Strategy 2.2 – Recommend conducting telehealth video calls via tablet or computer

From a developer perspective, a clinician’s need to view both members of a communication dyad in relation to each other is a potential design consideration. Clinicians could also recommend that dyads use a tablet or computer, and this could be noted in a setup checklist (Strategy 4.2) as described in the *‘Fourth priority domain: Adopters’* section. Camera (Consideration 2B) and screen size (Consideration 2A) were discussed as key features of technologies to deliver the intervention.

##### Consideration 2J - Patient-reported measures

Reflecting broader trends in healthcare, individuals experienced in implementation discussed a need to capture patient-reported measures, noting that such data may need to be directly entered by participants themselves;

*“there's a lot of patient-reported measures now around where part of the care for patients with chronic illnesses is actually reporting their own metrics over time. We have to consider the fact that on the one level, we need the patients to be able to enter that information easily” (Digital health implementation leader 5).*

Developers may therefore need to follow Strategy 1.6 to offer multimodal input options, as described in the *‘First priority domain: Condition’* section. Stakeholder perspectives on the type of outcome measures that should be collected from the patient perspective (Strategy 3.1) are also described in the ‘*Third priority domain: Value proposition’* section.

##### Consideration 2K - Progress data

Individuals experienced in digital health implementation also discussed a need for:

*“adaptive and data driven [modules], because if you start to integrate this in, then it means different people will get different education pieces based on what they have. […] or start to automate that drawing out of EMR [electronic medical records] […] Ideally, you want an adaptive program that responds to how the person is moving through it, how they're progressing through their disease, what their disease is. This is where technology can come in.” (Digital health implementation leader 4).*

###### Strategy 2.3 – Provide a clinician dashboard

Even highly automated or gamified interventions can benefit from clinician-oriented dashboards tracking progress;

*“[The intervention] should actually be something interactive like a game more where people do something, and the backend system is actually able to track their progress. It knows that they're improving on their speech capabilities. It knows that today they said 5 words wrong. Tomorrow, it's 4 words, et cetera. That report should be in a dashboard presented to the clinician.” (Digital health implementation leader 7).*

A clinician dashboard was necessary because;

*“the practitioner needs to then see what progress they've made and needs to be able to talk with them about their progress and needs to be able to then influence the program to be targeting at the next problem.” (Digital health implementation leader 7).*

Patient-reported input into this dashboard (Consideration 2J) and the usability experience of clinicians and people with ABI and their close others may need to be tested as a user pathway (Strategy 4.1).

#### Knowledge and support required to use technology

##### Consideration 2L - Digital literacy

It was suggested that the digital literacy of a user may be a fundamental screening criterion for intervention participation;

- *“I can't be telling you how to use a mouse. That might be a trigger for [clinicians] to go, this is not an appropriate intervention for this patient.” (Digital health implementation leader 5).*
- *“for example I see with one of my clients, […]. [At] First, he didn't understand, [nor the] second, third [time]. Now he does it himself without any problem. […] with practice he gets used to it, so I think it's not that hard because it's kind of automatic afterwards. But of course, that's one client. For example, the other one, the one with the mouse, for example, how long we tried, we cannot get that mouse moving, so maybe she needs either more practice, more help, or maybe it will not be possible with her at all.” (Speech-language pathologist 5, Focus group 1).*

It was noted that the post-injury proficiency of a person with ABI in using a web-based intervention,

*“depends on the person’s tech ability before their accident, what they can draw on. There are a lot of people who don't work with technology, but then there are a lot who did before their accident. [...] and I was really surprised that all that came back. [over the course of] About a year. [...] I was really surprised that to do with work - my occupation, [...] all that technology came back, but in my personal life it was lesser. I used to rely on it [technology], but it didn't come back in the same way." (Person with living experience of ABI 1, Focus Group 2).*

Therefore, for “two people [with different prior experience using technology], we’ve both got the same injuries and stuff like that, but we’re in a different process in the capacity to deal with computers” (Person with living experience of ABI 9, Focus Group 5). However, although prior experience can facilitate the capacity of a person with ABI to use the Toolkit (Person with living experience of ABI 10, Focus Group 3, and Communication partner 4, Focus group 7), it was also noted that for ‘digital natives’ who have grown up using technology all their lives “it's so confronting and overwhelming to be having difficulties with it.” (Speech-language pathologist 4, Focus group 1).

The digital literacy of close others who may be required to support the person with ABI may also be an uncertain assumption;

*“[for] a lot of them, potentially their parents are their carers, and they may not be computer savvy themselves, which could be a challenge. There's definitely a cohort of people who are not technically literate […] that don't need to use computers for work and just never picked it up. Some of those people are young as well. So, there is that issue that might need to be thought through that if the person themselves can't use a computer easily and then actually the carer can't either, [it] could be a problem.” (Digital health implementation leader 5).*

Inversely, people with ABI may find the digital literacy of close others to be an asset;

*“my best friend, he’s tech-savvy so he'd get this straight up.” (Person with living experience of ABI 3, Focus Group 2).*

##### Consideration 2M - Unfamiliar processes

Clinicians reported that even users with a base level of digital literacy would need support to learn to use a novel program, including clinicians;

- *“everyone needs training at the beginning. I don't think anyone can do it just like that. Even the SLPs [speech-language pathologists]. Everything. Anyone needs training for something like that.” (Speech-language pathologist 5, Focus group 1).*
- *“clinicians too! We can get stuck with what we're looking at and if it's not immediately intuitive, we won't know what we're doing either.” (Speech-language pathologist 2, Focus group 4).*

It was noted that there could be a particular lack of familiarity with the process of uploading recordings;

- *“I never use something like this to be honest, yeah, uploading. Yeah, and I believe with some family, it depends on the family too, if they will be familiar with the technology, and will they have the capability to do that one or not.” (Communication partner 6, Focus group 6).*
- *“just pressing a pop up is that hard for them [so] uploading a video, I don’t think my clients can do that.” (Speech-language pathologist 5, Focus group 1).*

It was suggested that training may need to be tailored to different user groups;

*“training for therapists, different training for companies and different trainings for clients - are you planning that or will it, for example, have different interfaces? Like if your client registers through here, if your SLP [speech-language pathologist] registers through here and then everyone sees the different kind of training for themselves.” (Speech-language pathologist 5, Focus group 1).*

It was also possible that the use of accessibility options may be unfamiliar;

*“I didn’t know how to do voice-to-text.” (Person with living experience of ABI 6, Focus Group 5 and 6).*

##### Consideration 2N – Troubleshooting skill

Additionally, it was acknowledged that;

*“there's probably a tech support element to these models of care from the clinician's end. [There is a] Good chance that the first point of call that will be made from the patient will be the person that referred them to the application.” (Digital health implementation leader 5).*

###### Strategy 2.4 – Provide an introductory software tutorial for clinicians

A leader in digital health implementation noted that their platform “is relatively simple and we still run webinars for onboarding”, with anything more complex requiring training;

*“If it's complicated to set up and you give them a free 2-hour introductory session and set it up with them, then that's fine. If you just leave it to them to do it on their own, then you're not going to get many people to set it up […]. Depends on how you deal with the complexities of getting started. [….] If it’s as simple as them going to a website, signing up and being able to use it immediately and you've got an online guide that tells them what to do, then maybe you don't need training. If it's any more than that, if they need to call you up and you need to talk with them to set it up for them, then you need to work with them to get all of their patients online onto the program. So, they need to import or export all their patients from their practice management software, import them into the system, blah, blah, blah, blah, blah, there’s another 20 steps involved in training them how to create the program for each one of the patients, et cetera, then yes, you absolutely need a workshop. Depends on how simple you make the onboarding process.” (Digital health implementation leader 7).*

Clinician-specific support may need to specifically address troubleshooting the program;

- *“I would need training of course though to see also about troubleshooting. How can I fix problems, especially for that I would need it, training.” (Speech-language pathologist 5, Focus group 1).*
- *“That may mean that there will need to be some basic trouble shooting education for that.” (Digital health implementation leader 5).*
- *“Might be quite short, mightn’t it – [troubleshooting training] doesn't need to be massive.” (Speech-language pathologist 3, Focus group 1).*

###### Strategy 2.5 – Provide hardware setup support for people with ABI

It was noted that people with ABI may benefit from support in the initial setup of hardware devices; “Yes, just someone to help set up the computer.” (Person with living experience of ABI 5, Focus Group 3). This support might be provided by clinicians;

*“I turn up at their house, PPE’ed up [wearing Personal Protective Equipment during COVID-19 pandemic], like, ‘Right, we're going to set up your computer so that we can all talk to you over the next however long this is going to be’, and I set everything up” (Speech-language pathologist 5, Focus group 1).*

This setup could be supported by a setup checklist (Strategy 4.2) described in the ‘Fourth priority domain: Adopters’ section.

###### Strategy 2.6 – Provide an introductory software tutorial for people with ABI and their communication partners

Support should be provided to close others;

*“The other thing we work on a little bit is with the carer, say, is the technology training, even to access the videos. If they've never accessed a video before, taking that time to increase their technology literacy, making that process as simple as [possible].” (Digital health implementation leader 9).*

Support was especially desired in the initial stages, with greater independence after initial assistance;

- *“I'd need some help and support [initially] but as soon as I got it up and rolling, I think I'd be OK to do it by myself.” (Person with living experience of ABI 5, Focus Group 3).*
- *“Maybe in the first few weeks you can talk to someone, Skype with someone, whatever, that can actually take you through the program while you're actually getting used to it. So, lots of support at the start.” (Communication partner 9, Focus Group 3).*
- *“maybe this is a huge generalization, but I feel like most people if I - most family members, and supports - if I showed them on the computer in the hospital, kind of how it works, I think a lot of them could go home and replicate it. […] so, I think there's a lot in the group of ‘difficulty setting up but okay once they’ve seen it once or twice’.” (Speech-language pathologist 4, Focus group 1).*

###### Strategy 2.7 - Provide video guides for people with ABI and their communication partners

In particular, people with ABI and their close others expressed a desire for visual guidance to use the technology;

*“step-by-step with screenshot. Let's say, you can say in the number 1 [step]; ‘Click here and file open’. But people with brain injury [would wonder], "what [is the meaning of] the [phrase] file open?" - you need to have another picture over there. This is a where the file is, this is, click this one” (Communication partner 6, Focus group 6).*

Close others also desired a demonstration;

*“if we could have a demonstration of how to apply that so we can use it and show to, or work with, our son.” (Communication partner 7, Focus Group 2).*

In particular, video presentation of this training was desired;

- *“I'm a huge fan of a video.” (Person with living experience of ABI 6, Focus Group 5 and 6).*
- *“the video is probably good if you're if people are accessing the Social Brain Toolkit without the support of a clinician, I think like a video's kind of a nice combination of modalities.” (Speech-language pathologist 1, Focus group 6).*

#### Supply model

##### Consideration 2O - Complexity of technological requirements

A leader in digital health implementation described the complexity in the technology domain as follows:

*“it’s absolutely huge, and it's incredibly expensive and complicated. If you underestimate it, this will be the end of your project.” (Digital health implementation leader 1).*

###### Strategy 2.8 - Tailor supply models to the technological complexity of each tool

Therefore, from the development perspective, it was noted that it may be necessary to select a technology supply model that was commensurate with the complexity of the intervention requirements;

*“When you go to speak to software developers, they’ll often assure you that they can do it, that there's off-the-shelf plug-in solutions which they can use to integrate the audio, the video, the recording, uploading and so on. Invariably, things are 3 or 4 times more complex, and 3 or 4 times more expensive. You're then relying on things like browsers, external software, which you have to ensure you can continue to update.” (Digital health implementation leader 1).*

### Third priority domain: Value proposition

#### Demand-side value

##### Consideration 3A – Improving communication after ABI

For people with ABI, a fundamental demand-side value of the Toolkit was its potential to improve communication, given social rejection in the community was “maybe their loss but also my loss too.” (Person with living experience of ABI 5, Focus Group 3). For people with ABI, the potential benefit of improved communication was to improve relationships, social connection and confidence;

- *“Confidence's probably huge actually, and the other thing is connection […] I'm around people all day, but you still feel lonely. So, the ability to connect with people.” (Person with living experience of ABI 6, Focus Group 5 and 6).*
- *“Any support really, communication, anything with social interaction. Well, relationships.” (Person with living experience of ABI 5, Focus Group 3).*

Close others may also wish for the person with ABI to receive support for cognitive-communication difficulties:

*“from my point of view, with [Person with living experience of ABI 6, Focus Group 5 and 6], brain injury often comes with a, to say it basically, no filter. So, there can be inappropriate speech, there can be butting in. Like, if [Person with living experience of ABI 6, Focus Group 5 and 6] has an idea in his head, he'll be champing at the bit to try and wait and have his say, so is little things like that going to be included in it? Like the listening skills as well, is as important as the…yeah. And even like down to the basics of you have to wait your turn, wait till someone else is finished speaking. As eager as [Person with living experience of ABI 6, Focus Group 5 and 6] gets, he will often butt in because he's got to get his thoughts out when he thinks that one [laughs].” (Communication partner 5, Focus group 6).*

Clinicians had the complementary desire to provide evidence-based communication support to people with ABI and their communication partners:

- *“if you look at the evidence for treating cognitive-communication disorders, it's really the programs that this [convers-ABI-lity and interact-ABI-lity] is based on [TBI Express and TBIconneCT], is really the only solid evidence-based treatment that we [speech-language pathologists] have. Like, there's scattered research on other things, but it's not that good, I think. This is really the epitome of cog-comm [cognitive-communication disorder] treatment research.” (Speech-language pathologist 1, Focus group 6).*
- *“The thing that makes me really excited about this is that it's for cognitive-communication [disorders] as opposed to aphasia, or even, there's packages for dementia communication partner training that I have access to, but I'm really lacking that really cog[nitive-communication] basis. […] That's so much to teach the family member and the completely different way they have to interact with their loved ones. So, the biggest sell for me as a clinician working in this population is like "cog-comm, cog-comm!” as opposed to aphasia, so in terms of marketing, that's what got me in.” (Speech-language pathologist 4, Focus group 1).*

###### Strategy 3.1 - Generate and update evidence of benefit

Leaders in digital health implementation noted that there was an expectation of evidence supporting the interventions;

- *“the clinical validation is the patient impact. The clinical trial is the clinician impact. There's no way around this in healthcare. You have to always prove that it will have a clinical impact and that it will actually provide value to patients and clinicians.” (Digital health implementation leader 7).*
- *“are you really testing their knowledge or their skills at a certain time point after doing the MOOC [Massive Open Online Course, interact-ABI-lity and social-ABI-lity]?” (Digital health implementation leader 9).*

Leaders in digital health implementation noted that the evidence would be required to demonstrate comparable or superior outcomes compared to face-to-face care;

- *“there really needs to be an idea about what outcomes are achieved from face-to-face training and attempt to demonstrate that the online training is achieving similar or better outcomes.” (Digital health implementation leader 8).*
- *“Then you have to actually show that it is better than existing therapies.” (Digital health implementation leader 7).*

Objective and subjective supporting evidence was also desired by consumers, with a close other asking;

- *“has it been done that there's been 20 or 30 or 50 ABI people invited to participate in the course and get feedback directly from them. […] I don't know how many you'd have to have to be statistically significant, but gee, I'd like to see how they felt about it and what they thought their – the advantage of doing something like that there is you could maybe set some benchmarks for start and finish and get some concrete evidence and that sort of thing. We might even test ‘George’, for example, and see how he goes.” (Communication partner 10, Focus group 7).*
- *“you could set cognitive parameters that you could measure at the start and at the finish. I'm sure there would be things, and you could add to it, it would be really interesting too, wouldn't it, to add to it some subjective things, so you'd get extended family and ask them how their response to the person was. I think all that evidence would be vital. […] if you do it that way you'll have that evidence and there'll be objective and subjective evidence, but by the same token it makes a compelling case.” (Communication partner 10, Focus group 7).*

From the clinician perspective, the generated evidence may also specify an evidence-based dosage so that this could eventually be advocated to services:

*“the thing about selling it to paying bodies, the main issue I think is selling the level of complexity and the reason why there's time involved, there’s time input involved. Why it can't just be, ‘here’s a video.’ I think that's the key that you have to explain that it’s not a case of, ‘oh you could just watch the education videos and that's OK.’ They actually do need the clinician input and that the dyad is important and all of that. […] [The Lee Silverman Voice Treatment, LSVT] have to go on about that gold standard of evidence, and they have to go on about the fact that you can't do a bit of it and expect it to work. You have to do it as the evidence shows and I think...That's really, really key. It sells the quality of the package and what you're doing, but it works in the [United] States [of America] because they're selling it as one evidence-based package, and you know, it's not evidence based if you only do a bit of it. I think it's more difficult with... I think Australians and British therapists and European therapists are a bit more flexible...Certainly LSVT [Lee Silverman Voice Treatment] is very much more flexed in the UK [United Kingdom], […] But they've done that for good reason and it does explains the need for the intensity, but I think we have, when I was saying about the company I'm working for, not understanding the complexity of group work and how and how hard it is to transfer that to online, I think you've really got to sell those things, why it's important.” (Speech-language pathologist 3, Focus group 1).*

In this way, community demand-side value may overlap with supply-side value to health services as a ‘payer’.

###### Strategy 3.2 – Provide video stories from people with ABI

People with ABI and their close others expressed a desire for video stories of the outcome of the intervention from people with ABI themselves. They suggested that a person with ABI would be the spokesperson who most resonated with them, with video a highly accessible medium;

- *“Videos of client stories. Like seeing another survivor, you just connect, straight away. And the video’s an easy way for me.” (Person with living experience of ABI 6, Focus Group 5 and 6).*
- *“It just strikes me that people that brain-injured people often do take a lot of notice of other people who are sufferers of a brain injury. […] If we have people who have had a good experience and that can be used to reassure other people who may be interested, I think that would be a big thing.” (Communication partner 10, Focus group 7).*

Close others may need to hear stories of positive outcomes to give them hope;

*“at that time the family really, really need something to cling on for the hope, […] every day, every night during the year again, I'm sitting in the hall in the waiting for [son with ABI] [to recover], I read all the stories [of successful rehabilitation]. So, it is very important […] help us to get through it and how hard time. oh...It is very important at that time, at that time the hope is very important, because at that time, your life has been turned upside down...Your whole family life [has been] turned upside down completely: we had to sell the house, we had to move the house, we had to do all sorts of different things…[it was] Very difficult. I don't know how can we do this, but think about, maybe the video clip or something like that, in the website, in the Toolkit, somewhere, it's the result.” (Communication partner 6, Focus group 6).*

A clinician described effective examples of organizations sharing stories from the perspective of multiple stakeholders;

*“[State public health agency] have some really beautiful videos on their website, something similar would be perfect for this. It's actually videos of the person who's had a brain injury, a clinician, like all the stakeholders are in the video explaining from their perspective what the benefits are. And it's done with, like, a client story.” (Speech-language pathologist 1, Focus group 6).*

Alternatively, celebrity endorsement, such as a sportsperson with experience of ABI, could also be influential;

*“you need to have like a champion of some sort, they will need to find like an athlete or something, someone that you look at and go "oh, that guy's a legend, [I] will do whatever he says". (Person with living experience of ABI 6, Focus Group 5 and 6).*

###### Strategy 3.3 – Suggest that speech-language pathologists can refer people with ABI and their communication partners to the tools

In addition to evidence and stories, clinicians were identified as a credible referrer to an intervention;

- *“I would take their word because they are professionals, and the advice directly from them is better than Google or the Internet. That's what I think.” (Communication partner 7, Focus Group 2).*
- *“I just think from two perspectives on it. One is that therapists, organizations, medical professionals and whatnot they have, they have the standing, the understanding of what this is. They've got inside information to the benefits of this tool, why it would be useful, and I think that then has credibility to it, as opposed to misinformation, just looking online word of mouth and things. I mean, when you start dealing in opinion then that can be then weaponized to misinformation, and that's really dangerous, particularly when you're dealing with people's physical and mental health and then Social [Brain] Toolkits to assist people. Misinformation is really dangerous. So the second aspect of how I view it, is from the point of view of, this is a product that you guys have spent a lot of time and money developing and so to maintain the integrity of the product that you've got and that you're putting out there, you then need to protect it by putting it in front of the right people, so they then pass it on and put it in front of the appropriate people as well, as opposed to just throwing it online and hoping social media grabs hold of it. […] you just don't want one person’s opinion, negative or otherwise weaponized into misinformation.” (Communication partner 3, Focus group 4).*
- *“my experience is that the patients [and] the patients’ families trust the recommendation of their direct therapist more than anything. […] So, I don’t think it needs to come from [Association name] or [peak body name] or these big organizations for the patient” (Speech-language pathologist 4, Focus group 1).*
- *“I got involved in the face-to-face one because we’re in [City name]. Because [speech-language pathologist name] suggested it, it gave it an extra credibility. It's really good to have it online as well but coming through [Organization name] or [Rehabilitation facility name] or the speech therapist gives it that level of credibility. You know, I think it must come through them.” (Person with living experience of ABI 1, Focus Group 2).*
- *“Coming from a therapist or an organization gives it kind of a 5 out of 5-star rating.” (Person with living experience of ABI 1, Focus Group 2).*

This strategy is thus connected with the suggestion that speech-language pathologists can provide assessment (Strategy 1.2) in the *‘First priority domain: Condition’* section.

Clinician involvement was seen by some as important for treatment fidelity;

- *“it makes you worry if someone going to go through it by themselves are they going to get the best out of it. It might be good to have an initial clinician run through. […] I'm not a brain injury survivor, but personally I think it would benefit from having possibly an initial interaction with the therapist and then you can go on to do more on your own. [Person with living experience of ABI 6, Focus Group 5 and 6] obviously feels differently. [laughs]” (Communication partner 5, Focus group 6).*
- *“probably the hesitation with the MOOC [Massive Open Online Course – interact-ABI-lity or social-ABI-lity] is 2 hours - what's 2 hours of training going to do? […] the fidelity of the intervention is different for online versus with the speechie [speech-language pathologist]. So, saying, “hey, we've got this gold standard intervention around communication, and we can put a lot of that online”, but yeah. So, for me, if you're structuring it like a course, it's all self-directed, what else do they get with that bundle? Is it just videos? It's resources. What's included in the pack for them? If I signed up today and I get to navigate through the modules, what do I get?” (Digital health implementation leader 9).*

Addressing this may require a multi-tiered approach, with open access to some components and clinician involvement for others;

*“There’s loads of stuff on it online they can learn about it. They can go to their websites, whatever, but when they want to do it, they have to go and find a certified clinician. There's an advantage to that in that you could have a situation if it's too open and too available where people are accessing bits of it and not perhaps, I don't know. I mean, it's OK, maybe it's OK for people to access the education stuff, isn't it? I don't know. I think maybe you just need to think about what, what should be freely available and what, what needs clinicians’ input.” (Speech-language pathologist 3, Focus group 1).*

However, in the experience of leaders in digital health implementation, “If the clinicians are the limiting factor on the patient reaching the next stage or continuing to use it, then you have a big problem.” (Digital health implementation leader 3 with living experience of ABI). The need to disseminate through channels other than clinician referral is highlighted in Strategy 3.7.

###### Strategy 3.4 – Gain momentum through clinician champions

However, clinician referrals were only possible if the value proposition to clinicians was clear; “if this group doesn't see it as useful, they're not going to refer patients to it.” (Digital health implementation leader 5). A primary way the value proposition could be demonstrated to clinicians was through other clinicians; “[recommendation by a clinical colleague is credible because] I think when you present this at a conference, it's presented with stats and numbers, and it sort of makes [it] a bit more distant from real life.” (Speech-language pathologist 1, Focus group 6).

Leaders in digital health implementation particularly advocated for leveraging these early adopters;

- “*identify them early and use them to sell it for you to the others who may be a bit risk-averse or a bit reserved or not understanding the purpose and the why.” (Digital health implementation leader 6).*
- *“it’s really critical from your aspect that you find some of those people – and there’s always a few of them, so there will always be a small percentage of people that look at it and go ‘absolutely, this is a great idea, let’s do it’. Then the more it gets out there, the more people will start to hear about it and become onboard with it.” (Digital health implementation leader 8).*
- *“who you identify as you start, those people that are saying that, the clinicians, you make a list and you do provide access. So, they’re called your first lean-intos kind of thing, so they’re your biggest advocates and you can afford that you make mistakes with […] and be honest with them that you might not have everything right here. There’s going to be bugs, […] some teething problems on the tech side. Say we are really happy to give you early access and free access, so you just need [it to be] free at the beginning. You can test barriers with your beta on purchasing later, but you say that – you lean into it, [make the jump], but not to everyone, people that are really, really keen for their patients to do it and you just make their – they become your alphas, so that’s how you identify them. They’re the passion people.” (Digital health implementation leader 3 with living experience of ABI).*

As a practical example,

*“you can give them the codes, so you might have cards, you might have a pamphlet and you say, ‘we’re very excited to have you included in our alpha trial’ or – people quite like a password. Barriers are not always bad……so it makes it exclusive. It makes it available to them if they’ve been at the conference. You say they can pass it on as well. You can have a landing web page, you can have something like ‘contact us if you want to be part of the alpha trial’.” (Digital health implementation leader 3 with living experience of ABI).*

Eventually, this could lead to the positioning of an intervention as a default treatment option;

- *“over time, if everybody is using the Social Brain [Toolkit], if every single speech-language therapist in Australia is using it, then it's not a risk, because consumers will expect it, and therapists who work for those organizations will expect it. When we launched [digital health service], there was absolutely no brand recognition, […] but those GPs [General Practitioners] who have used [digital health service] to refer patients to, continued to do so, and we don’t promote [digital health service] to those GPs, but they’ve actually embedded our referrals as part of their standard practice.” (Digital health implementation leader 1).*
- *“[App name] cost hundreds of dollars to get. But for most speechies [speech-language pathologists] it's like this weird like no-brainer... ‘Of course, I'm getting. It is [App name]. We have to get it. We have to have it’ […] it's like [practice area] is [App name]. There's no other thing, […] We as speech path[ologists]s know that it's not always the best option. Sometimes it's bloody beautiful and fantastic, but other times it's really not what you want, but outside of the speech path[ology] community, it's synonymous with [practice area], that's what [practice area] is: [App name]” (Speech-language pathologist 4, Focus group 1).*

##### Consideration 3B - Supporting communication partners after ABI

People with ABI wanted communication partner training to support their close others;

- *“it would have a lot to do with giving the friends and family a warning of what to expect. [In the] Early days when you're experiencing the brain injury, I think I was a very different person to how I used to be, so it would be nice to give friends, families and people were coming to see you a bit of a warning to let them know what to expect when you meet someone who has just experienced a brain injury, so it would be very valuable information for the people who aren't expecting it, 'cause otherwise they came across as quite a shock. […] [it could help them to] not be too shocked or react in a negative way. In doing so, it could actually impair the progress of the healing, 'cause if they start screaming; “Oh no, this crap. Oh no, this-“, that could really affect the participant who's going through the recovery in a negative [way]. It would be immensely helpful to avoid any negative or backward progress or regression.” (Person with living experience of ABI 3, Focus Group 2).*
- *“sometimes if you haven't done a degree like Social Work, some of the people who have just got like a Cert[ificate] 3 or support workers, they can be ignorant or just unaware of some of the needs of a person with a brain injury. So, I think the Tool could help them, and also sometimes just friends and family, because they can do or say things because they're unaware of our difficulty or challenges.” (Person with living experience of ABI 2, Focus Group 3).*

The need was confirmed by a spouse of a person with ABI. For families;

*“a lot of family and friends could get a lot out of this as well, not just the injured person. It goes back to what I was saying before about extended family members also feeling judged, like they're not doing it properly; ‘I don't know what to say when I don't understand what he says to me’, or that sort of thing. I know I personally feel like I have to keep the conversation going when certain people come to visit. That person says they're coming to see [Spouse with ABI], but if I leave the room it's all a bit chaotic, because the person doesn't I guess know how to engage with him in those conversations like they used to have. So, them being trained I guess a little bit as well would be - they don't necessarily have to go through the course with him [as in convers-ABI-lity] but they could watch [interact-ABI-lity], that could be really helpful. Then everyone's going to succeed, everyone's going to feel like they're achieving something in that situation, which would be fantastic for all the people that have been traumatized by what's happened.” (Communication partner 4, Focus group 7).*

However, the need extended to other communication partners;

*“it would be a bit like a ‘train the trainer’ situation, and I know in our case when [Spouse with ABI] first came home from hospital, we had support workers coming in to support him in the home, because physically he was not as independent as he is now. […] Would there be scope for training multiple support people? That's I guess my question. So, if you do have a partner, like in our situation, but you also have support workers, what would be the scope for… and you might be in a situation where you don't have the same support worker that's coming in every day. These are people who have got quite severe physical - [Spouse with ABI] wasn't able to get out of bed himself, he wasn't able to dress himself, he wasn't able to shower himself. So, we needed people. Then he was having his therapists coming to the house, so we had speech therapy, occupational therapy, physiotherapy. I wasn't working at the time but there would be situations where families where the non-injured person is at work fulltime. I just question whether the continuity would be there if different support people are having to be supported as well.” (Communication partner 4, Focus group 7).*

Clinicians corroborated this need to support close others, including multiple paid support workers, particularly through an educational intervention such as interact-ABI-lity:

- *“there's a group of people that I haven't thought about until now who would really, really benefit from this, but perhaps the slightly adjusted version [interact-ABI-lity]. So, I have many, many patients who discharge from hospital into supported accommodation with paid carers and my responsibility is to train those carers, so it's a group of carers that have never met this person before, have most often never ever met a person with a brain injury before, but are, as of tomorrow, going to be the full-time carer for this person with brain injury, and I get given an hour to teach them everything about cognition and communication and swallowing before tomorrow. And I always feel, naturally, that I just like, skim the surface and go, ‘well, I know you're going to have all these issues, but I don't have the time, we haven't been allowed the time, or funding or whatever to properly give you the skills to manage’; I would love being able to give this package to these carers who have never met someone with a brain injury and go, ‘these are some of the things you can encounter and these are some of the ways you can respond’. So, it would be slightly different in that they wouldn't perhaps do the, kind of uploaded videos and things, [it] would be more of that classic, online learning education rather than the dyad, but that could be like a spinoff version that I would capitalize on like nobody’s business! [interact-ABI-lity is for unfamiliar communication partners that have just met someone to do an online course to learn what brain injury does to your communication and how to respond.] That is the best thing I've ever heard.” (Speech-language pathologist 4, Focus group 1).*
- *“it’s those CC, those cog-comm [cognitive-communication disorder] clients, where there's an effect that you're seeing ripping out on the family, and it's that aspect of the Toolkit, the education side of it, I think would be really, really good for those.” (Speech-language pathologist 3, Focus group 1).*
- *“I'm a part owner and operator in a home care NDIS [National Disability Insurance Scheme] provider. So, we have a lot of staff, a couple of hundred staff that would possibly use a tool like this to educate themselves further.” (Communication partner 2, Focus Group 3).*

Close others could also be empowered with information;

- *“I really like the approach. I think that it’s really valuable. It’s something that I know my family went out to look for if something like this existed […] you’re empowering the whole family unit and the carers around them. It’s such a positive thing to be doing and it’s something that we need. […] I think that it’s going to be really positively [accepted by clinicians] as well, because they’ve got issues with families not knowing enough and they talk to the patient the most. You have the possibility to change the whole unit and the way that it responds to the person, therefore making that journey easier for that person. So, you’re seeing the person as a whole and part of a community, not just treating a deficit.” (Digital health implementation leader 3 with living experience of ABI).*
- *“at that point in time we have no idea what we're going to do, what are we going to be faced with? Something like [the Toolkit], it will help people in [the] first place. I think we kind of [need] to set up something to help people in the first few weeks when they started to leave hospital to rehab. It's very important because nobody knows what's going on.” (Communication partner 6, Focus group 6).*

Close others could also have positive experiences of shared success:

- *“one of the satisfying things about doing stuff with [Person with living experience of ABI 1, Focus Group 2]; that there are always these changes, even though some of them may have been subtle. But that was a reward, friendship was rewarded. Yeah, and so being able to maintain that, and I suppose when a friend does experience such a trauma to hang onto them, and to watch them continue to grow and come back to themselves is, yeah, it's...a pretty amazing reward actually.” (Communication partner 1, Focus Group 2)*
- *“I think that the person looking after the person with the brain injury would gain a great deal from the courses as well. I think there can be a sharing of success there too” (Communication partner 10, Focus group 7).*
- *“enabling that carer to improve, be part of that journey, is brilliant.” (Digital health implementation leader 9).*

This therefore relates to the provision of positive feedback of progress (Strategy 4.5) in the ‘Fourth priority domain: Adopters’.

###### Strategy 3.5 – Ensure that communication partner training is described in an empowering rather than judgmental manner

Close others of people with ABI noted that, when communicating the benefits of communication partner training, it was important to avoid connotations of ‘passing’ or ‘failing’ as a communication partner;

- *“any of these programs and things, which are brilliant, it would be helpful if it could be presented in a way that the people who are going to be living the therapy every day with the brain-injured person also don't feel judged or that it's a test. Not a test, it's not a test in any way. Yeah, so that's been my struggle with those things.” (Communication partner 4, Focus group 7).*
- *“course” has been used quite a lot that “we’re doing this course” and that sort of thing leads towards classroom [connotations], and I just wonder about just how it's packaged from an engagement point of view.” (Communication partner 3, Focus group 4).*

Instead;

*“the word “tool” is of real importance, that this is a way of developing tools and a set of skills in which to help a person with a brain injury with their recovery, whatever that looks like, this is just one more arrow in your quiver, to give you confidence, to give you some level of ability to be as helpful as possible. So, I think that it gets sold as ‘we are helping you to develop tools to help a person with a brain injury’, or for a person with a brain injury, to aid in their recovery. It should be sold in the same fashion that you would sell a scalpel to a surgeon. You would sell a textbook to a student, and things. These are tools of your trade, and this is how we can help you to develop these skills.” (Communication partner 3, Focus group 4)*

This related to the need for close others to manage performance anxiety in the *‘Fourth priority domain: Adoption’* section (Consideration 4D).

##### Consideration 3C - Addressing social media use after ABI

Close others may want a social media intervention for the person with ABI:

*“[Facebook is] good for him to stay connected so I think it is helpful for people, but he also needs to learn, and I imagine other brain-injured people too need to learn the pitfalls and the mistakes and the ‘don't put your address on Facebook’ and ‘don't post photos that have got anything personal in the background’, and ‘if you're going to post a photo of someone's child you've got to ask their permission’, and all these sorts of things.” (Communication partner 4, Focus group 7).*

This could be important given limitations in a close other’s capacity to provide this support;

*“[social media training] would be helpful because he didn't know about it before [his injury] so it's a new thing. His brain has to learn how Facebook operates, and I don't always have the time to sit there with him and show him. I'm relatively in the world new to Facebook myself.” (Communication partner 4, Focus group 7).*

Clinicians may also need guidance to support people with ABI with their communication on social media;

*“Possibly for my age group, mid-40s, social media is a bit of a new thing to us and so it would just rather not address that. So, I think that is a really big selling point of the Social Brain Toolkit that you are heading it head on; ‘Let's see what to talk about with this, let's support people with it’.” (Speech-language pathologist 3, Focus group 1).*

There could therefore be a disparity between clinician familiarity with social media and the need to provide social media support as part of neurorehabilitation, with a clinician noting “part of my role is to support them to access social media again and kind of re-learn how to use their phone or computer or iPad”, and;

*“I've had the social media issues slap me in the face. We had a patient who gave their credit card details on Facebook marketplace and had thousands of dollars taken. So, I can't avoid this social media problem.” (Speech-language pathologist 4, Focus group 1).*

###### Strategy 3.6 – Create clear, simple messages to explain and differentiate between the 3 different tools in the Social Brain Toolkit

Given the Toolkit contains 3 different tools with different purposes, it was considered important to ensure clinicians “are really clear about the different arms of it” (Speech-language pathologist 2, Focus group 4). An expert in digital health implementation noted the value of;

*“simple messaging, 3-word slogans, […] good quality accurate but simple messaging for those groups. […] consistently hammering home that simple message, which is in this particular case it might be simple; ‘tools to improve …’.” (Digital health implementation leader 5).*

In particular, a clinician familiar with TBI Express and TBIconneCT noted “conversation skills training” may be a more accurate definition of convers-ABI-lity than “communication partner training” to differentiate it from interact-ABI-lity;

*“for clinicians it makes much more sense for interact-ABI-lity to be the [communication] partner education bit. convers-ABI-lity, if you were to market that as ‘this is communication partner training’, I think many clinicians will go, ‘I don't have time to do communication partner training’. Whereas, they need to understand it's not just communication partner training, because you can work on the skills of the person with brain injury in that program as well. So, I think if it was marketed as ‘conversation skills training’, where you can improve the skills of the communication partner and the person with brain injury, I think clinicians are much more likely to take it up.” (Speech-language pathologist 1, Focus group 6).*

A close other and person with ABI in that focus group strongly agreed that this phrasing was also clearer for them to understand.

##### Consideration 3D – Improving access to support

In addition to its intended benefit to support communication, people with ABI and their close others identified the potential for the Social Brain Toolkit to address current gaps in the continuum of care;

- *“just 'cause of the whole jumping through hoops just to access the NDIS [Australia’s National Disability Insurance Scheme] support and I think I needed it the most when I was just out of hospital and there was like months and months and months before I could actually access anything.” (Person with living experience of ABI 2, Focus Group 3).*
- *“as I understand it, the research shows that a lot of the gains you can make are in the first 12, 18, 24 months post injury and we got really good [support] for the first of 6 months with hospital outpatient [services], but then eventually you reach the limit of that and then we had a big gap there and something like this would have been useful bridging that gap. It would have been good if we had other options in between.” (Communication partner 2, Focus Group 3).*
- *“filling the gap between [hospital and rehabilitation]. I think it sounds like a lot of people felt like there was a gap before or after getting any sort of rehab. For me, that was huge because I'd just found it incredibly frustrating knowing that that was a critical time to be doing rehab, and I wasn't doing anything. So that would be the standout for me” (Person with living experience of ABI 10, Focus Group 3).*

This need relates to considerations of the ‘Stage of Recovery’ (Consideration 1A) in the ‘*First priority domain: Condition’* section, and *‘Scalability’* (Consideration 3H).

The availability of an intervention via the internet may also enable individuals to access communication support who may not otherwise be connected to services at all;

- *“Ideally, someone has a therapist in their life or they're a part of an allied health service or something, but those that are not, that do have social anxiety, that are agoraphobic, whose friends have dropped away, friends and family have found it too hard, and they do sit at home on their computer because that is a way they can connect with the world. They have just as much right as someone who doesn't have all those services to be able to access ways to help themselves.” (Communication partner 4, Focus group 7).*
- *“if this is something people can access without having to have a clinician attached, that's important, as well as if they do have access, that's great, but if they don't, that means you reach a lot of people. And its timings too, like, people might have had access to a clinician at one point, but that wasn't the right time or whatever, so, it increases the chances for them to access [communication intervention] when they're ready.” (Speech-language pathologist 2, Focus group 4).*

This expressed need to address current service limitations was corroborated by clinicians;

- *“clinicians are always looking for very handy tools! They're always looking for a way to help make sure that they can serve as many people as well as possible and always keenly aware when there's people waiting and not getting any service, so if there's a way to support people to get a service sooner when you know personally you can’t do it, that's good. And then when you are actually working with people, then it gives you a way to serve some people who wouldn’t be able to access your service otherwise, from a remote kind of access point of view. I think clinicians would be interested. I'm not too sure there's going to have to be a hard sell from that point of view.” (Speech-language pathologist 2, Focus group 4).*
- *“what I like about this is that it's an adjunct to your therapy input […] it's propping up this gap I have, where I can't get the family to come in, or they keep reporting the same issues and I feel like I've gone through it before, but it's not sinking in for whatever reason, so let’s present [it] in a different manner and you can refer back and have a practice and stuff.” (Speech-language pathologist 4, Focus group 1).*
- *“in a rehabilitation center, [the] speech therapist goes there once a week and sees all the clients right? But what about the rest of the week? Or clients cannot make it that day? Then you can sell it to this rehabilitation center. […] there's an SLP [speech-language pathologist], but for the rest of the week you don't have an SLP. Here is our app. That's how we can approach rehabilitation centers who don't have an SLP on location.” (Speech-language pathologist 5, Focus group 1).*

###### Strategy 3.7 – Disseminate the Social Toolkit through as many channels as possible

To reach underserviced groups, stakeholders recommended dissemination through multiple channels;

- *“is it better to advertise it online through [Organization name], through your clinician? I think all of the above. For different audiences, I think that different audiences need different things.” (Speech-language pathologist 3, Focus group 1).*
- *“the more options out there, the more people will hear about it.” (Communication partner 5, Focus group 6).*
- *“It should come from as many people as possible, as many avenues, from everywhere possible, so everyone might have a glimpse of knowing about it.” (Communication partner 9, Focus Group 3).*

This included distribution via peak bodies and organizations; *“you might find that [Peak body] or [Organization name] or groups like that may be good [at] passing the word around.” (Speech-language pathologist 2, Focus group 4).*

It was also possible to distribute an intervention through word-of-mouth both in-person and over the internet;

- *“I'm thinking ‘how else clients can communicate with each other?’. It's only those therapy groups, communication groups and in rehabilitation centers if they talk [to] one another, with their neighbors et cetera. You can have a Facebook group or something like that.” (Speech-language pathologist 5, Focus group 1).*
- *“It’s better if something as part of the app itself, either after the first module or something, just ask the question, is there anyone you know that you think would benefit from this? And then email them to push something to them. After someone has seen the value of it.” (Communication partner 2, Focus Group 3).*

It was also necessary to have a website;

*“having an online presence so people can evaluate it and as a consumer, a potential consumer, if I'm worried about something I don't necessarily get occupied by it between 9:00 and 5:00. I might think about it at 2am and it's, you know, my best mate and I want to do something. If it's 2am, I will probably sit up and have a look at a search and that's where I think sometimes that online is useful, as well.” (Communication partner 1, Focus Group 2).*

At present, there was a gap between a desire for clinician referral to programs and clinician awareness of service options (Strategy 3.6);

- *“we went through the private sector and they were really uninformed. […] we're just highlighting that sometimes the private sector ones don't seem to be the attention for programs like this. [...] you've got a bunch of private hospitals that still have a lot of patients coming through that this would be applicable to.” (Communication partner 2, Focus Group 3).*
- *“It was only through our research that we heard about it. If medical professionals could be more informed of all the support that is out there and available would be helpful.” (Person with living experience of ABI 2, Focus Group 3).*

Therefore, clinicians should not be the only way individuals could access the program;

- *“If it's primarily clinicians [rolling it out], I think people would find it harder to access.” (Communication partner 5, Focus group 6).*
- *“If you’re using the service to reach the people and their effectiveness to roll it out, it will limit the accessibility of how you reach your intended market, whereas – but people will find you online anyway other ways. But if that’s the main way, that will be a limiting factor.” (Digital health implementation leader 3 with living experience of ABI).*

To determine which channel is most effective,

*“You could also ask how you heard about it. That would give you feedback on how people got in touch with it, who's referring and who is not...” (Person with clinical and living experience of ABI 8, Focus Group 3).*

#### Supply-side value

##### Consideration 3E - Improving service efficiency

A web-based service delivery model offered potential to improve service efficiency;

- *“that is another selling point that this is another way to access it away from the traditional. And that's why TBIconneCT is also interesting people, because people are looking for different ways to do this model of...less arduous ways to do it. I'm playing around with it myself to try and work out how I can do stuff online, and that was one of the reasons that attracted me to the whole Social Brain Toolkit, well part of it, was like, “Oh great! Someone’s already doing it!” (Speech-language pathologist 3, Focus group 1).*
- *“on the health service side, or the system side – delivering virtual care can certainly improve efficiency. In terms of what we’re trying to achieve here, I would really see it as an improver of quality.” (Digital health implementation leader 8).*

Carers confirmed that web-based access was desirable to reduce the burden of healthcare access:

- *“If we could have the online [Toolkit], there's less travelling and there will be less cost involved. For my son, traveling is a big problem because we need to hire a taxi with wheelchair access and for a one-hour session it may take us a whole day with a lot of money to pay.” (Communication partner 7, Focus Group 2).*
- *“online video call appointments would be wonderful.” (Communication partner 7, Focus Group 2).*

People with ABI also considered web-based access more convenient;

- *“it's a lot easier than setting up an appointment with your speech pathologist at the location. Having it digitally is much, much easier to do.” (Person with living experience of ABI 3, Focus Group 2).*
- *“I know I had trouble with my daughter when I did [TBIconneCT face-to-face]. Doing the speech thing face-to-face back then was more difficult.” (Person with living experience of ABI 1, Focus Group 2).*

Web-based availability could also facilitate access for people who had difficulties accessing healthcare, including those who are rurally and remotely located:

- *“you can reach people in regional and remote areas, which is a real positive, that might not have access to a metropolitan area as easily to face-to-face interventions.” (Digital health implementation leader 9).*
- *“there's a lot of people who won't be able to get access to the hospital, a lot of the time. Maybe they're on limited means, even getting to the hospital is hard. People in lots of different situations where they don't feel comfortable, leaving the house; it really helps people connect to services.” (Communication partner 9, Focus Group 3).*

###### Strategy 3.8 – Use the language of the funder to communicate the Social Brain Toolkit’s benefits

Leaders in digital health implementation noted the benefit of tailoring communication to the strategic objectives of funders;

*“the way you communicate just has to be individualized, perhaps. You could go to one service provider and say ‘hey, this is really going to improve your efficiency’, and they’ll be 100 per cent onboard, and you could go and say that to the next one and they’ll be like, ‘I don’t care about efficiency. I want quality of care. I want patient outcomes’. So, I think understanding what a provider wants and tailoring communications to them is also important.” (Digital health implementation leader 8).*

In particular, at the time the study was conducted,

- *“there's some terms that are exciting to the system. Now, there's terms that I would be suggesting you might lean towards at the moment. […] ‘Virtual care’, ‘patient-led care’, ‘Self-service’ […] a ‘customer-centric’ model of care. […] if you start associating your product with some of these terms, which I think can be legitimately applied, it may be more attractive to senior leaders.” (Digital health implementation leader 5).*
- *“Digital therapeutics for me really just means that you've - like you said, you've got typically a face-to-face model. It's best practice. It's gold standard. Now we're taking that model, that intervention, into a digital health format, with the majority of that being self-directed learning, which allows you scale. That's the problem we're talking about. Hey, I've got 135 - 100 million people, I want to access this at scale. […] Whether you use that term digital therapeutics - it's a good term, because from a commercialization perspective, it attracts investment, if there's, like you said, a good business case for it.” (Digital health implementation leader 9).*

It could be helpful to provide clinicians with pre-set phrasing to meet funder criteria;

*“the ‘reasonable and necessary’ criteria for NDIS [Australia’s National Disability Insurance Scheme] which they use as the test when they fund things, the A to J criteria could be a good checklist for you to look at. So, when clinicians are writing reports to get funding for people to access services, we have to make sure they meet, our requests meet this A to J criteria. So, for example, it is the most cost-effective option there is. There is no other service that could do the same thing for cheaper. So, there's a bunch of criteria that could be helpful. the more you can tick off of those, then more likely the NDIS will fund it.” (Speech-language pathologist 1, Focus group 6).*

The need to target insurance schemes as a payer is part of a potential hierarchical pricing structure for the Toolkit (Consideration 3.12).

###### Strategy 3.9 – Communicate that the Social Brain Toolkit complements or adds value to existing services, rather than replacing them

Leaders in digital health implementation identified that it was extremely important to manage user and provider perceptions of digital health as compared to face-to-face care;

- *“if it's done standalone outside or in addition to normal care and not interfering with the model of care, I would imagine there would be little opposition if you can demonstrate real value and benefit.” (Digital health implementation leader 4).*
- *“don't be afraid of saying that for some people it's not a solution. If the majority of - if only half of them go online then the other half, there is room for them in the waiting room, because we cannot - we are an aging population. […] we have more and more diseases and we cannot be there, all of us. It's not possible. If I stay at home on my computer, then you can have the seat in the waiting room.” (Digital health implementation leader 2).*
- *“it can't be seen as an alternative to seeing a clinician in person, it needs to be positioned as, ‘this is actually an enhancement, it improves your access and it's actually a better quality of service than you can get from a clinician alone’. I think this is really important because people feel like we're just cheaping out. […] The actual rationale for virtual care […] is access, it's not cheaping out, it's actually providing services rurally that we couldn't before. Now, patients actually, when it comes to specialists, really like this once you start pointing out that, okay, well the alternative is you drive to [city name] and then you wait in an office for 3 hours and then you're seen, consultation's 2 to 3 minutes long and then you drive back to [rural town name] or whatever. They hate that. But virtual care gives them an alternative to that is vastly preferable. […] It's kind of about managing expectations of the public because it's not a wholesale rejection of the model but we're still finding our feet around what's acceptable. I think in regards to [the Social Brain Toolkit], it seems to me like it wouldn't be an alternative to clinician services anyway, it would be an enhancement.” (Digital health implementation leader 5).*

For example, as described from the clinician perspective,

*“that's where I feel like I have a gap in in resources to give people. I've got the personal skills to be able to support in session as a therapist, but I've got nothing to give to them, offer them to work with in their own time.” (Speech-language pathologist 4, Focus group 1).*

Other examples were discussed further in relation to the value proposition of improving access to support (Consideration 3D).

To avoid negative perceptions,

- *“whenever you introduce something like this, you have to do it very carefully and you need to work with consumer groups and others to show that this has benefit and that it's actually about improving outcomes, not substituting care or reducing care.” (Digital health implementation leader 4).*
- *“for these types of interventions to succeed, you actually need to engage with the stakeholder community. That includes whatever the associations are for speech language therapy, the various decisionmakers or people who are influential, and have them trial it, provide feedback, and potentially become advisers or supporters of it. […] but also ensuring that they aren't sabotaging it, either. […] If they, for example, weren't consulted. In the past, I’ve thought of healthcare as benevolence, as service providers, as wanting to support consumers, and working together in harmony. It's actually, obviously, commercial, competitive. People are concerned about their individual value proposition, and by extension, of that proposition, their colleagues and friends. If you're perceived as a threat, then you'll be treated as a threat. […] Another way of framing it is you're actually helping them add value to their service. That means you have to think through your commercial model as well, because your finance and funding and so on, in order to be able to continue down this path. So, ensuring that your funding model isn't competing unnecessarily with their funding model. […] [Digital health service name] is funded through, essentially community grants from the federal government. Now, because of that, we're actually not competing for funding with MBS [the Medicare Benefits Schedule] or with other ways that [face-to-face clinicians] are funded. We also work with a very large number of people, who don’t access traditional services. In fact, we refer many people to traditional services. As soon as I explain those 3 facts to, for example, the [Professional peak body names], they realize we're not a threat, but we're actually helping their business model. They may not want to promote us, but they won't criticize us, because really, they want patients treated. They want to support the community. So, they’ll tolerate us. Yeah. Now, it's quite a shift. I found it took me about a year or so to understand and be okay with that fact that it is competitive, and if you don’t accept that it's competitive, you will make naïve decisions and mistakes, and I've made many of those. That's not to say that you have to change your moral base or your standards or your values, but you just have to be careful about how you communicate and be very clear about what the value proposition is for you on engagement, and, on the flip side, what the risks are, as well. [For example,] If this program is free, then why would people want to see a speech language therapist?” (Digital health implementation leader 1).*

###### Strategy 3.10 – Demonstrate to funders that there is a financial benefit to providing the tools

Leaders in digital health implementation and close others of people with ABI acknowledged;

- *“If you try to get the funds from the government, we need to prove [to] them it is cheaper. Less money. […] Everything [is] around the money.” (Communication partner 6, Focus group 6, agreed by Communication partner 5, Focus group 6).*
- *“Money is always tight in health, but it is incredibly tight at the moment. So, that is probably the other area that you'd need to really focus on in terms of you know, this is how much it costs, these are the benefits. That kind of cost-benefit analysis of, by investing in this, how much are they saving in other ways. I think if you're able to kind of demonstrate that, you know, if for instance you could reduce outpatient appointment. You know? Or improve access and flow.” (Digital health implementation leader 6).*

There were potential financial advantages to offering web-based care;

*“[face-to-face], you’ve got costs in travel, you’ve got costs in terms of time. At a system level, it usually costs less to deliver an intervention in the community and keep them out of hospital, rather than them occupying a hospital bed.” (Digital health implementation leader 8).*

##### Consideration 3F - Providing person-centered care

Another key value proposition of the Toolkit for both healthcare systems and users was the empowerment of people with ABI and their close others within a broader ethos of person-centered care. From the health system perspective;

- *“delivering care in the community and wrapping care around the person, that is one aspect of person-centered care. The only way we can do that is by starting to deliver care in the community. Delivering care in the community often improves accessibility. […] Broadly speaking, not just specific to brain injury, going to hospitals or going to health centers, it sometimes provides a lower experience of care for people – because of the need to travel, because of the inconvenience of it, they often have strict scheduled times that they need to go. So, I think the more we can deliver care in the community and be flexible, we’re actually improving experience and accessibility.” (Digital health implementation leader 8).*
- *“anything that supports and empowers and enables consumers and their carers of healthcare and supports healthcare clinicians in delivering person-centered care is a winner.” (Digital health implementation leader 6).*

Person-centered care includes consideration of the support provided to close others (Consideration 3B).

For example, a person with ABI noted; “I'm high functioning so I would prefer to try make my way through it.” (Person with living experience of ABI 6, Focus Group 5 and 6). An expert in digital health implementation, noted that;

*“the autonomy of computer-assisted learning and therapy, that can in certain instances where people are feeling sensitive or embarrassed or challenged, then technology can support that. […] there's evidence that it can get over reluctance to seek assistance and help [among men]. […] I had a friend, actually a colleague, who was [….] really quite severely brain injured and there was a huge embarrassment level to him reengaging in society. So, I think if you get these technologies right, then you can provide a clear pathway to people […] it should be able to provide easy and discreet access to learners.” (Digital health implementation leader 4).*

This need was discussed in the ‘*First priority domain: Condition’* section regarding managing social stigma with an invisible disability (Consideration 1L) and the sociocultural aspect of healthcare access for men (Consideration 1N).

Finally, it may be noted that upfront communication of time requirements (Strategy 4.6) may be important for asynchronously or flexibly delivered healthcare options;

*“we often think people have to be available between 8am to 5pm Monday to Friday. So having considerations I suppose for people to interact outside of traditional hours […] Which the - I mean the solution sounds like it does, which is also one of the selling points. I think is that patients are able to access it at any time of the day, week, so they're not restricted by an appointment between certain hours or…” (Digital health implementation leader 6).*

###### Strategy 3.11 – Maximize user autonomy

In keeping with the ethos of person-centered care, stakeholders felt that users should be given autonomy over what to complete;

- *“They can choose their own topic, and then in each topic there is the easy way [the basic information] and the hard way [if you want extra information, you can read it yourself here]” (Communication partner 6, Focus group 6).*
- *“choosing the topic will give them ownership. Don’t tell them to do it.” (Person with living experience of ABI 6, Focus Group 5 and 6).*

This was important because;

- *“the delivery of the [Social Brain] Toolkit has to meet what the patient wants. Every patient wants to achieve different things at different points in time. I know it’s probably difficult with the Toolkit, but there needs to be some flexibility around what a patient wants to work on. For example, a patient may think, ‘Well, this is important to me, that’s what I want to work on first’ within the [Social Brain] Toolkit. So not necessarily a linear, systematic approach to completing the modules or the learning – I think there really needs to be the opportunity for the user or patient to log in and say, ‘That’s important to me, this is what I want to get to, let me do that first.’” (Digital health implementation leader 8).*
- *“something like this puts the power of information in the hands of the patient. So, it's quite empowering for the patient to have access to this.” (Digital health implementation leader 6).*

Likewise, there was a desire for;

*“capacity for me as the clinician to kind of go through and pick modules that are most relevant to the patient and family, and be like, ‘well, that's not even applicable. They don't even present with that difficulty, I’m just going to cut that part of the program out for them’. […] And it doesn't have to be that I can block access to things - if it's all optional and you can kind of just skip past the bit and then I say do it like a future carer, like ‘do Module 5 and 7, 'cause they are particularly relevant for this person, that's great.” (Speech-language pathologist 4, Focus group 1).*

People with ABI could also be empowered by options to connect to other people with ABI;

- *“you almost never meet someone within your social network that’s also had a TBI. […] TBI sufferers usually feel ostracized. I think If you can utilize the Toolkit to generate synergy amongst people and create some sort of feeling of support, that all they've gotta do is flip open their laptop and they've got a world of people with a TBI that are going through the same thing, it can center someone with a TBI […] maybe in the future if not straight away, [but] that'll be of immense utility, […] arm people with something that's going to help them get back out into the real world, but also make them feel supported, like they're not completely alone in spite of their condition.” (Person with living experience of ABI 7, Focus group 4).*
- *“The other option as well is a place where resources can be given out for support groups, and [organization name], for example in [State name], and it's really great for linking people up with support groups and things. I'm not sure if that's another way of doing it,” (Speech-language pathologist 1, Focus group 6).*

The person-centered focus of this strategy on supply-side value inherently overlaps with demand-side value.

##### Consideration 3G - Sustainability

Individuals experienced in digital health implementation attested to a need to look beyond demonstrating benefit for initial upfront investment to longer-term sustainability. Leaders in digital health implementation reported numerous interventions failed to be sustained;

- *“doing something because it’s the right thing to do doesn’t always translate into something that stays around and it’s always so sad. [Laughs].” (Digital health implementation leader 3 with living experience of ABI).*
- *“It's easy to get one-shot injections for that sort of training, it can be harder to get this sort of sustained funding required to maintain implementation.” (Digital health implementation leader 5).*
- *“This is paramount, right? This is the number one failure I think in most of these systems, is not understanding the primary economics and usage of this. So, who is going to pay for this? Who is going to implement it into their systems of care? How do you do that? I mean I've seen just so many examples of people building solutions with no home. It's absolutely vital. Obviously, a key component of that is showing benefit and impact. But that's by no means enough, right? There's a lot of [digital health] platforms as well that show benefit but they still don’t go anywhere, right? Because they're not - people don’t understand that health care is a business, so who is going to fund this? […] It's often a failure in academic programs, that they build stuff and then they think they can go to the funder at the end. I've just seen it so many times.” (Digital health implementation leader 4).*
- *“showing the benefits is really important and the value proposition. However, the sustainability of that in terms of the business model I think will be the biggest barrier, like who pays for it, because I mean if it can self-sustain itself, like sitting there without anyone working on it, that’s fine. But I don’t think [that’s the] plan with it. I think it’s around connecting – from what I can hear – connecting speech therapists, connecting clinicians. It’s almost like it’s got a 2-sided marketplace and I think it’s about following the train of I guess about the money and the purchasing power and why they’re doing it.” (Digital health implementation leader 3 with living experience of ABI).*

Sustainability was necessary to consider because;

- *“there are fixed costs, initially, and then ongoing costs in maintenance,” (Digital health implementation leader 1).*
- *“the insurance rates for holding information on your server, or any type of video or voice uploading, is extremely high. So, it’s something like [AUD]$2500 a month to have an insurance broker for holding that information.” (Digital health implementation leader 3 with living experience of ABI).*
- *“costing model is crucial, resource overhead to implement, human and cost resources to actually implement. What's it going to take? Do you have to buy a server? Do you have to have an IT team to manage it? You know, who is going to do all of that side of it? So what's the real cost of implementing this and what's the impost on the human and financial resources within the organization?” (Digital health implementation leader 4).*
- *“if you guys are going to keep developing this, you need some real income to do that and I think people generally would understand that in the world.” (Speech-language pathologist 3, Focus group 1)*
- *“As for who should pay for it, I think everything has a cost. That's just the way of the world. All the brilliant clinicians and researchers who put it together deserve some sort of remuneration for that, for their skills and their qualifications. But I guess it comes down to who pays for the physical, human, speech pathologists now. If you go to a speech pathologist, if you're seeing one in the public system, then the public system pays for the online Toolkit. If you're seeing someone through the private system, then you pay for that, whether it's through your own private health insurance or whatever it might be.” (Communication partner 4, Focus group 7).*

To achieve this, an expert in digital health implementation noted that;

*“things like other payers that are outside Medicare really have to be explored, or self-funded options. I don't think we can discontinue self-funded. I think clinicians hate the idea of self-funding programs, but there's lots of people that are happy to pay for a service if there's a real value in it. It might save them 1000 Ks in travel or whatever it may be to see that speechie [speech-language pathologist] each week, and they're really happy to contribute a couple hundred dollars each week to that program. I still think whether it's in this question or somewhere else, exploring privately funded models or a subscription service or something to this as a tool can have value.” (Digital health implementation leader 9).*

It was acknowledged;

*“it's a dirty phrase sometimes, but I think it's at least worth exploring, if people would self-fund. We know particularly in some of these areas that the financial distress is very high, and the roll-on effects of losing my job, like you said, even ending up homeless, is not going to contribute to people paying for these services or even having a laptop or a computer to access - or a tablet to access it anyway. But there's ways around that.” (Digital health implementation leader 9).*

It was also noted by carer that foreign nationals may need to access to self-funding if they were ineligible for domestic support;

*“I'm from [another country], [name of friend with ABI]’s from [another country] and we get no funding assistance at all. For anything. We don't qualify for NDIS [Australia’s National Disability Insurance Scheme]. We don't qualify for [State services] and [Australian Social Security] or any of those funds. So, everything with regard to [name of friend with ABI]’s recovery comes out of my pocket. And that's fine because we can afford to do it. However, I also accept that there's plenty of people that just simply can't, and so I can see both sides of it. I don't mind paying for the things that we use because that was something I accepted when we moved over here.” (Communication partner 3, Focus group 4).*

However, when self-funding was explored, people with ABI, their close others and clinicians responded negatively to the notion of people with ABI paying for the intervention, and rejected a self-funding model as inappropriate;

- *“I don't think anyone should have to do all this research for nothing. Someone should pay, but I don't think it should be the head-injured people that have to actually pay for it.” (Communication partner 11, Focus group 7).*
- *“I'm sorry, someone’s had a brain injury and we're going to punish them more? And say, ‘we can only help you by punishing you and making you pay’.” (Communication partner 1, Focus Group 2).*
- *“no, no, no, it should be free” (Communication partner 6, Focus group 6).*
- *“I want it available for everybody not [just] if they can afford it, but they're going to sell their arms and legs, like it should be everyone [can] get it” (Person with living experience of ABI 6, Focus Group 5 and 6).*
- *“with brain injury survivors there are a lot of extra costs involved et cetera and they’re on disability pensions and so if there was a cost involved, it wouldn't be able to be too great, I don't think. Just for personal users, they've already got their injury and yeah, they're out. I mean it's different for your hospitals, et cetera, but I'm with [Communication partner 6, Focus group 6] in that, it shouldn't cost anything or else there's some sort of reimbursement partial or full reimbursement, ’cause basically they're [not] doing it because [of] the choice they've made in life, unfortunately. Like, they need it.” (Communication partner 5, Focus group 6).*

Clinicians likewise highlighted socioeconomic considerations (Consideration 1M) noted in the ‘*First priority domain: Condition’* section as preventing some people from being able to self-fund, and for 1 clinician, the depth of feeling was emphasized in a follow-up email with suggestions of alternative funding models (Speech-language pathologist 2, Focus group 4).

Close others felt people with ABI were unlikely to be willing to self-fund an intervention;

- *“most people who have acquired brain injury are very careful with their expenditure, and I think that that would be inviting a waste of resource to try to have the brain-injured person pay for it, they should have to work another way around that problem.” (Communication partner 10, Focus group 7).*
- *“it would better if it was free. If it cost money, [Daughter with ABI] wouldn't do it, I don't think. She certainly would if it was free. I don't know whether that's a head injury thing either or it's just [Daughter with ABI]'s nature” (Communication partner 11, Focus group 7).*
- *“[Spouse with ABI] would be exactly the same. If we had to pay for it out of our pocket, he would not be interested. But if we could claim it as therapy through NDIS then it might lean him more towards doing it. (Communication partner 4, Focus group 7).*

One person with ABI expressed willingness to split the costs with insurance, citing an unwillingness and potential outdatedness for taxpayers to cover the cost, and “I think that if the injured person is paying for the treatment sessions, they are more likely to value the service.” (Person with living experience of ABI 4, Focus Group 5). However, this was a contested view, with a counterpoint that; “there is a risk of paying and not doing - think of all the gym memberships people pay for and not use!” (Speech-language pathologist 2, Focus group 4).

Another person with ABI noted that it should be services paying for and thus valuing the intervention;

*“I do totally agree that something paid for is valued more and so I would also assume that the services should pay for it first, but when [Person with living experience of ABI 4, Focus Group 5] said, “why not the person involved?” I thought yeah, maybe there should be provision for that, if you stumble across it on the Internet, you have provision to do that. But first and foremost, I would think that the service or you know, [Rehabilitation Facility name] or [Insurance] or something should pay.” (Person with living experience of ABI 1, Focus Group 2).*

###### Strategy 3.12 – Adopt a hierarchical pricing structure with least to no payment from people with ABI, and most from organizations, government, or insurance

Overall, all focus groups preferred;

*“if there was some sort of hierarchical aspect to the payment, [because] metaphorically and literally, the person who's already paying the most is the TBI sufferer, so if there was someone you’d wanna negate payments to, it’d be the TBI sufferers, and divvy up the costs. You could even provide it for, at least a reduced rate to the TBI sufferers and their families, and work upwards from there.” (Person with living experience of ABI 7, Focus group 4).*

Stakeholders and leaders in digital health implementation saw insurance schemes as the most appropriate funding source for the interventions;

- *“I was funded by [insurance] because I had [a motor vehicle accident].” (Person with living experience of ABI 3, Focus Group 2).*
- *“quite often people who are injured in accidents have brain injuries are receiving workers’ comp[ensation], Compensation of some kind and quite often the insurance will pay for the therapy.” (Person with living experience of ABI 4, Focus Group 5).*
- *“The model is that the government pays for everything. I would imagine that in Australia for the outpatient context, it would be the NDIS [National Disability Insurance Scheme] paying or the insurance company or whoever is funding the therapy primarily. I know there are private paying patients, but that's not a great proportion of people 'cause it's so expensive to have speech path[ology] input. But I wouldn't imagine it would be actually the families or the patient paying out of pocket themselves. It would be their representing funding body or the government service, or like a license.” (Speech-language pathologist 4, Focus group 1).*
- *“maybe there's opportunities with private health insurers, as well. There's 35 private health insurers that can provide funding in Australia. I think as soon as you go internationally, obviously, it becomes harder to navigate through that, but there are different insurers that work, say, in Asia, so life insurers, other insurers that work across - New Zealand, as an example, for an emerging area.” (Digital health implementation leader 9).*
- *“Once [Spouse with ABI’s] community therapy ended, all his other therapies after that we can claim as NDIS [National Disability Insurance Scheme], so it should be I think the Social Brain Toolkit should be a registered item on the NDIS, if it's proven to be successful, which it sounds like it would be.” (Communication partner 4, Focus group 7).*

However, many participants reported not qualifying for or being connected to the National Disability Insurance Scheme, and suggested Medicare as the preferred alternative;

- *“For me, for me I don't get NDIS at the moment, [therefore] Medicare [is] probably the most logical one. (Person with living experience of ABI 6, Focus Group 5 and 6).*
- *“[it should be covered by Medicare] Just like when people see [a] doctor. Let's say if the people resist it and if [the] government accept it then say, ‘look, just bill it with the time frame’, let's say this person spends 10 hours with the course, how much money [per hour], then work that out. And the person and the family of the injured people, they don't need to worry about the money at that time. [because] At that time, money is very important, so don’t do that, but do [it] in the back end with the government, Medicare.” (Communication partner 6, Focus group 6).*

A caveat to a Medicare-funded model was that,

*“a Medicare-funded model through things like chronic disease management plans - again, isn't really conducive to something that really - scalability. There's a lot of barriers with that.” (Digital health implementation leader 9).*

Sponsors or organizational endorsement and grants were also nominated, particularly for initial costs;

- *“If you want, do a free model and then do a premium paid model where [organizations] can put their logo in the corner or some rubbish like that, there would be people who would pay for that.” (Communication partner 2, Focus Group 3).*
- *“you need to find some sort of sponsorship. Yes, you can get sponsorship from [the rehabilitation facility], they've got the research department over there. […] for initial setup. […] some sort of sport club, because all the people with sport, they get the brain injury.” (Communication partner 6, Focus group 6).*
- *“there is some NDIS grants available. […] bits and pieces that you can cobble together that might allow you to put together a model of care within a certain payer - if we call it a payer. But it's not so much - there's a lag time and they're slow. These big companies are very slow, often, in terms of making things work.” (Digital health implementation leader 9).*

Since carers prefer to be referred by a trusted clinician (Strategy 3.3); “that means that they're [clinicians] probably going to be the ones that I think would be paying for it.” (Communication partner 3, Focus group 4). For clinicians were the payer;

*“You've got a couple of options. Your first option is to sell it to patients as a mobile app that they use to improve their training. If you do it as a mobile app, out of the app store, it can't be more than, let's say $3.99, a month or something. It's small but you get paid by thousands and thousands of people using it. So that can work. I would not take that route because you want it to be clinically validated and you want it to be a clinical tool and you want it to be used by all clinicians as part of their therapy. So, I would actually set it up as a clinician-only solution. So, the clinician has to sign up with you. I would set it up as a clinician solution and the clinician pays, let's say per customer, let's say $20 per month or something like that. The clinician maybe pays - I don't know - $100 a month and then 2 dollars per customer or something like that. You'll have to think of a clinician approach to that.” (Digital health implementation leader 7).*

However, there was a potentially unintended effect of penalizing continued use if users were charged over time or per use as in a subscription model;

*“I can't see a business model around software that doesn't involve a subscription that is sustainable. I mean the only other thing you could do is charge per consult or charge per use. That typically in healthcare will have the opposite effect. If you charge every time a person interacts with your program, then they'll stop using it because they pay for usage. That has the opposite effect of what you want to achieve.” (Digital health implementation leader 7).*

Some clinicians noted that a price inferred connotations of quality compared to free resources;

- *“there’s almost a sense sometimes that if something is free, it can't be that good or it can't all that great a quality or the evidence base can't be there, because why would it be free? And that having even just like a $20 price tag for the [App name] app you’re like, ‘that's more legit than the free one.’ Yeah, so like. I think it's...just a funny balance between like making it affordable like a token amount of money, that you, knowing that you're not actually really you're not, it’s not actually about getting profit, it's about making it seem more reputable or more legitimate because it has a price rather than being free.” (Speech-language pathologist 4, Focus group 1).*
- *“I totally agree with [Speech-language pathologist 4, Focus group 1] that it gives it that element of quality.” (Speech-language pathologist 3, Focus group 1).*

However, a clinician expressed desire for the Toolkit to be free;

*“I would love for there to be a way that some kind of grant is such that it looks after the ongoing costs that you talked about, but also means that services can use it, cause I think you might get quicker uptake, or maybe even at least for a trial phase where it's free just to make sure that people start using it and then they can advocate to their service,” (Speech-language pathologist 2, Focus group 4).*

A close other with experience managing a service provider within Australia’s National Disability Insurance Scheme suggested a way to confer the quality of a price point, while ensuring there was no prohibitive cost:

*“sell it as a paid service and discount it 100% for different groups -- that just happens to be everyone.” (Communication partner 2, Focus Group 3).*

Clinicians objected to a subscription model, citing recurring costs against sporadic use. They therefore preferred either the option to pause subscriptions or make a single upfront payment;

- *“the subscription thing, do [it] like a gym membership where you can place it on hold, so recurring monthly costs for the service that you can put on hold.” (Speech-language pathologist 1, Focus group 6).*
- *“I can't pay a subscription if I've got like 1 patient every so often that I do it with, I’m not paying £12 a month, […] It is a normal amount, but they add up, don't they? […] Don’t do a subscription!” (Speech-language pathologist 3, Focus group 1).*
- *“I agree, it’s way more likely you pay 1 lump sum at the beginning that you know what it is and it's not repeating, way more likely to invest in that. Yes, don't do [a subscription]! ‘Cause again, you might use it with 4 people in a month and then I have a dry spell for 6 months where it's not suitable for anyone and then suddenly, you know. So yeah, that 1 initial sum is really valuable.” (Speech-language pathologist 4, Focus group 1).*

There was also distinction between cost models for tools, with the clinician-led tool, convers-ABI-lity, more likely to be paid for than self-directed components;

- *“education stuff [interact-ABI-lity and social-ABI-lity] is free, but the package that I might use as a clinician [convers-ABI-lity, I could imagine buying that as a package. But, like $50, I'm being able to then use that with... because I'm buying your research, I'm buying your experience and I'm buying your way of doing things. That's a valid...that's a model we all use all the time, isn't it?” (Speech-language pathologist 3, Focus group 1).*
- *“Oh, I totally get that, you kind of just want to give it away in the sense that you know it’s going to help, this is the reason we do clinical work is to help the patient, so – and I think that’s okay as well, to let that shine through, saying we’ve spent a lot of time but this is really valuable to just have it out there and benefitting who it needs to. Then in return, you’ll take a cut from the clinician’s clinic appointment or something like that.” (Digital health implementation leader 3 with living experience of ABI).*

For example, clinicians thought it was reasonable to pay for training;

- *“I'm sure you're familiar with if you buy a test, for example, usually there comes a training [package] with it, and then you get certified for it and then then you can apply it.” (Speech-language pathologist 5, Focus group 1).*
- *“I'm thinking of other treatments that we have where you do have to pay to learn from a clinician’s point of view, you have to pay to learn the treatment, like LSVT [Lee Silverman Voice Treatment] or something. I guess if you know that the treatment is going to be done in the correct way, you can then get more treatment fidelity if you do have to be trained to do it, I guess anyone can just do this course however they want you don't. It's not guaranteed that it's going to be done the way that it's intended to be done.” (Speech-language pathologist 1, Focus group 6).*

This relates to a suggestion for clinicians to receive clinical training in the delivery of the program (Strategy 4.14).

It was also possible for organizations to be the payer;

- *“an organization could buy a license for the software and then it can be used on multiple devices. Certainly, that's often how things in government work. They pay for this license and use it on the computers. And that it would not be the patients paying for it. But again, I don't work in private, so that's an unfamiliar...” (Speech-language pathologist 4, Focus group 1).*
- *“I would hope that the hospital would pay, and I'm working in the public service anyway, so that's still part of the government.” (Person with clinical and living experience of ABI 8, Focus Group 3).*
- *“As the [NDIS] provider, you get funded per individual, and then for home care these staff work across multiple individuals and so you have to allocate that from like administrative funding rather than someone’s package or something like that, so I would not be averse to free. […] [in terms of] cost allocation for us with 250 staff. If I had to pay per staff member, or even if I had to pay as a group, maybe it only applies to half of the clients. I could allocate that. If you can do it for free, I would strongly encourage that -- we would pick it up.” (Communication partner 2, Focus Group 3).*

An expert in digital health implementation acknowledged the complexity of needing so many payment options;

*“as soon as you start going, we're going to fund this through the NDIS plus all these different insurers, plus potentially self-funding, it's a bit of a nightmare.” (Digital health implementation leader 9).*

##### Consideration 3H - Scalability

The self-directed and clinician-led tools in the Social Brain Toolkit had the option to be scaled up in different ways for different purposes. For example, although self-directed ‘Massive Open Online Courses’ could scale more readily at an international level, an expert in digital health implementation noted;

*“I think there's still scalability options with the third arm [convers-ABI-lity]. So, the telehealth component, you still need health professionals to deliver that, so 1 session a week for 10 weeks isn't really getting away from the problem of [scalability] - from a workforce perspective. If there's workforce issues, then that doesn't really assist with that unfortunately. But it does mean equality of access in some way, that why - because I live out in a remote area or I've got other disabilities that limit me accessing the clinic that could be 2 K[ilometer]s away or I have poorer supports, why don't I get access to these post-injury therapy services? So yeah, I think the premise of these interventions is really there.” (Digital health implementation leader 9).*

Likewise, the potential for the Toolkit to be culturally adapted and translated into other languages may not only increase international reach, but also had a value proposition for a multicultural society;

*“we in Australia we have how many people, how many different countries and nations living together? So, we need to emphasize that one for the government.” (Communication partner 6, Focus group 6).*

###### Strategy 3.13 – Adopt a strategic, stepwise approach to scaleup

Although web-based delivery offers the possibility of scaling up a digital health intervention, leaders in digital health implementation cautioned against immediate global release. An expert in digital health implementation provided insight into the risks associated with scaling too quickly;

*“[The consequence and worst case scenario of doing everything at once include the risk that] the team would break down. I think there'd be multiple mistakes, errors. You'll lose your reputation very quickly. So, risk to reputation, risk to business, risk to funding, burnout of your internal team, burnout of consumers. If you get something - it's a complex intervention you're trying to implement, and you get it wrong, and badly wrong, it'll be quite catastrophic. Everyone will tell you that you need to scale up and go hard immediately, but most successful companies have taken a very long time to get up and running. Microsoft, Apple, they were operating for 10, 20 years before they scaled. Most people don’t know that. Success often doesn’t happen overnight. Most success actually takes a long time.” (Digital health implementation leader 1).*

Instead, their expert recommendation was a strategic, planned approach to scaling;

- *“No [don’t make it freely available online for everyone immediately]. Be strategic. I think you need to develop or clarify your strategic objectives and the timeline. That is, once you meet certain milestones in terms of number of users, and satisfaction rates and whatever, then you can move to the next point in the strategic plan, but don’t try and do everything at once.” (Digital health implementation leader 1).*
- *“I would be inclined, in terms of the rollout and testing, just small, careful, develop over time, as opposed to try to roll out too quickly.” (Digital health implementation leader 1).*

The stepwise approach should start with the tool most likely to succeed at scale;

- *“just be cautious about it. I would, rather than necessarily roll out 3 whole new paradigms, I'll begin with 1, the simplest, the most engaging, because as you roll the 1 out, your - the message is much simpler for people to understand. If you can't roll 1 out, that is, uptake isn't acceptable, or people don’t want to use it, then [it is unlikely] you could roll out [3].” (Digital health implementation leader 1).*
- *“We invariably find so much complexity and so many potential points of failure, that I'd certainly be encouraging other groups, if they're rolling out something quite complex for the first time, to try to simplify it. Go for the minimum viable products. Go for something that can succeed, relatively easily.” (Digital health implementation leader 1).*
- *“You don’t have to scale straight away. Get the product out there. Enhance it, improve it, make it the world's best, then scale. Scaling's not the same as implementation. […] It requires different skill sets, different strategic imperatives and different funding streams, and so on. People get the two confused. So, start off with a product that you know several thousand people will use, or whatever it might be, and that you like, and build off that success.” (Digital health implementation leader 1).*

Another expert in digital health implementation had the same view;

*“not [released] to the masses, no. But [there are lovely things] from clinical trials to bring out, like as in you can release it and you’re releasing it in alpha. You’ve got your alpha product out there and then you might make that a locked website that’s available with a [passcode], so it’s [not as a] paid product, but it’s called an alpha test. Then you send it out and you get people and feedback and do an interview like this afterwards, get to talk to them and then you’ll make changes and release a beta. Your beta group might include your alpha group as well. Sometimes it’s a total new group. You might be got the wrong market fit, so you kind of like – sometimes you might even run something like 5 alpha groups. So, you might run it with one population and then another and you see who it’s hitting and so, yeah, to release you don’t have to release- you can release in soft form. […] Then, when you’re ready, then you’re unlocking the website.” (Digital health implementation leader 3 with living experience of ABI).*

Multiple participants suggested demonstrating initial value through free versions before pursuing commercialization;

- *“Just in terms of strategy, what you may want to do is initially trial a free version, and test it, a model where existing therapists, speech-language therapists, could use it for free, and easily. Essentially, if everyone finds it helpful and useful, then you add value to the intervention over time, and the experience. Then you might actually add a commercial arm to it, as well, because once it's accepted as safe and effective, then it's much easier to become commercial. Whereas if you try to become commercial too soon, without having demonstrated or tested the value proposition, the appetite, without having essentially improved, and just rated the program, you'll be behind the 8-ball, and having to retrofit, which is always very challenging.” (Digital health implementation leader 1).*
- *“I love the idea that you’re going to offer modules for free, because building up traction and testing the products [with the barriers] to see if they’re actually doing what they intend to do, reaching the audience, that kind of stuff is incredibly valuable to start with. [Otherwise] you wouldn’t have enough data to tell an insurance company it’s valuable to pay for, or Medicare, if you weren’t feeling like people were coming to the site to use it.” (Digital health implementation leader 3 with living experience of ABI).*
- *“as a promotion maybe at the beginning because it's something you then they can be either free or cheap at the beginning and then the more you also get feedback you the more you will develop the app and everything so it will get better. It will get a better quality and then maybe you can later on consider having it with the price because you have to do some.... because I'm thinking of promotional at the moment and if you if you have to make it appealing to the buyers so that they can give it a try. If it was something well known. And with the price everyone is like oh it must be good quality, yes? but it has time to get there. Um, so at the beginning as a start it has to come with some free features, at least for the first tries, and then later you can try it. Or, as I said, I don't really like it, but I think it's a lot of out there. Maybe because it's working, like some features can be free, some features can be extra valuable things. You know. I hate when apps do that like, “To reach this you have to go to Premium” I hate that, but it's a lot out there. So now I'm thinking probably it's working, that's why it's out there because some people maybe need just free materials, just the basics.” (Speech-language pathologist 5, Focus group 1).*

While progressing through each stage of development, an expert in digital health implementation noted the value of a website to communicate with individuals interested in the development of the Toolkit;

*“you should have a landing page about – so you might need 2 pages. So, you want a page that says this is me, this is us, this is all the people involved, so if people do want to go and check back on what they’ve done or before they say yes, they want to check you out. You need to have a presence. You need to be legitimate and you have we’re in alpha trials and you can have something similar, like you might have some sort of graphic about how many people you’ve had in your alpha trial and then……when you go to – your alpha trial is finished, you might have a sign saying coming soon and that’s a sign that you’re usually in a beta trial. I think you think about it as a clinical trial, a release and document all the data around that, but you have 2 separate pages. So, this doesn’t mean 2 different domains, it just means your web page has front, like open access, so anyone can see it. Then you have locked pages that require passwords. You can put anything on those. You might even have multiple versions of your Toolkit that […] somebody gets Version A and someone gets Version B and you’re running and testing these.” (Digital health implementation leader 3 with living experience of ABI).*

A close other noted from the communication partner perspective that international translations could be forecast as part of the longer-term potential of the Toolkit, but would only occur after initial success with English versions:

*“we live in Australia and you need to think a little bit ahead, with multiculture, let's say you, in the future we've got forecasting of 4-5 different languages […] we need to put together the picture to the government, say "these are the way we are going to do in the future, this [is] only first step […] that [is] the future. You need to when you get success. We need to get up to that. [It’s] a bit too early now.” (Communication partner 6, Focus group 6).*

### Fourth priority domain: Adopters

*All users*

##### Consideration 4A – User experience

Leaders in digital implementation identified the need to ensure “the user experience is smooth, and they don’t have a block, in terms of the software platform.” (Digital health implementation leader 1). Such consideration needed to include all user perspectives;

*“always have all the participants in mind. There's your clinician that needs an oversight over what the patients are doing that comes through a clinician dashboard; the patient who needs to see that they're making progress, that they're having fun and the third is obviously you trying to make a business out of it. You have to really consider all the all the people, all the personas involved in running this.” (Digital health implementation leader 7).*

For example, from the clinician perspective:

- *“with any technology, if you're having to spend 20 minutes setting it up and dealing with glitches and things, it's much less likely to be used by the clinician.” (Speech-language pathologist 1, Focus group 6).*
- *“Or clinicians getting a moment to do it, and then it doesn't work, all the other pressures bang up against it and they need to keep moving and it will drop out a bit or it will be difficult to implement,” (Speech-language pathologist 2, Focus group 4).*
- *“it certainly will impact on some people. I think if something isn’t simple, easy, works the first time, then it gets put in the too-hard basket.” (Digital health implementation leader 8).*

###### Strategy 4.1 – Conduct user or persona testing

User experience could be pre-emptively tested through user personas and the paradigm of user journeys, including the different considerations for people with ABI (Considerations 1A-1N in the *‘Priority domain 1: Condition’* section) and their close others (Considerations 4D-4H);

*“how many different consumer journeys have you mapped? When we're thinking about a service pathway, we'll map initially, 7 of 8 key personas, which we recognize from our user base, but then we'll model an additional 18 to 20, up to about 70 or so different directions and profiles. […] We have people specifically testing the different functionality between our software components, and pressure testing at the technology level, but they're based on user cases.” (Digital health implementation leader 1).*

This could be followed up with posthoc user observation;

*“you might need to just see some people using it with these conditions to understand why for instance, is this person ceasing the training? You might know that they've ceased, you know it at this point in the process from the software, [but] you might not necessarily know why. It could be just a really basic thing like the screen reader doesn't work on a certain page and they get stuck.” (Digital health implementation leader 5).*

In parallel, developers needed to be

*“thinking about the journey for the clinician, which occurs in parallel to the journey for the consumer, and even something as simple as having to have another screen or browser open on the computer can be quite challenging. Even this business of trying to actually share information or collect information, when they use a different electronic medical record software application [can be] challenging.” (Digital health implementation leader 1).*

This includes clinicians’ need to provide technological support to people with ABI and their close others (Consideration 4K).

*People with ABI*

Consideration 4B - Access to a viable setup

It was noted that not all people with ABI or their close others may have a viable setup for videocalls or self-directed work. For example;

*“because I'm not really well set up at home with a webcam and everything, I find it a bit difficult [to participate in video calls]” (Person with living experience of ABI 5, Focus Group 3).*

In the ‘*Second priority domain: Technology’* section, stakeholders discussed the challenges of setting up for video calls in terms of the key features of device stability (Consideration 2E), camera (Consideration 2B) and screen size (Consideration 2A), as well as the need for a person to help a person with ABI set up hardware (Strategy 2.5). Access to a viable setup may also depend on sociocultural factors, such as a person’s socioeconomic situation (Consideration 1M).

###### Strategy 4.2 – Provide a setup checklist

A potential way to minimize the complexity of setting up the required technology for an intervention could be to provide a checklist itemizing requirements in advance;

*“For me personally, I think that's massive [to have an upfront checklist of requirements] because we, doing this on my phone, which we had the chances of [knowing prior], then we wouldn't be doing it on the phone, and the list of things you may need, like [a] mouse, like for me, that's awesome, a big checklist.” (Person with living experience of ABI 6, Focus Group 5 and 6).*

##### Consideration 4C – Expectation to return and reorient to an intervention

Given all 3 tools in the Social Brain Toolkit require users to complete self-directed modules and self-reported measures, explicit consideration is needed of the assumption that people with ABI and their close others need to engage regularly with an intervention and reorient themselves to the task after each break.

###### Strategy 4.3 - Streamline the number of steps

An expert in digital health implementation explained that the burden or demand upon users could be reduced by streamlining the number of steps required;

*“[Every] time we ask someone to check an email or to log back in, requires new learning. I guess the theme I'm really talking about is trying to reduce burden, reduce demand.” (Digital health implementation leader 1).*

In addition to reducing steps, some could be transferred to the clinician;

*“every additional step required, even if it's pressing a button, it's associated with reduced adherence and engagement. What you may choose to do is flip the paradigm somewhat and actually have the therapist record some of the interviews [rather than expecting dyads to upload recordings]. So, you're embedding a key piece of monitoring, with a click, within context of a regular, scheduled appointment.” (Digital health implementation leader 1).*

This may also assist with challenges in concentration (Consideration 1B) and memory (Consideration 1D).

###### Strategy 4.4 – Recommend and provide reminders to complete the intervention at a regularly scheduled time

People with living experience of ABI agreed on the benefit of a regularly scheduled time to complete the intervention or attend appointments, particularly for avoiding scheduling conflicts with other commitments or requests;

- *“Could I suggest before you start actually having a set time, whether it be every second day or every third day or once a week or something like that, that you say I'm setting aside this time each week so you actually have it in your head that you're doing it at a certain time, and then everything else has to stop. […] that would be the only way I'd be able to do it.” (Person with living experience of ABI 1, Focus Group 2).*
- *“being part of routine if I know that I'm doing that at this time, I'm more likely [to do it] because I’ve got a whole bunch of things in my life, but if it's part of my routine I'm prepared, I know that I'm going to do that. I would put it in one day in the morning and make it part of a routine that I did maybe once a week, and that is what I think would be helpful for me. But I know everyone is different.” (Person with living experience of ABI 2, Focus Group 3).*
- *“once I’m in the process and the routine, I’m going to be able to do it much more straightforward than if I was just trying to figure out a way to fit this into my week’s work and other things I’m doing through the course of the week. It would be much easier.” (Person with living experience of ABI 9, Focus Group 5).*
- *“having a set routine, like everyone suggested, is very helpful.” (Communication partner 7, Focus Group 2).*
- *“I find that I'm driven by expectations, so if I know that I'm expected to turn up at a certain time every week, I can schedule that into my electronic diary and it happens! But if it’s all over the place, say I’m doing this 2-week conference at 12 o’clock on a Friday and then the next one’s going to be at 7 o’clock on a Tuesday night, it’s all over the place and I find that the routine, if you can get into a routine, then every day you wake up and you say well at 12 o’clock I’m going to be doing this teleconference, every Tuesday, so that’s just an issue that I would appreciate.” (Person with living experience of ABI 4, Focus Group 5).*
- *“Yep, it's Tuesday middle of the day -- I'm out, I can't come to your party. I can't go and pick you up from school. I can't do this 'cause I've got my Tuesday morning regular time slot – bang, done.” (Person with living experience of ABI 9, Focus Group 5).*
- *“I need a lot of structure, just on a personal level, to be well […] a timetable kind of approach would - it sounds kind of old-school, but that would be a good way for me to do it” (Person with living experience of ABI 10, Focus Group 3).*
- *“Me personally, like if you're due to have it like 1 o'clock every Monday or Friday, that would make me put it in my calendar. Because I live by my calendar [laughs].” (Person with living experience of ABI 6, Focus Group 5 and 6).*

Ideally, this routine could be supported with electronic reminders that reflect individual preferences:

- *“and then to be able to start like we said before being able to put reminders in place that matches the timetable -- that would be great. […] text [messages] would be good, but I guess that's probably pretty expensive as well. But failing that by email, I guess. Then I guess you just have to make sure you got your notifications on so you see it come up.” (Person with living experience of ABI 10, Focus Group 3).*
- *“I don't know what form reminders would take but if you could set those, what's the word, not tailored, but maybe if you could basically set your own parameters for when you get reminded based on how you intend to attack it, I think that would really help as well.” (Person with living experience of ABI 10, Focus Group 3)*

In terms of frequency of reminders,

*“weekly'd be spot on. Anything longer than that, then it wouldn't be worth it ’cause […] in 2 weeks going back […] catch up would be a nightmare. I just wouldn't go near it. If I went 2 weeks and missed it; I'd get... I'd be overwhelmed trying to catch up. Yep, I'd chuck it in the bin and give up.” (Person with living experience of ABI 6, Focus Group 5 and 6).*

###### Strategy 4.5 – Provide positive feedback of progress and achievement

Clinicians, close others, people with experience implementing digital health and a person with ABI felt positive reinforcement, including gamification, could support engagement with an intervention;

- *“We're really just supporting people to succeed on their journey; every time they read something, gosh, a flag goes up, or a thumbs up.” (Digital health implementation leader 1).*
- *“something that's very repetitive, that's gamified, that's fun. I think you really need to focus on making these people enjoy their interaction with technology”. (Digital health implementation leader 7).*
- *“hopefully some degree of fun, rehab’s torture enough!” (Speech-language pathologist 2, Focus group 4).*
- *“it reframes the way you look at something, it can be kind of difficult and whatever, but if there's a stimulating component to it, people will come back to it.” (Person with living experience of ABI 7, Focus group 4).*
- *“[With a web-based service delivery model], you could structure it differently. I think you could say there is something new for you to do every Monday, or something like that…and it’s not a commitment, but something that you – if you gamified it, like you’ll get another credit or certificate, or something that might appeal to the audience or the person, that means that they feel like they want to do it again.” (Digital health implementation leader 3 with living experience of ABI).*
- *“I really liked the “make it like a game” idea. I don't know how you could make it like a game, I'm not a gamer, but I do know that [Spouse with ABI] spends a large amount of time playing games on the iPad and they're all the kind of games that have a reward attached to them.” (Communication partner 4, Focus group 7).*
- *“He would respond really well to that [a percentage progress bar] […] That's his personality type; he's a competitive, results-driven person. For someone like him, if there is those “you're 60 per cent of the way through”, and “you're running on 90 per cent accuracy” or whatever it might be, he would really respond positively to that. I'm sure there's a lot of other people that are similar.” (Communication partner 4, Focus group 7).*

As a counterpart to avoiding a judgmental tone, this strategy may also assist support self-esteem (Consideration 1F).

However, some people with ABI described being sufficiently internally motivated as to not require external rewards:

- *“Me, personally, I couldn't care less about [a certificate]. But me improving myself, if I get enough benefit to myself, then that's my trophy.” (Person with living experience of ABI 6, Focus Group 5 and 6).*
- *“from my point of view, the goal that you’ve achieved doesn't require a certificate or something, it's the goal done, complete, next.” (**Person with living experience of ABI 9, Focus Group 5, agreed by Person with living experience of ABI 4, Focus Group 5).*

It was also noted that overly stimulating game design, or gamification itself, may not be necessary, and that any design should be respectful toward adult learners;

- *“For some, the colors and music and whatnot might be a bit overwhelming.” (Communication partner 5, Focus group 6).*
- *“it doesn’t have be profoundly fun and razzle dazzle and full of rewards but something that's got some sort of interface that is both really simple and easy to enjoy.” (Person with living experience of ABI 7, Focus group 4).*
- *“It's going to vary [whether people would like a game design]. […] as we all know, a great number of these people with acquired brain injury have very sound opinions, they're capable of deep thought but they just have trouble - they can't get it out quickly and you can't rush them, but I think it's one of the most important things is they should be all treated* like that *with respect to that. I think that they will become aware of their own success anyway as they come on.” (Communication partner 10, Focus group 7).*

The intrinsic value of the intervention to communication (Consideration 3A) was discussed in the ‘*Third priority domain: Value Proposition’* section.

Therefore, positive reinforcement may take the form of communicating tangible progress towards personal goals;

- *“you've got to demonstrate value to maintain people's engagement with these types of tools.” (Digital health implementation leader 4).*
- *from my perspective everything I do is about [name of friend with ABI]'s welfare, about his recovery, and about him having his needs met. And it's just about getting people to buy into it, from that point of view of, if you are up to speed with this, then this is how it's going to benefit you to help the person that you're helping. I think just a reward system of, why am I doing this? what's the point of this? is imperative to it, otherwise. people will start with a hiss and roar and then there’ll be a significant drop off if people don't have a clear understanding about ‘what's the point?’.” (Communication partner 3, Focus group 4).*
- *“You see yourself being able to talk better, to interact better. You can see it in everyday life, that your interactions are improving then it’s an effective program. You keep doing it.” (Digital health implementation leader 7).*
- *“part of adult learning and part of changing behaviors is actually what’s important to the person. […] If it’s not important to them or if they’re not ready to do it or if they’re not confident in doing it, they’re very unlikely to have success. So, if the first module, if they don’t feel that they’re ready to do it or that it’s not important to them, they’re very unlikely to succeed with it, whereas they could look at the modules and say actually module 3, I really want to do that, I’m ready to do it, it’s important to me, then they need to have the option of doing that.” (Digital health implementation leader 8).*
- *“Yes, yes, it is, it's a big help. It's a big help... to get us through.” (Communication partner 6, Focus group 6).*
- *“if you highlight, “hey, if you do use this the potential benefit could be this, this, this and this” and that might motivate people more because they might be thinking, “Oh well, what could I get out from this? What would be the point?” (Person with living experience of ABI 2, Focus Group 3).*
- *Once we get started on the Social [Brain] Toolkit like the benefits that we’ll gain are just so much, that will keep us going, in itself.” (Person with living experience of ABI 5, Focus Group 3) and “I also like the idea of seeing the benefits of it up front in terms of what the outcome will be for us. I really like that idea as well.” (Person with living experience of ABI 10, Focus Group 3) and “I think we should be hearing from people who have benefited from the program; like people themselves. I reckon that would help us get online and do it a lot better.” (Person with living experience of ABI 5, Focus Group 3).*
- *“But I think that when things are rewarding and you can see that it has benefit then I think it's easier, and I suppose with some processes it can be very slow. Yeah, you think this is really worthwhile and so then you’re motivated to do it and so then you do keep appointments, you honor them.” (Communication partner 1, Focus Group 2).*
- *“seeing the benefit would actually be more motivating than anything else.” (Person with living experience of ABI 1, Focus Group 2).*
- *“If you can have mini goals along the way, because quite often if you're doing a therapy course like you've got in mind for us, you sort of have an end goal, but it seems so far away and little steps that you take each time you do a therapy course seems pretty unremarkable, but if you have set yourself mini goals each week or each month and then ticked it off when you achieve those goals, I think that shows, like a road map that to your final destination. You know, rather than just get everyone to do 6 courses without any sort of measure, along the way. in my view, [it’s] preferable to have each little course that you do, an end goal like an interim goal.” (Person with living experience of ABI 4, Focus Group 5).*
- *“As time progresses your goals change – in-depth, if you will. So, you should always update your goals on a regular basis. You achieve this, what's the next step? Achieve this, next step. There’s always going to be progress. Even if you haven't got injuries like we do. It's still learning, everyone’s still improving, so everyone has to have goals.” (Person with living experience of ABI 3, Focus Group 2).*
- *“achievable goals, too. Because then you feel better after about a little bit. There was a quote in my ABI group, because when you first hit hospitalization, getting better just looks like a mountain, but if you just do one little step, just one little thing, a little bit regularly... So achievable goals: just start small and then you won’t die.” (Person with living experience of ABI 2, Focus Group 3).*

###### Strategy 4.6 – Provide upfront communication of the required time commitment

All stakeholder types described the benefits of communicating the expected duration and frequency of engagement with the tools;

- *“it's being as transparent as possible, and shows you how long each section of the course will take at a normal pace, cause I could represent the country in laziness and that would tell me pretty quickly whether I was prepared to engage with it properly or not rather than start, do a half-arsed job and never finish it and probably never go back to it, or just be a bad experience all of my own doing, so if I knew upfront what sort of a commitment and how I could manage that with whatever schedule I had at that moment that would be a big benefit to me.” (Person with living experience of ABI 10, Focus Group 3).*
- *“if you could at the beginning just sort of say […] we suggest the program is completed in this amount of time, so just setting up that expectation up from the beginning.” (Speech-language pathologist 1, Focus group 6).*
- *“Yeah, ’cause I think if it is a bit unclear how long it takes then that's when you might get people who dabble in it every now and again over many weeks and that's where issues with forgetting and having to go back could come in.” (Speech-language pathologist 1, Focus group 6).*
- *“perhaps..[you] could give a recommended outline, like you do it, like an hour every week you'd be completed by this date.” (Person with living experience of ABI 6, Focus Group 5 and 6).*

However, it was noted that;

*“it needs to be strongly emphasized that it might be a suggested pace and not, 'okay, this will take 10 hours this month’; it should be a suggested time frame so that they don't feel bad if they are falling behind. So once again, you might get to the stage where they might just find it too hard” (Communication partner 5, Focus group 6).*

This may relate to the concepts of avoiding conveying judgment and penalty (Strategy 1.11).

An expert in digital health implementation reported that;

*“we know the research around interventions that are 8 to 16 weeks are typically well received, 8 to 12 even better. The engagement with that - if the concerns are, okay, how many hours or how much time is this going to take me person week is typically the question. If that is less than an hour - this is just anecdotally, but from our experience, an hour or so per week, then that's okay, great. I can make it work.” (Digital health implementation leader 9).*

###### Strategy 4.7 – Provide printable reference materials

Although digital health interventions are primarily delivered through devices, communication partners noted the benefit of being able to print hard copies of reference materials;

- *“But even with [Person with living experience of ABI 6, Focus Group 5 and 6], he's high-functioning, I think it would even still help him because of his short-term memory loss to have some reference material beside the computer as well as the back and forth, and just to have something to look at to reference.” (Communication partner 5, Focus group 6).*
- *“having the paper copy of it is just critical also.” (Communication partner 10, Focus group 7).*

This thus supports memory impairments (Consideration 1D).

###### Strategy 4.8 – Facilitate accountability to a clinician

Accountability to a clinician was observed to be beneficial to the completion of self-directed intervention tasks;

- *“From our experience, the other big thing that helped: [Person with living experience of ABI 2, Focus Group 3] got a lot of homework when she did speech [therapy] and that would always be done […] At least by the night before. Yeah, because she didn't want to go to her speech therapist not having done the homework.” (Communication partner 2, Focus Group 3).*
- *“[the] effect of having him [the clinician] in that third cohort was crucial to […] engagement with the program. So, I think that you know, it's that kind of evidence that gives me the indicator that for people to sustain engagement, again, it has to be part of that therapeutic build. You know, it has to be seen to be important by both sides. [It] Has to be used regularly. Otherwise, it will just sit there.” (Digital health implementation leader 4).*
- *“even though the virtual one can help, if we have personal support by the actual person with you, the human touch is always good. […] I would love to have, every now and then, actual contact with a human rather than just machine itself. I know it is hard.” (Communication partner 7, Focus Group 2).*

It was noted that accountability to a clinician could be effective due to the informal social contract of honoring agreements;

- *“usually we schedule the next time we’re going to get together, and we’re very loyal, we keep to the time.” (Communication partner 1, Focus Group 2).*
- *“it's the capacity for you to do what you said you’re going to do, and you've given someone your word that you're going to be there and through it, do the processes running through the course and do whatever you’ve got to do to run through the course then I think it's up to you to fit yourself around that requirement, as it were.” (Person with living experience of ABI 9, Focus Group 5).*

However, these agreements could also be formalized;

*“we have a coaching agreement that we ask each side to - the actual clinician and the coachee to sign, to say this is the expectations for you for doing this program. If you don't agree with these expectations, then it's the wrong program for you.” […] we're soft in how we talk about that, but we try and have clear standards. Essentially, not a contract, but I'm signing up to this, I want to take it seriously. I think it's going to have benefit to me, so therefore I'm going to engage with it. I understand it's not going to be a perfect trajectory, but the other person's also putting their time and effort, and I have to respect that. So, the speech pathologist on the other side doesn't have 1000 hours of their time. They're dedicating this to you in a caring way.” (Digital health implementation leader 9).*

It was suggested that video calls could facilitate this clinician contact while overcoming difficulties accessing face-to-face services; “[Face-to-face contact] would be nice, yes, but unfortunately some of us aren't that [geographically] close by anymore.” (Person with living experience of ABI 3, Focus Group 2). Therefore, it was noted that clinician contact through video calls would be “excellent” (Communication partner 7, Focus Group 2).

###### Strategy 4.9 – Facilitate accountability to peers or mentors with ABI

In 1 focus group, people with ABI suggested the benefit of peer support;

- *“working in groups works for me. Like if [Person with living experience of ABI 9, Focus Group 5] and I were working as a team, right, and we were meeting at say 12 o'clock every Tuesday and we had a certain deliverable that we had to meet, then he could motivate me to get my bit done and I can motivate him. Working as a team is often a good motivator for me.” […] “at the end of the completion of the course we'd all go out and have a big cup of coffee together or something like that.” (Person with living experience of ABI 4, Focus Group 5).*
- *“Maybe some contact with other members of the team might help. To say, you should be doing X and Y. Have you finished X and Y? If you’ve finished X and Y, you could help me do Z, for example, or something like that.” (Person with living experience of ABI 9, Focus Group 5).*

Similarly, in another focus group, a communication partner suggested;

*“I'm wondering if it would be useful when someone had gone through the course and had already graduated, to become a partner with someone who you are introducing it to? Given that people had the skill, or they got on [with each other], because I think, then you've got someone being mentored by a graduate.” (Communication partner 1, Focus Group 2).*

#### Close others

##### Consideration 4D – Performance anxiety

A communication partner observed that some family members felt anxious about the appropriateness of their communication with the person with ABI, which may hinder their engagement with communication partner training;

*“I feel like other people, extended people around [Spouse with ABI] perhaps also feel like they're going to do it wrong or they're going to be judged. […] That that they're not doing it right. They're not doing it right so I'm just not going to do it.” (Communication partner 4, Focus group 7).*

This was addressed by Strategy 3.5 to ensure empowering communication with close others.

##### Consideration 4E – Familiarity with tool

Communication partners who wanted to support the person with ABI to complete self-directed work described familiarizing themselves with an intervention in advance;

- *“even doing the course that the person with the brain injury will be doing is really useful: it’s like you're studying something ahead of them. So, from the get-go you know how it works, you avoid those frustrations, and you make using it the first time quicker and easier, which is a huge thing because if you're sitting there trying to figure it out then it's a much longer process for them, more fatigue, etc.” (Communication partner 2, Focus Group 3).*
- *“Whenever you do any of these things it just pays to learn everything you possibly can about it. That's what will help those people to get the very most out of those courses, or for anything that they try to do.” (Communication partner 4, Focus group 7).*

This pathway through the intervention may be investigated through user or persona testing (Strategy 4.1) as above.

##### Consideration 4F – Expectation to provide social support

Communication partners recalled from their experience that; “family support is more than 50% of the help of the recovery.” (Communication partner 7, Focus Group 2). Support included contributing toward communication goals;

*“You had different goals. You would tell us what your goals were because they were not things that I would ever have picked up, because when you want someone to communicate with you, you accept any form of communication. But for [Person with living experience of ABI 1, Focus Group 2], she was looking to always better her communication and it was useful for us to know what the goals were. So, I think that could be helpful as well because it was helpful to us.” (Communication partner 1, Focus Group 2).*

This consideration thus relates to empowering close others (Consideration 3B) in the *‘Third priority domain: Value proposition’* section and the benefit of seeing tangible progress (Strategy 4.5).

It also included practical support and encouragement to complete the intervention;

- *“I'll just go back to what [Communication partner 4, Focus group 7] said. I think it's absolutely important that there's got to be a person to back them up, even to prompt them on, to help them, to encourage them.” (Communication partner 10, Focus group 7).*
- *“If there was a person there that could try to make at least some of their time to help the brain-injured person, I think it would be huge, make a huge difference. And tell them that they've done a good job when they've done a good job and kept their appointments and done all that, I think. It's pretty hard for them otherwise because they would just forget or remember then forget, or some little thing comes up that they can't do so they miss the whole thing. There wouldn't be anybody I think that has a close association with a brain-injured person that wouldn't go through all that.” (Communication partner 10, Focus group 7).*
- *“I think sharing with it them is okay, well now, ‘remember we've got to have our computer session at such and such a time, what did we do last week, and oh yeah, that was pretty interesting, wasn't it, or last time, and what are we going to do this time’, and any problems they had encountered. Then you can help them through that.” (Communication partner 10, Focus group 7).*

Whether this expectation is met depends on the availability and nature of social support (Consideration 1K).

##### Consideration 4G – Expectation to provide technological support

It was highlighted by communication partners, clinicians, and leaders in digital health implementation that close others often need to support a person with ABI to use technology;

- *“the thing for me with the Social Brain Toolkit is that someone who couldn't work the technology on their own, there has to be someone else.” (Communication partner 4, Focus group 7).*
- *“we usually have a family member setting up everything for them, pressing the buttons, connecting with me because I send them a request.” (Speech-language pathologist 5, Focus group 1).*
- *“in terms of the technology, I've mentioned already just the general challenges around that population and obviously potentially requiring carer support to navigate it.” (Digital health implementation leader 5).*
- *“I have seen how old people are supported by perhaps not children but grandchildren. It's interesting to see how they come together in a network of help. That would also be - yeah, or friends. I think it's interesting how people support brain injured and could that play a role if they're very much into the digital solutions.” (Digital health implementation leader 2).*

A person with ABI could experience difficulty if this technological support was not available from close others;

- *“he's not opposed to a telehealth call, but he couldn't start one himself.” (Communication partner 4, Focus group 7).*
- *“I have one client - he can do everything. He can push buttons, go between the tabs and everything, but once. For example, our connection was lost and the, the carer wasn't there, so we couldn’t reconnect again.” (Speech-language pathologist 5, Focus group 1).*

The capacity to meet this expectation depends on the availability and nature of social support (Consideration 1K) and their digital health literacy (Consideration 2L).

##### Consideration 4H – Expectation to participate in interventions

It is notable that close others may need to participate directly in communication partner training programs as a communication partner;

*“If so, what I would do is say to the communication partner, ‘I need you to come in, like, for this to be beneficial you need to come in at least once a week and we’ll, all 3 of us will sit down and do it. So, I'd be present for that and then I would hopefully be able to use it, the package whereby the rest of the module they can kind of do by themselves at home and it, and so that they do all the learning at home. And then they come with their person with a brain injury and kind of implement strategies or do the videoing,” (Speech-language pathologist 4, Focus group 1).*

Some communication partners might have both the motivation and capacity to participate:

*“It was really me who was giving him the therapy and so that's how I've looked at all the therapies for [Spouse with ABI]. I would go to as many of his sessions when he was an inpatient as possible, so that I could learn what we need to do when we're at home, and that continues now.” (Communication partner 4, Focus group 7).*

Others may be willing to participate but struggling under caregiver burden;

*“people [who] support people with the [brain] injury. It's a very, very hard time for them. I've been through that, I know it. Yeah, don't, don't push. It's good to remind, it's good to remind. Yeah. But don't push.” (Communication partner 6, Focus group 6).*

Other close others may have neither the willingness no capacity;

*“The [adult] children are not really interested in helping their parents, because I usually see elderly clients, they have their own life. They just put them there in front of the camera and then they leave and sometimes partners are involved or sometimes as I said, they are not really involved or they don't really understand what's with the technology.” (Speech-language pathologist 5, Focus group 1).*

Whether this expectation is met depends on the availability and nature of social support (Consideration 1K).

###### Strategy 4.10 – Facilitate buddy or peer support for close others

A potential way to support close others may be to connect them to peers;

*“I don't have any brain injury, but doing anything by myself is hard, even though I know the benefit of that. […] so, to me, if there is an online video call, doing it with a virtual group, I think it's more likely to happen than doing it by yourself.” (Communication partner 7, Focus Group 2).*

This may include connecting one pair or dyad with another;

*“That's a great idea. I think that’s excellent. [to be paired with a buddy or two who are completing the treatment at the same time]” (Person with living experience of ABI 1, Focus Group 2).*

###### Strategy 4.11 – Provide reminders for close others

To support close others,

*“reminders are good. I already use - we already have a family calendar, so for [Spouse with ABI] I already put his things in that he's got to do that will pop up. I have a calendar for him, I have a calendar for us, I have a calendar for the kids, I have all of this stuff.” (Communication partner 4, Focus group 7).*

This strategy may also support Strategy 1.7 to reduce memory requirements for people with ABI in the *‘First priority domain: Condition’* section.

#### Clinicians

##### Consideration 4I – Responsibility to support and follow up use of the tools

It was noted by clinicians and leaders in digital health implementation that self-directed web-based tools and modules may shift the role of the clinician from providing direct face-to-face intervention to referring people with ABI and their communication partners to the tool, and following up its use;

*“[the tool] will just become another menu option, almost, to do, so long as they [the clinicians] are aware of it, and that it's easy to incorporate. And then, if they're also aware of these other things that people can be doing, then they can just recommend it. They don't have to have headspace to follow the people in and then, you know, family members and the person with injury can pursue it if they want to, and if not, that's fine. And if it's on the clinician’s agenda, it means that they can also just say “well, how’d you go with such and such” so they can pick it up and review it again at some point, but they can also just provide that as information for people to use as they see fit.” (Speech-language pathologist 2, Focus group 4).*

Clinicians described the extent of clinician involvement as being something that could be negotiated directly with people with ABI and their close others;

*“talk together about: “How would you like us to work with that?”, “Would you like to go off and do this on your own?”, “Do you want to have joint sessions and maybe make it a team-shared equal plan?” Actually, it needs to be the person using it and their family member need to hold the power. And then they just instruct how they want help, or no help from the system, I suppose. If that's got, like, if they've got access. I mean, I'm sure there'll be plenty of people out there who'll be using it and they have no access to any part of the system at that point. But if they do have access, I think it would be great if they are able to inform their clinician how they want help through a conversation that is shared with them.” (Speech-language pathologist 2, Focus group 4).*

Developers could ask themselves from the outset of intervention design;

*“is it the clinician's role to actually police whether the person's been finishing, completing the exercises, that's something that you're probably going to have to have a position on? You might want to make that decision yourself; you might want to do that in consultation during implementation. But I think it is something that would have to be thought through.” (Digital health implementation leader 5).*

For example,

- *“there's an argument with this that if you are a clinician referring a patient to this tool, you have a responsibility to make sure that the treatment's working effectively. You can't just refer them and then go ‘well, that's just speech pathology’ or that's...” (Digital health implementation leader 5).*
- *“there's an expectation of both sides of the coin, right? There's an expectation from the consumer that the clinician takes it seriously therefore they'll do it. But then there has to be an expectation from consumer that the clinician is going to engage with it.” (Digital health implementation leader 4).*

This responsibility therefore comes from Strategy 3.3 in which speech-language pathologists provide initial referral.

##### Consideration 4J – Need to adjust from face-to-face delivery

Changing from face-to-face to web-based delivery could be a significant adjustment for some clinicians. Leaders in digital health implementation recognized that;

- *“the healthcare provider aspect is a different way of working for them. They’re used to going out and seeing people face-to-face. They need to be rather skilled in using technology to navigate issues that they might be having. They need to be more skilled in establishing relationships. It is very much a change for some healthcare providers from being on their feet all day, seeing people all day, to sitting at a desk in front of a computer screen all day. That’s a significant change as well. They can certainly all be overcome but there are some changes that would need to be considered and could potentially impact on the use of virtual care.” (Digital health implementation leader 8).*
- *“from a clinician's perspective, anything new, it's that whole you know, ‘we've always done it this way, why do we need to make changes?’, the whole change management aspect is always the most challenging part of anything new. So, if there's a significant change to how they're going to deliver their education or how they're going to need to structure their appointments, whether they're going to need to learn a new system, I think all of those things will have a strong impact on the clinician engaging.” (Digital health implementation leader 6).*

###### Strategy 4.12 – Provide a checklist for web-based service delivery

Clinicians may benefit from receiving a guide to web-based service delivery;

*“you could potentially look at putting together guidelines or tips which would make it easier for people to use or deliver virtual care. […] Some items to consider when delivering virtual care includes; taking regular breaks, making sure you have a really good workspace set up, that you have access to other care providers if you have a difficult patient or challenging patient that you need to debrief about. Those types of things are quite important. Also consider that they have a quiet space to work in, no disruptions – that’s often a very difficult thing sometimes when delivering virtual care. So, I think some sort of checklist of things they should consider before delivering virtual care would probably help them to overcome some of those challenges and barriers.” (Digital health implementation leader 8).*

##### Consideration 4K – Need to provide technological support to people with ABI and close others

It was recognized that;

- *“there's probably a tech support element to these models of care from the clinician's end. [There is a] Good chance that the first point of call that will be made from the patient will be the person that referred them to the application.” (Digital health implementation leader 5).*
- *“For learning how to use the platform, I think if they're working with the clinician, then clinicians will, I think, provide support around using the platform based on the person’s need.” (Speech-language pathologist 1, Focus group 6).*

However, speech-language pathologists noted;

*“I would need training of course though to see also about troubleshooting. How can I fix problems, especially for that I would need it, training.” (Speech-language pathologist 5, Focus group 1).*

###### Strategy 4.13 – Provide troubleshooting education and support for clinicians

Given clinicians may need to learn how to provide technological support,

- *“That may mean that there will need to be some basic trouble shooting education for that.” (Digital health implementation leader 5).*
- *“Might be quite short, mightn’t it – [troubleshooting training] doesn't need to be massive.” (Speech-language pathologist 3, Focus group 1).*

In addition to education, there may need real-time support;

*“Working with this tool and alone, you might be a little bit lost because sometimes even you're trained in everything. So, there should be an IT [information technology] personnel available at all times if possible,” (Speech-language pathologist 5, Focus group 1).*

This support and education may therefore need to complement the introductory tutorial for clinicians in supporting digital literacy (Consideration 2L) and the learning of unfamiliar processes (2M).

##### Consideration 4L – Area of clinical practice

Given the breadth of practice and specializations in the profession, a speech-language pathologist noted that it should not be assumed that all speech-language pathologists are familiar and confident in supporting people with ABI and their close others;

*“I think this is an area, communication partner training and cog[nitive]-comm[unication] treatment in - Very, I would say a lot of clinicians don't really feel comfortable doing it, so training days and things I think would be really helpful for this.” (Speech-language pathologist 1, Focus group 6).*

###### Strategy 4.14 – Provide clinical professional development in the program

Therefore, in addition to software tutorials, clinicians could be supported through clinical professional development in the program. A participant in the same focus group noted that treatment fidelity was desirable from the communication partner perspective;

*“I agree with [Speech-language pathologist 1, Focus group 6]'s point about maybe a day’s training, half a day training, so it can be delivered in the best possible way.” (Communication partner 5, Focus group 6).*

However, an expert in digital health noted that the feasibility of the training package should be considered;

*“clinical groups may say they need training, but their availability to complete that training's probably non-existent. It's kind of like a ‘training lite’ approach might be required.” (Digital health implementation leader 5).*

### Abbreviations

ABI: acquired brain injury

NASSS: Nonadoption, Abandonment, Scale-up, Spread, and Sustainability

NSW: New South Wales

TBI: traumatic brain injury

UTS: University of Technology Sydney

GP: general practitioners

IT: information technology

LSVT: Lee Silverman Voice Treatment

MBS: Medicare Benefits Schedule

MOOC: Massive Open Online Course

NDIS: Australia’s National Disability Insurance Scheme

PPE: personal protective equipment

SLP: speech-language pathologists

UK: United Kingdom
